# Supplementary material for: Dominant negative ATP5F1A variants disrupt oxidative phosphorylation causing neurological disorders
Source: EMBO Mol Med. 2025 Aug 26;17(10):2562–85. doi: 10.1038/s44321-025-00290-8 (PMC12514044; doi:10.1038/s44321-025-00290-8)
Supplement: Supplementary file 1 — Appendix [file 44321_2025_290_MOESM1_ESM.pdf]

APPENDIX

Dominant negative ATP5F1A variants disrupt oxidative phosphorylation causing neurological disorders

(Fielder et al.)

Table of contents

1. Appendix Full Clinical Report (pages 2 – 5)
2. Appendix Figure S1 – MRI images (page 6)
3. Appendix Figure S2 – ATP5F1A Sequence alignments (page 7)
4. Appendix Figure S3 – *atp-1* extra copy expression (page 8)
5. Appendix Figure S4 – Thrashing speed (page 9)
6. Appendix Figure S5 – Mitochondrial respiration - Seahorse analysis (page 10)
7. Appendix Figure S6 – Mechanism model (page 11)
8. Appendix Figure S7 – Crossing scheme (page 12)
9. Appendix Table S1 – CRISPR reagents (page 13)
10. Appendix Table S2 – Respiratory chain enzyme activities (page 14)
11. Appendix Table S3 – *C. elegans* lines used in study (page 15)
12. Appendix Table S4 – Primers for *atp-1* extra copy (page 16)
13. Appendix Table S5 – Biological and technical replicates (page 17)
14. Appendix Undiagnosed Diseases Network Consortium Author List (pages 18-25)

APPENDIX – FULL CLINICAL REPORT

Proband 1 is a 12-year-old male with dystonia, global developmental delay since birth, and mild-moderate intellectual disability. A diagnosis of cerebral palsy was made at three years of age. He crawled up to age three, and then he walked with the aid of braces. His first words were at 18 months. Neuropsychiatric testing placed him at a 6-year-old level at a chronologic age of 10 years. His examination is significant for dystonia, spasticity, gait apraxia, fluency disorder, poor fine and gross motor skills, limited attention, poor muscle tone, and brisk reflexes with normal sensation, but he is nondysmorphic. He has had a normal EEG, normal brain/spine magnetic resonance imaging (MRI), and normal EMG/NCV studies. Blood lactic acid levels have been mildly elevated; CSF lactate was normal. GDF15 levels were normal. Family history was non-contributory. Clinical duo exome sequencing with the father identified a heterozygous missense variant in *ATP5F1A* (NM_004046.6:c.545G>A p.(Arg182Gln)). Subsequent targeted maternal studies confirmed the variant was *de novo*. Trio genome sequencing did not identify a second *ATP5F1A* variant, and mitochondrial DNA sequencing was normal.

Proband 2 is a 9-year-old male with moderate to severe global developmental delay. He was born at term via forceps after an uncomplicated pregnancy and was in good condition at birth. Early history included significant central hypotonia with crawling achieved at 18 months of age and pulling to stand by 16 months. He can walk with support, but independent ambulation has not been achieved. He has reduced tone with brisk reflexes in upper and lower limbs and upgoing plantar responses. He has a tented upper lip with hypotonic facies and profound central hypotonia. He displays prominent drooling but there have been no concerns regarding swallowing or possible aspiration. Verbal communication is limited to a handful of words, however comprehension appears better than expressive language. There has been no regression nor seizures. Growth is in the normal range. Serum lactate was borderline at 2.2 mM (NR 0.5-1.4 mM). Genetic testing including chromosome microarray, fragile X testing, trio exome sequencing and *DMPK* repeat analysis was non diagnostic. Trio genome sequencing identified a recurrent *de novo* heterozygous missense variant in *ATP5F1A* (NM_004046.6:c.545G>A p.(Arg182Gln)). Mitochondrial genome sequencing was non diagnostic.

Proband 3 is a 3-year-old female with a significant history of severe global developmental delay and abnormal muscle tone, characterized by central hypotonia and

peripheral spasticity. Her medical history is further complicated by dysphagia, chronic constipation, frequent vomiting, and feeding difficulties necessitating the placement of a gastrostomy tube (G-tube). Despite her mother's early concerns about feeding difficulties since birth, she was not evaluated until 7 months of age, following a drop in her weight to the 1st percentile, which prompted the decision for G-tube placement. She had a mildly elevated lactate 3.1 mM (normal 0.5-2.2), which at other times were normal. Genetic testing, including chromosomal microarray analysis (CMA) and mitochondrial DNA analysis were unrevealing. Subsequent clinical exome sequencing identified a heterozygous variant of uncertain significance (NM_004046.6:c.1037C>T p.(Ser346Phe)) in the *ATP5F1A* gene. It also identified a single heterozygous pathogenic variant in the *MMAA* (NM_172250.3:c.433C>T p.(Arg145Ter)) gene, but biochemical testing had been normal and did not support the autosomal recessive methylmalonic aciduria diagnosis. Subsequent testing confirmed that the *ATP5F1A* variant in this patient is *de novo*.

Proband 4 was born prematurely at 33 weeks gestation. At age 1 year, developmental delays were noted. Over the next years, she gradually developed spasticity and dystonia, and could only walk with adaptive devices. On exam, she has dystonia most notable in facial and upper extremity movements. She has severe spasticity, most pronounced in her lower extremities, and has contractures in elbows and knees. Her functionality improved following dorsal rhizotomy. She has fatiguability. She is fed by gastrostomy tube for dysphagia and has severe gastroesophageal reflux. At age 13 years, she has growth retardation with height at -4 SD and weight at -3.3 SD. She is very socially interactive using assistive devices. Her lactate and her GDF15 levels were normal. She had a normal oxidized/reduced glutathione ratio. However, while non-fasting, she has elevated ketones with 3-hydroxybutyrate 1.96 mM, and acetoacetate 0.50 mM, with a clear elevated ratio of 3.89 (normal 0.47-2.57). Genetic testing showed a de novo variant in *ATP5F1A* (NM_004046.6:c.1037C>T p.(Ser346Phe)).

Proband 5 is a 16-year-old female with significant intellectual disability, developmental regression, growth failure, and history of hospitalizations for ketoacidosis. Prenatally, she was noted to have a dilated right ventricle in her brain, thought to be due to an ischemic event. Neonatally, she had hyperbilirubinemia, which improved with phototherapy. She had failure to thrive, multiple epistaxis which improved with surgery and cautery, an atrial septal defect, sinus tachycardia with normal QTc, mild scoliosis, and she is not dysmorphic. She had one possible seizure event at 7 years of age. Developmental concerns were raised at 6 months of age. She

walked at 21 months with stiffness and toe walking, which has improved. She has lost skills: she had about five words, which she lost around age 4 years, and she is currently nonverbal. She had poor social interaction at around 3-4 years of age, but has improved. Behaviorally, she shows stereotypic hand movements and teeth grinding, and she had abusive behaviors in the past. Treatment with fluoxetine has improved anxiety and crying. On neurologic exam she has asymmetric increased tone on the left side, 3+ reflex on the left patella, ankle clonus on the left, and abnormal gait. She has had 8 hospitalizations with ketoacidosis (3-hydroxybutyrate 3.6 mM), typically with normal ammonia, with the first episode at 4 years of age with PICU admission. Her ketones normalize when she is healthy. She also has intermittent elevations of blood (1.1-5.5 mM) with normal lactate:pyruvate ratio, and urine lactate without a clinical correlate. She has never had documented hypoglycemia but has been hyperglycemic. Brain MRI has shown stable asymmetry of lateral ventricles and stable focal volume loss. GDF15 levels were normal. Skin fibroblast studies for beta ketothiolase, succinyl CoA:3-oxoacid CoA transferase (SCOT), and pyruvate carboxylase (PC) were normal. Clinical trio exome sequencing for proband 5 identified a *de novo* missense variant in *ATP5F1A* (NM_004046.6:c.992C>T p.(Pro331Leu)). Additionally, a *de novo FLT1* consensus splice variant (NM_002019.4:c.1276+2T>C) was identified; this was considered as a potential candidate for her prenatal stroke, given the role of FLT1 (also known as VEGFR1) in vascular development (Chappell et al., 2013; Nesmith et al., 2017). Compound heterozygous variants in *IARS1* were also identified (paternally inherited NM_002161.6:c.2500G>A p.(Val834Met) and maternally inherited NM_002161.6:c.3713C>T p.(Thr1238Ile)). Pathogenic variants in *IARS1* cause growth retardation, impaired intellectual development, hypotonia, and hepatopathy (OMIM #617093). However, zinc deficiency is a common feature of this condition, and the proband had normal zinc levels, and the p.Thr1238Ile variant has been classified as likely benign in ClinVar (variation 744089), so this was not prioritized as a possible contributor (Kopajtich et al., 2016). Finally, a maternally inherited *PCK2* variant (NM_004563.4:c.1405C>T p.(Arg469Cys)) was identified. This was considered as a candidate contributory factor for her ketoacidosis, since recessive *PCK2* variants cause mitochondrial phosphoenolpyruvate carboxykinase deficiency (OMIM #261650); however, a second variant in this gene was not identified. Mitochondrial DNA testing identified one rare homoplasmic *CYTB* variant, m.15099T>C, which has been classified as likely benign in ClinVar (variation 693824), and thus ruled out as contributory.

Proband 6 is an 11-year-old male with global developmental delay, limited verbal communication, postnatal failure to thrive with feeding difficulties, axial hypotonia, and leg

spasticity with contractures (and therefore is nonambulatory). Echocardiogram and brain MRI were normal. Metabolic workup, including lactate, ammonia, acylcarnitine profile, and plasma amino acids, was largely unremarkable. Targeted genetic testing, including chromosomal microarray analysis, spinal muscular atrophy testing, ataxia panel, Fragile X testing, and Prader-Willi methylation studies were nondiagnostic. Mitochondrial genome sequencing revealed a 22% heteroplasmic variant of uncertain significance in the gene MT-TI (m.4317A>G), inherited from his mother who has 9% heteroplasmy in blood; this variant is not considered contributory to his phenotype. Trio whole exome sequencing and later whole genome sequencing identified a *de novo* heterozygous missense variant in *ATP5F1A* (NM_004046.6:c.326T>C p.(Leu109Ser)).

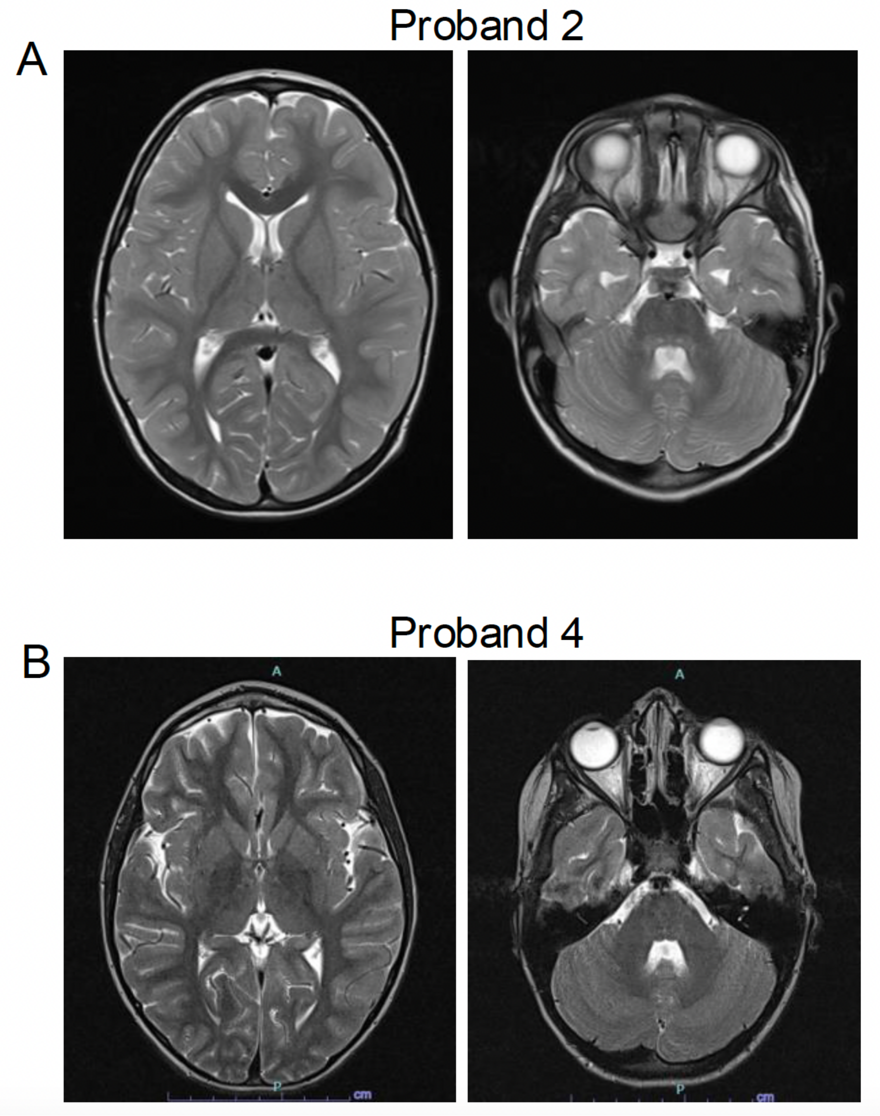

Appendix Figure S1 – Magnetic Resonance Imaging (MRI) proband brains. T2 weighted MRI images of basal ganglia (left) and brainstem at mid pons level (right) showing normal signals for (A) proband 2 (age 29 months), and (B) proband 4 (age 8 years 11 months). MRI images of other probands were performed by outside hospitals and were not available for inclusion in this study. However, clinical records indicated that they were normal.

```
Human         1 MLSVRVAAAVVRALP-RRAGL--VSRNALGSSFIAARNFHASNTHLQKTG   47
                |||:|:  |.  |.  . ||:   .|   |.|
C. elegans    1 MLSKRIVTALNTAVKVQNAGIATTARGMAGAS------------------   32

Human        48 TAEMSSILEERILGADTSVDLEETGRVLSIGDGIARVHGLRNVQAEEMVE   97
                 .|.| |||||||| :| :.|||||:|||||||||||:||:|:|||||||
C. elegans   33 GSEVSKILEERILGTETGINLEETGKVLSIGDGIARVYGLKNIQAEEMVE   82

Human        98 FSSGLKGMSLNLEPDNVGVVVFGNDKLIKEGDIVKRTGAIVDVPVGEELL  147
                | ||:|||.:||: ||||||||||||.|:|||||||||||||||||: ||
C. elegans   83 FDSGIKGMAMNLDVDNVGVVVFGNDKVIREGDIVKRTGAIVDVPVGDGLL  132

Human       148 GRVVDALGNAIDGKGPIGSKTRRRVGLKAPGIIPRISVREPMQTGIKAVD  197
                ||||||||| |||||||    | || .||||||||:|||||| ||:||||
C. elegans  133 GRVVDALGNPIDGKGPIANARRSRVEVKAPGIIPRLSVREPMVTGVKAVD  182

Human       198 SLVPIGRGQRELIIGDRQTGKTSIAIDTIINQKRFNDGSDEKKKLYCIYV  247
                ||||||||||||||||||||||.||||||||||||||  |:||||:||||
C. elegans  183 SLVPIGRGQRELIIGDRQTGKTAIAIDTIINQKRFNDAGDDKKKLFCIYV  232

Human       248 AIGQKRSTVAQLVKRLTDADAMKYTIVVSATASDAAPLQYLAPYSGCSMG  297
                |:|||||||||:||||||| || ||||||||||||||||:|||||||.||
C. elegans  233 AVGQKRSTVAQIVKRLTDAGAMDYTIVVSATASDAAPLQFLAPYSGCAMG  282

Human       298 EYFRDNGKHALIIYDDLSKQAVAYRQMSLLLRRPPGREAYPGDVFYLHSR  347
                |:|||||||||||:||||||||||||||||||||||||||||||||||||
C. elegans  283 EHFRDNGKHALIIFDDLSKQAVAYRQMSLLLRRPPGREAYPGDVFYLHSR  332

Human       348 LLERAAKMNDAFGGGSLTALPVIETQAGDVSAYIPTNVISITDGQIFLET  397
                |||||||||.. ||||||||||||||||||||||||||||||||||||||
C. elegans  333 LLERAAKMNNSLGGGSLTALPVIETQAGDVSAYIPTNVISITDGQIFLET  382

Human       398 ELFYKGIRPAINVGLSVSRVGSAAQTRAMKQVAGTMKLELAQYREVAAFA  447
                ||||||:|||||||||||||||||||:|||||||:|||||||||||||||
C. elegans  383 ELFYKGVRPAINVGLSVSRVGSAAQTKAMKQVAGSMKLELAQYREVAAFA  432

Human       448 QFGSDLDAATQQLLSRGVRLTELLKQGQYSPMAIEEQVAVIYAGVRGYLD  497
                ||||||||.||||| |||||||||||||| || ||||| ||||||:||||
C. elegans  433 QFGSDLDASTQQLLNRGVRLTELLKQGQYVPMGIEEQVGVIYAGVKGYLD  482

Human       498 KLEPSKITKFENAFLSHVVSQHQALLGTIRADGKISEQSDAKLKEIVTNF  547
                |.:|| |||||  ||.|. |  |||| ||| :|.|| |:||.||::| ||
C. elegans  483 KVDPSAITKFEKEFLAHLRSSQQALLKTIREEGQISPQTDAQLKDVVVNF  532

Human       548 LAGFEA    553
                || |.
C. elegans  533 LATFKP    538
```

Appendix Figure S2 – *ATP5F1A* is highly conserved as *atp-1*. Sequence alignment of human ATP5F1A and *C. elegans* ATP-1 protein sequences. Horizontal dashes indicate gap, vertical dashes indicate identity, colon indicates conservative change, blank indicates mismatch. Wild type proband variant residues are highlighted in magenta. Annotated ATP binding sites from UniProt are underlined.

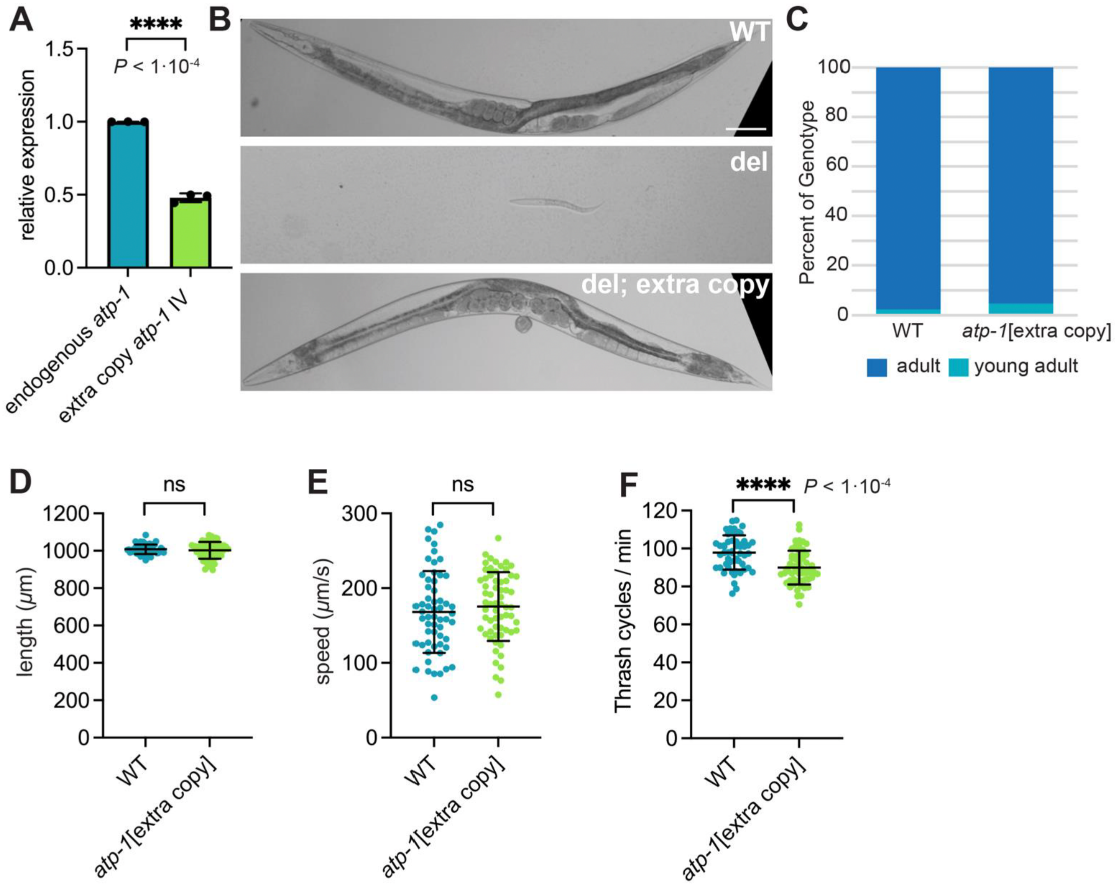

Appendix Figure S3 – Animals with extra copies of wild type *atp-1* are superficially normal. (A) Relative expression of endogenous *atp-1* compared to WT *atp-1* transgene inserted on chromosome IV as measured by RNA-seq. The transgene has two synonymous changes compared to the endogenous *atp-1*. Three biological replicates, student's t test used to compare results. Mean and SD plotted. (B) Animals 72 hours after embryo laying. WT animals grow to adults at 72 hours post embryo lay (top), while *atp-1* deletion homozygous animals arrest at larval stage L1 (middle). Growth and fertility of *atp-1* deletion animals are fully rescued by addition of two transgenic copies of *atp-1* (*atp-1[extra copy]*) (bottom). Scale bar 100 µm. Quantification of development rate (C), body length (D), crawl speed (E) and thrashing rate of wild type animals with and without extra copies of *atp-1*. Three biological replicates for development rate performed with approximately 60 animals per genotype per replicate. Three biological replicates performed with approximately 90 animals for each genotype used in recordings for length, crawl speed, and thrashing. Mean and SD plotted, student's t-test performed. ns- not significant, * p <0.05, ** p<0.01, *** p<0.001, **** p < 0.0001.

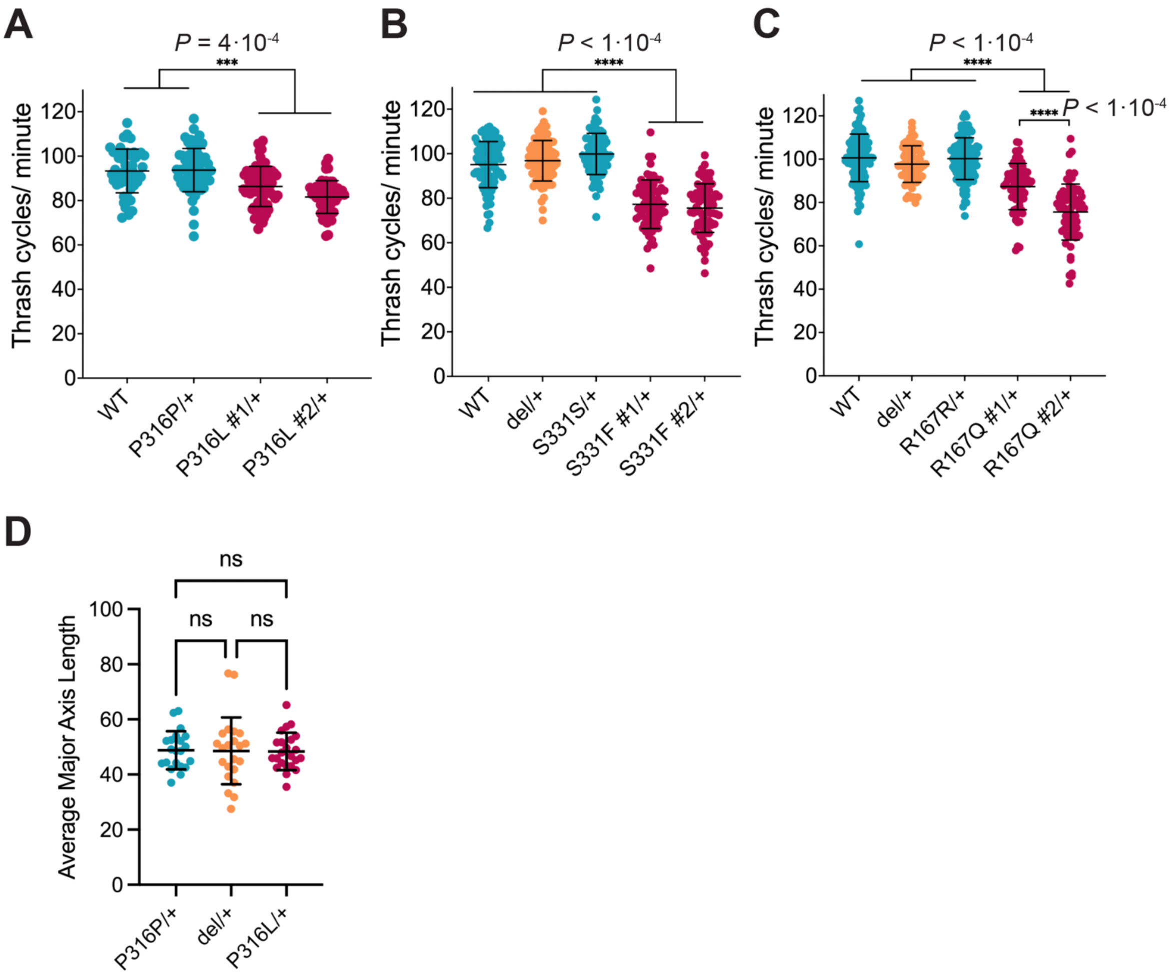

Appendix Figure S4. ***atp-1*** **proband variant animals thrash slower and P316L animals have normal mitochondrial tubule length.** (A-C) One day adult proband variant animals show slower thrashing as compared to control animals. (D) Quantification of major axis length of *C. elegans* muscle mitochondria shows no difference between heterozygous control edit, heterozygous deletion, and heterozygous P316L animals. Same images used as in Fig 5B. Mean and sd plotted. One way ANOVA performed followed by post-hoc Holm-Sidak tests. ns- not significant, * p <0.05, ** p<0.01, *** p<0.001, **** p < 0.0001.

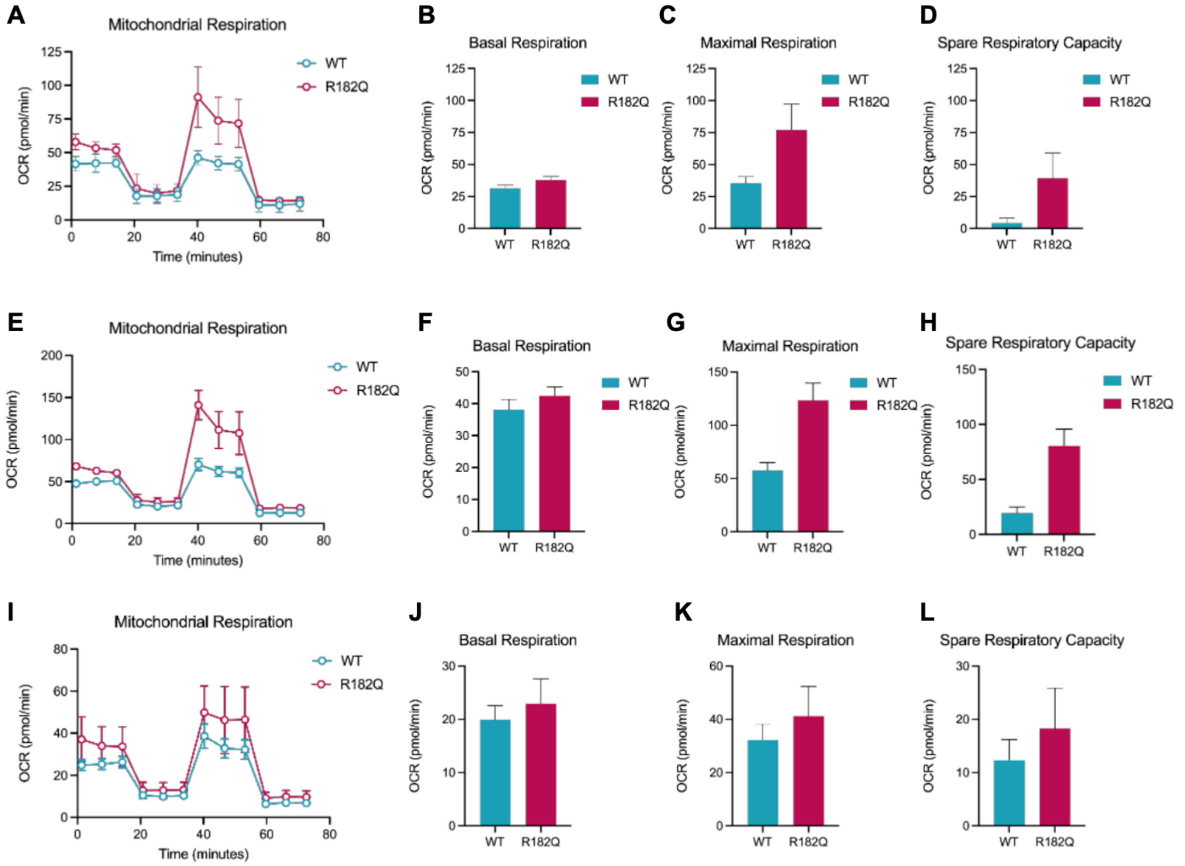

Appendix Figure S5. Individual replicates of mitochondrial stress tests performed on proband derived fibroblasts shown in Fig. 6. Three individual replicates of mitochondrial respiration studies of *ATP5F1A* p.R182Q proband fibroblasts and age, race, and sex matched control fibroblasts as measured by Seahorse. (A, E, I) Mitochondrial respiration, (B, F, J) Basal, (C, G, K) maximal, and (D, H, L) spare oxygen consumption rates (OCR).

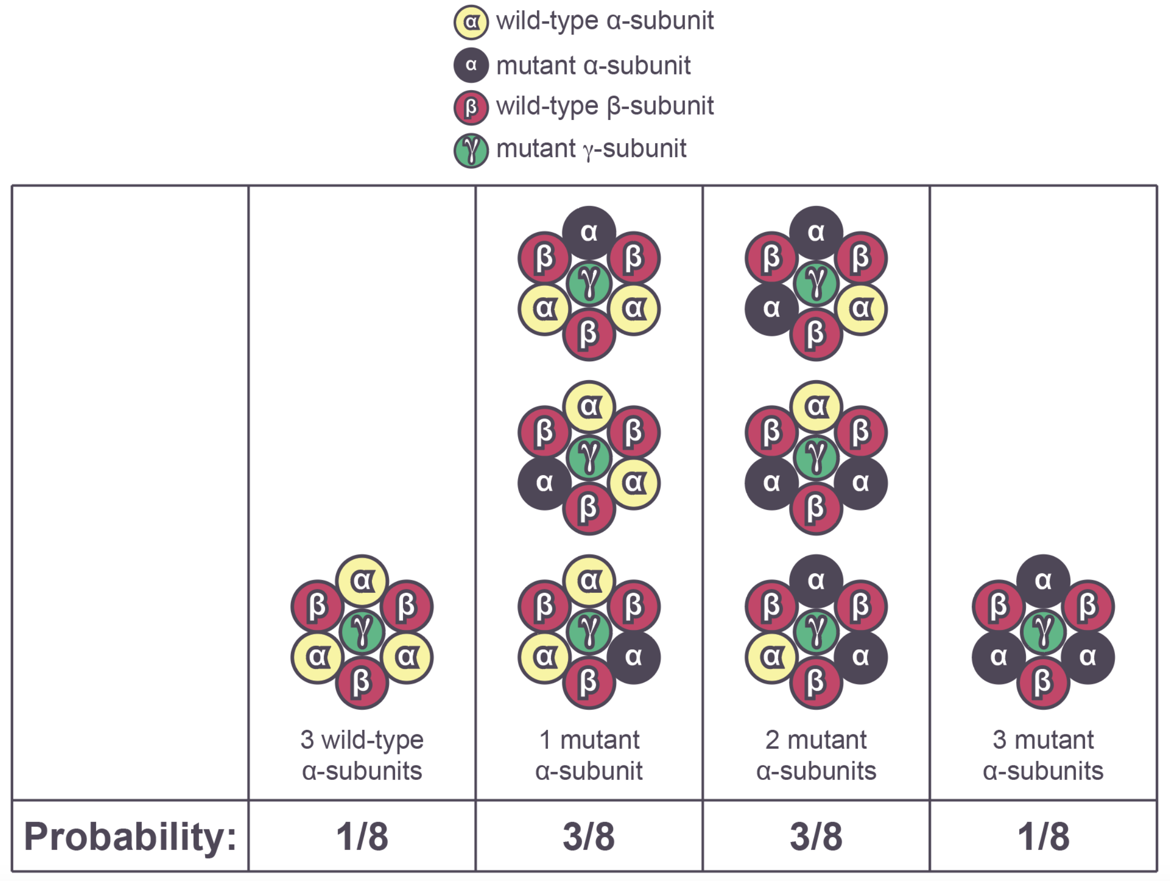

Appendix Figure S6. Possible combinations of α- and β-subunit assembly in F_1 complex. The α-and β-subunits form hetero-hexameric ring around the central stalk (γ-subunit). 1/8th of the complex formed will have 3 wild-type α-subunits. 3/8th will have 1 mutant and two wild-type α-subunits. 3/8th will have 2 mutant and 1 wild-type α-subunits. 1/8th will have 3 mutant α-subunits.

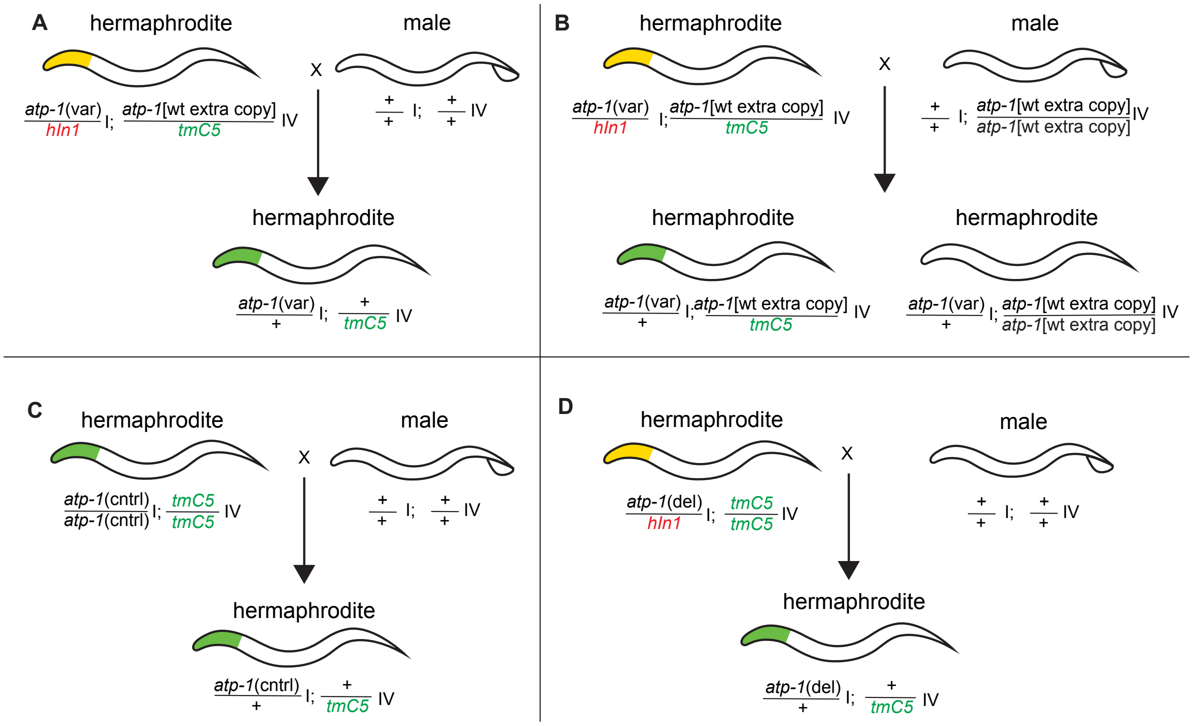

Appendix Figure S7. Crossing schematic to generate animals analyzed in this study. Animals heterozygous for the *hIn1* balancer have red heads (pharynx), animals heterozygous for *tmC5* balancer have green heads (pharynx). + indicates locus with no transgene. Animals homozygous for either balancer are uncoordinated. (A) heterozygous proband variant animals with one extra copy that had red and green heads (depicted as yellow) were crossed with WT males to obtain heterozygous animals with no extra copies that were used in Figs. 3, 4, and Appendix Fig. S4. (B) Heterozygous proband variant animals with one extra copy that had red and green heads were crossed with extra copy males to obtain heterozygous animals with one extra copy and heterozygous animals with two extra copies that were used in Fig. 3. (C) Animals homozygous for the control edits or WT and for the *tmC5* balancer were mated to WT males to obtain control edit animals and WT animals in Figs. 3, 4, and Appendix Fig. S4. (D) Animals heterozygous for *atp-1* deletion and homozygous for the *tmC5* balancer were crossed with WT males to obtain deletion heterozygotes in Figs. 3, 4, and Appendix Fig. S4.

Appendix Table S1A. Guide sequences used for CRISPR/Cas9 editing

Locus	Guide RNA sequence used for CRISPR/Cas9 editing
R167	CAAACGTGGGATGATTCCTG
S331	GGCGGCACGTTCGAGAAGAC
P316	TCGTCCACCAGGTCGTGAGG

Appendix Table S1B. Repair templates used for CRISPR/Cas9 editing

Name	Restriction site change	single stranded DNA repair template
R167Q repair template	FokI site removed	CCAACGCCAGAAGATCTCGTGTCGAAGTCAAGG<br>CaCCAGGAAT*CATtC*CA**Caa**TTGTCTGTGCGCG<br>AACCAATGGTTACTGGAGTCAAGGCCGTCGATT
R167R repair template	FokI site removed	CCAACGCCAGAAGATCTCGTGTCGAAGTCAAGG<br>CaCCAGGAAT*CATtC*CA**CGT**TTGTCTGTGCGCG<br>AACCAATGGTTACTGGAGTCAAGGCCGTCGATT
S331F repair template	XbaI site added	AGGTCGTGAGGCTTACCCAGGAGATGTCTTCTA<br>CCTTCAC**Ttt**CGTCT*TCTaGA*ACGTGCCGCCAA<br>GATGAACAACAGCCTCGGAGGAGGATCTCTCAC
S331S repair template	XbaI site added	AGGTCGTGAGGCTTACCCAGGAGATGTCTTCTA<br>CCTTCAC**TCt**CGTCT*TCTaGA*ACGTGCCGCCAA<br>GATGAACAACAGCCTCGGAGGAGGATCTCTCAC<br>C
P316L repair template	HaeIII site added	AAGCAAGCCGTCGCCTACCGTCAAATGTCTCTT<br>CTTCTtCGTCGT**CtA**CCA*GGcC*GTGAGGCTTAC<br>CCAGGAGATGTCTTCTACCTTCACTCCCGTCT
P316P repair template	HaeIII site added	AAGCAAGCCGTCGCCTACCGTCAAATGTCTCTT<br>CTTCTtCGTCGT**CCA**CCA*GGcC*GTGAGGCTTAC<br>CCAGGAGATGTCTTCTACCTTCACTCCCGTCT

lowercase indicates changed from WT sequence
underline indicates modified PAM
bold indicates residue of interest
italics indicates altered restriction site
Changes to the restriction site were silent and did not change the amino acid sequence

Appendix Table S2. Respiratory chain enzyme activities

Proband 1 p.Arg182Gln	**Activity (controls)**	**Z-score**	**Ratio /CS**	**Z-score**	**Ratio/complex II**	**Z-score**
Complex I	91.0 (49.3-131.1)	0.2	256 (145-396)	0.5	373 (237-754)	-0.1
Complex II	243.9 (130.9-364.4)	0.3	687 (297-863)	0.6	NA	NA
Complex III	17.2 (8-29.2)	0.5	48 (19-65)	0.7	70 (36-114)	0.4
Complex II-III	133.1 (61.8-158.8)	0.8	375 (131-376)	1.2	546 (263-1100)	0.2
Complex IV	2.1 (2.2-7.1)	-1.9	6 (6-23)	-2	9 (12-35)	-2.1
Citrate synthase	355.1 (253.5-554.1)	-0.3	NA	NA	NA	NA
**Complex V hydrolysis**	**131.6 (205.8-440.6) % of avg: 42%**	**-2.76**				

Proband 4 p.Ser346Phe	**Activity (controls)**	**Z-score**	**Ratio /CS**	**Z-score**	**Ratio/complex II**	**Z-score**
Complex I	114.1 (49.3-131.1)	1	241 (145-396)	0.5	425 (237-754)	0.3
Complex II	268.1 (130.9-364.4)	0.6	566 (297-863)	0	NA	NA
Complex III	11.3 (8-29.2)	-0.5	24 (19-65)	-0.7	42 (36-114)	-0.7
Complex II-III	13.2 (61.8-158.8)	0.8	286 (131-376)	0.6	504 (263-1100)	0.1
Complex IV	5.6 (2.2-7.1)	0.7	12 (6-23)	0.6	21 (12-35)	0.6
Citrate synthase	473.4 (253.5-554.1)	0.7	NA	NA	NA	NA
**Complex V hydrolysis**	**104.6 (205.8-440.6) % of avg: 33%**	**-3.17**				

Proband 6 p.Leu109Ser	**Activity (controls)**	**Z-score**	**Ratio /CS**	**Z-score**	**Ratio/complex II**	**Z-score**
Complex I	155.7 (49.3-131.1)	2	467 (145-396)	2.7	725 (237-754)	1.9
Complex II	214.3 (130.9-364.4)	-0.1	643 (297-863)	0.3	NA	NA
Complex III	18.8 (8-29.2)	0.7	56 (19-65)	1	87 (36-114)	0.8
Complex II-III	150.8 (61.8-158.8)	1.2	453 (131-376)	1.8	704 (263-1100)	0.7
Complex IV	3.6 (2.2-7.1)	-0.5	11 (6-23)	-0.4	17 (12-35)	-0.5
Citrate synthase	333.3 (253.5-554.1)	-0.6	NA	NA	NA	NA
**Complex V hydrolysis**	**116.7 (205.8-440.6) % of avg: 37%**	**-2.98**				

Appendix Table S3. *C. elegans* lines used in study

Strain name	Allele name	Use	Origin
CGC105	*hIn1* [umnIs78] I	balancer for *atp-1* endogenous locus I	Genetic Toolkit project
FX30140	*tmC5* [F36H1.3(tmIs1220)] IV	balancer for *atp-1* [extra copy] on IV	Ref 1
MD4046	*bcIs80* [myo-3::mito-gfp + pRF4]	marker for mitochondria in muscle	Conradt lab, unpublished
SJ4100	*zcIs13* V [hsp-6p::gfp]	UPRmito reporter	Ref 2
VC2824	*atp-1(ok2203)* I	*atp-1* deletion	Ref 3
NM5179	*jsTi1493* [LoxP::mex-5p::FLP:SL2::mNeonGreen::rpl-28p::FRT::GFP::his-58::FRT3] IV	RMCE insertion strain- to generate udnSi40	Ref 4
UDN100612	*udnSi40* IV	atp-1 extra wildtype copy	this study
UDN100613	*atp-1(udn293)* I	R167Q variant #1	this study
UDN100614	*atp-1(udn294)* I	R167Q variant #2	this study
UDN100615	*atp-1(udn296)* I	R167R control edit	this study
UDN100616	*atp-1(udn298)* I	P316L variant #1	this study
UDN100617	*atp-1(udn299)* I	P316L variant #2	this study
UDN100618	*atp-1(udn301)* I	P316P control edit	this study
UDN100619	*atp-1(udn303)* I	S331F variant #1	this study
UDN100620	*atp-1(udn304)* I	S331F variant #2	this study
UDN100621	*atp-1(udn306)* I	S331S control edit	this study

1. Dejima K, Hori S, Iwata S, Suehiro Y, Yoshina S, Motohashi T, Mitani S. An Aneuploidy-Free and Structurally Defined Balancer Chromosome Toolkit for Caenorhabditis elegans. Cell Rep. 2018 Jan 2;22(1):232-241. doi: 10.1016/j.celrep.2017.12.024. PMID: 29298424.

2. Yoneda T, Benedetti C, Urano F, Clark SG, Harding HP, Ron D. Compartment-specific perturbation of protein handling activates genes encoding mitochondrial chaperones. J Cell Sci. 2004;117(Pt 18):4055-66.

3. C. elegans Deletion Mutant Consortium. large-scale screening for targeted knockouts in the Caenorhabditis elegans genome. G3 (Bethesda). 2012 Nov;2(11):1415-25. doi: 10.1534/g3.112.003830. Epub 2012 Nov 1. PMID: 23173093; PMCID: PMC3484672.

4. Nonet ML. Efficient Transgenesis in Caenorhabditis elegans Using Flp Recombinase-Mediated Cassette Exchange. Genetics. 2020;215(4):903-21.

Appendix Table S4. Primers used to clone wild type *atp-1* extra copy

**Primer**	**Primer sequence**
atp1_plasmid_R2	GCCAGAAAAGCTTCGCCACTAGTTCTAGAGCGGC
atp1_hifi_F21	TAGAACTAGTGGCGAAGCTTTTCTGGCGTTTTTCAGCTGA
atp1_hifi_F22	AAGTCAAGGCt*C CgGG* AATCATCCCA**CGT**TTGTCTGTG
atp1_hifi_R21	GGGATGATT*CCcG G* aGCCTTGACTTCGACACGAGATC
atp1_plasmid_F2	CTATTCGTCCTCCTCGGGTAACCACGCGTTGCATGC
atp1_hifi_R22	GTGGTTACCCGAGGAGGACGAATAGACGAGATTGC

lower case indidcates changes from original sequence
italics indicates added NciI restriction site
bold indicates R167R codon

Appendix Table S5. Biological and technical replicates

Figure 3D - Growth rates

	WT	del/ +	PP/+	SS/+	RR/+	PL/+	SF/+	RQ/+
Biological replicates	4	4	4	4	4	4	4	4
Total n	220	140	108	118	138	71	66	185

Figure 3F - Length

	WT	P316P/+	P316L #1/+	P316L #2/+
Biological replicates	3	3	3	3
Total n	59	69	66	67

	WT	del/+	S331S/+	S331F #1 /+	S331F #2/ +
Biological replicates	4	4	4	4	4
Total n	93	88	86	78	100

	WT	del/+	R167R/+	R167Q #1/+	R167Q #2/+
Biological replicate	5	3	5	5	5
Total n	132	75	120	99	115

Figure 3G - Crawl speed

	WT	P316P/+	P316L #1/+	P316L #2/+
Biological replicates	3	3	3	3
Total n	59	69	66	67

	WT	del/+	S331S/+	S331F #1/+	S331F #2/+
Biological replicates	3	3	3	3	3
Total n	66	65	60	58	75

	WT	del/+	R167R/+	R167Q #1/+	R167Q #2/+
Biological replicates	4	3	4	4	4
Total n	105	75	93	78	89

Figure 4B - hsp-4::GFP*

	WT	P316P/+	P316L #1/+	P316L #2/+	P316L #1/+; 1 extra	P316L #2/+; 1 extra
Biological replicates	4	4	4	4	4	4
Total number of wells	11	10	12	12	10	10
Total n	110	100	120	120	100	100

Figure 4C - hsp-4::GFP*

	WT	S331S/+	del/+	S331F #1/+	S331F #2/+	S331F #1/+; 1 extra	S331F #2/+; 1 extra	S331F #1/+; 2 extra	S331F #2/+; 2 extra
Biological replicates	4	4	3	4	4	4	3	4	3
Total number of wells	12	12	8	12	12	9	7	9	7
Total n	120	120	80	120	120	90	70	90	70

Figure 4D - hsp-4::GFP*

	WT	R167R/+	del/+	R167Q #1/+	R167Q #2/+	R167Q #1/+; 1 extra	R167Q #2/+; 1 extra	R167Q #1/+; 2 extra	R167Q #2/+; 2 extra
Biological replicates	3	3	3	2	3	3	3	3	3
Total number of wells	9	9	8	5	6	8	7	8	8
Total n	90	90	80	50	60	80	70	80	80

* Some wells were censored either because animals were out of focus or had autofluorescent lint particles that interfered with measurements
Additionally, R167Q/+ animals are extremely sick and difficult to work with. As such, they only two biological replicates are available for the R167Q/+ genotype.

Figure 5B - Mito morphology

Average area		P316P	deletion /+	P316L /+
	Biological replicates	3	3	3
	Total n	21	22	23

Average minor axis		P316P	deletion /+	P316L /+
	Biological replicates	3	3	3
	Total n	21	22	23

Undiagnosed Diseases Network Consortium Author List

**Full Name**	**Affiliation**
Alyssa A. Tran	Baylor College of Medicine, Clinical site
Arjun Tarakad	Baylor College of Medicine, Clinical site
Ashok Balasubramanyam	Baylor College of Medicine, Clinical site
Brendan H. Lee	Baylor College of Medicine, Clinical site
Carlos A. Bacino	Baylor College of Medicine, Clinical site
Daryl A. Scott	Baylor College of Medicine, Clinical site
Elaine Seto	Baylor College of Medicine, Clinical site
Gary D. Clark	Baylor College of Medicine, Clinical site
Hongzheng Dai	Baylor College of Medicine, Clinical site
Hsiao-Tuan Chao	Baylor College of Medicine, Clinical site
Ivan Chinn	Baylor College of Medicine, Clinical site
James P. Orengo	Baylor College of Medicine, Clinical site
Jennifer E. Posey	Baylor College of Medicine, Clinical site
Jill A. Rosenfeld	Baylor College of Medicine, Clinical site
Kim Worley	Baylor College of Medicine, Clinical site
Lindsay C. Burrage	Baylor College of Medicine, Clinical site
Lisa T. Emrick	Baylor College of Medicine, Clinical site
Lorraine Potocki	Baylor College of Medicine, Clinical site
Monika Weisz Hubshman	Baylor College of Medicine, Clinical site
Richard A. Lewis	Baylor College of Medicine, Clinical site
Ronit Marom	Baylor College of Medicine, Clinical site
Seema R. Lalani	Baylor College of Medicine, Clinical site
Shamika Ketkar	Baylor College of Medicine, Clinical site
Tiphanie P. Vogel	Baylor College of Medicine, Clinical site
William J. Craigen	Baylor College of Medicine, Clinical site
Jared Sninsky	Baylor College of Medicine, Clinical site
Lauren Blieden	Baylor College of Medicine, Clinical site
Sandesh Nagamani	Baylor College of Medicine, Clinical site
Hugo J. Bellen	Baylor College of Medicine, Model Organism Screening Center
Michael F. Wangler	Baylor College of Medicine, Model Organism Screening Center
Oguz Kanca	Baylor College of Medicine, Model Organism Screening Center
Shinya Yamamoto	Baylor College of Medicine, Model Organism Screening Center
Christine M. Eng	Baylor College of Medicine, Sequencing Center
Patricia A. Ward	Baylor College of Medicine, Sequencing Center
Pengfei Liu	Baylor College of Medicine, Sequencing Center
Adeline Vanderver	Children's Hospital of Philadelphia
Cara Skraban	Children's Hospital of Philadelphia
Edward Behrens	Children's Hospital of Philadelphia
Gonench Kilich	Children's Hospital of Philadelphia
Kathleen Sullivan	Children's Hospital of Philadelphia
Kelly Hassey	Children's Hospital of Philadelphia
Ramakrishnan Rajagopalan	Children's Hospital of Philadelphia
Rebecca Ganetzky	Children's Hospital of Philadelphia
Vishnu Cuddapah	Children's Hospital of Philadelphia

Anna Raper	Children's Hospital of Philadelphia, University of Pennsylvania
Daniel J. Rader	Children's Hospital of Philadelphia, University of Pennsylvania
Giorgio Sirugo	Children's Hospital of Philadelphia, University of Pennsylvania
Vaidehi Jobanputra	Columbia University
Allyn McConkie-Rosell	Duke University
Kelly Schoch	Duke University
Mohamad Mikati	Duke University
Nicole M. Walley	Duke University
Rebecca C. Spillmann	Duke University
Vandana Shashi	Duke University
Alan H. Beggs	Harvard University
Calum A. MacRae	Harvard University
David A. Sweetser	Harvard University
Deepak A. Rao	Harvard University
Edwin K. Silverman	Harvard University
Elizabeth L. Fieg	Harvard University
Frances High	Harvard University
Gerard T. Berry	Harvard University
Ingrid A. Holm	Harvard University
J. Carl Pallais	Harvard University
Joan M. Stoler	Harvard University
Joseph Loscalzo	Harvard University
Lance H. Rodan	Harvard University
Laurel A. Cobban	Harvard University
Lauren C. Briere	Harvard University
Matthew Coggins	Harvard University
Melissa Walker	Harvard University
Richard L. Maas	Harvard University
Susan Korrick	Harvard University
Jessica Douglas	Harvard University
Cecilia Esteves	Harvard University, Data Management and Coordinating Center (DMCC)
Emily Glanton	Harvard University, Data Management and Coordinating Center
Isaac S. Kohane	Harvard University, Data Management and Coordinating Center
Kimberly LeBlanc	Harvard University, Data Management and Coordinating Center
Rachel Mahoney	Harvard University, Data Management and Coordinating Center
Shamil R. Sunyaev	Harvard University, Data Management and Coordinating Center
Shilpa N. Kobren	Harvard University, Data Management and Coordinating Center
Brett H. Graham	Indiana University
Erin Conboy	Indiana University
Francesco Vetrini	Indiana University
Kayla M. Treat	Indiana University
Khurram Liaqat	Indiana University
Lili Mantcheva	Indiana University
Stephanie M. Ware	Indiana University
Breanna Mitchell	Mayo Clinic

Brendan C. Lanpher	Mayo Clinic
Devin Oglesbee	Mayo Clinic
Eric Klee	Mayo Clinic
Filippo Pinto e Vairo	Mayo Clinic
Ian R. Lanza	Mayo Clinic
Kahlen Darr	Mayo Clinic
Lindsay Mulvihill	Mayo Clinic
Lisa Schimmenti	Mayo Clinic
Queenie Tan	Mayo Clinic
Surendra Dasari	Mayo Clinic
Abdul Elkadri	Medical College of Wisconsin, Central Wisconsin
Brett Bordini	Medical College of Wisconsin, Central Wisconsin
Donald Basel	Medical College of Wisconsin, Central Wisconsin
James Verbsky	Medical College of Wisconsin, Central Wisconsin
Julie McCarrier	Medical College of Wisconsin, Central Wisconsin
Michael Muriello	Medical College of Wisconsin, Central Wisconsin
Michael Zimmermann	Medical College of Wisconsin, Central Wisconsin
Adriana Rebelo	University of Miami
Carson A. Smith	University of Miami
Deborah Barbouth	University of Miami
Guney Bademci	University of Miami
Joanna M. Gonzalez	University of Miami
Kumarie Latchman	University of Miami
LéShon Peart	University of Miami
Mustafa Tekin	University of Miami
Nicholas Borja	University of Miami
Stephan Zuchner	University of Miami
Stephanie Bivona	University of Miami
Willa Thorson	University of Miami
Herman Taylor	Morehouse School of Medicine, DMCC
Andrea Gropman	National Institutes of Health, Undiagnosed Diseases Program
Barbara N. Pusey Swerdzewski	National Institutes of Health, Undiagnosed Diseases Program
Camilo Toro	National Institutes of Health, Undiagnosed Diseases Program
Colleen E. Wahl	National Institutes of Health, Undiagnosed Diseases Program
Donna Novacic	National Institutes of Health, Undiagnosed Diseases Program
Ellen F. Macnamara	National Institutes of Health, Undiagnosed Diseases Program
John J. Mulvihill	National Institutes of Health, Undiagnosed Diseases Program
Maria T. Acosta	National Institutes of Health, Undiagnosed Diseases Program
Precilla D'Souza	National Institutes of Health, Undiagnosed Diseases Program
Valerie V. Maduro	National Institutes of Health, Undiagnosed Diseases Program
Ben Afzali	National Institutes of Health, Undiagnosed Diseases Program, NHGRI
Ben Solomon	National Institutes of Health, Undiagnosed Diseases Program, NHGRI
Cynthia J. Tifft	National Institutes of Health, Undiagnosed Diseases Program, NHGRI
David R. Adams	National Institutes of Health, Undiagnosed Diseases Program, NHGRI
Elizabeth A. Burke	National Institutes of Health, Undiagnosed Diseases Program, NHGRI

Francis Rossignol	National Institutes of Health, Undiagnosed Diseases Program, NHGRI
Heidi Wood	National Institutes of Health, Undiagnosed Diseases Program, NHGRI
Jiayu Fu	National Institutes of Health, Undiagnosed Diseases Program, NHGRI
Joie Davis	National Institutes of Health, Undiagnosed Diseases Program, NHGRI
Leoyklang Petcharet	National Institutes of Health, Undiagnosed Diseases Program, NHGRI
Lynne A. Wolfe	National Institutes of Health, Undiagnosed Diseases Program, NHGRI
Margaret Delgado	National Institutes of Health, Undiagnosed Diseases Program, NHGRI
Marie Morimoto	National Institutes of Health, Undiagnosed Diseases Program, NHGRI
Marla Sabaii	National Institutes of Health, Undiagnosed Diseases Program, NHGRI
MayChristine V. Malicdan	National Institutes of Health, Undiagnosed Diseases Program, NHGRI
Neil Hanchard	National Institutes of Health, Undiagnosed Diseases Program, NHGRI
Orpa Jean-Marie	National Institutes of Health, Undiagnosed Diseases Program, NHGRI
Wendy Introne	National Institutes of Health, Undiagnosed Diseases Program, NHGRI
William A. Gahl	National Institutes of Health, Undiagnosed Diseases Program, NHGRI
Yan Huang	National Institutes of Health, Undiagnosed Diseases Program, NHGRI
Andrew Stergachis	Pacific Northwest
Danny Miller	Pacific Northwest
Elisabeth Rosenthal	Pacific Northwest
Elizabeth Blue	Pacific Northwest
Elsa Balton	Pacific Northwest
Emily Shelkowitz	Pacific Northwest
Eric Allenspach	Pacific Northwest
Fuki M. Hisama	Pacific Northwest
Gail P. Jarvik	Pacific Northwest
Ghayda Mirzaa	Pacific Northwest
Ian Glass	Pacific Northwest
Kathleen A. Leppig	Pacific Northwest
Katrina Dipple	Pacific Northwest
Mark Wener	Pacific Northwest
Martha Horike-Pyne	Pacific Northwest
Michael Bamshad	Pacific Northwest
Peter Byers	Pacific Northwest
Runjun Kumar	Pacific Northwest
Seth Perlman	Pacific Northwest
Sirisak Chanprasert	Pacific Northwest
Virginia Sybert	Pacific Northwest
Wendy Raskind	Pacific Northwest
Nitsuh K. Dargie	Pacific Northwest
Chun-Hung Chan	Sanford Health
Dr. Francisco Bustos velasq	Sanford Health
Isum Ward	Sanford Health
Jason Schend	Sanford Health
Jennifer Morgan	Sanford Health
Megan Bell	Sanford Health
Miranda Leitheiser	Sanford Health

Mohamad Saifeddine	Sanford Health
Paul Berger	Sanford Health
Rachel Li	Sanford Health
Taylor Beagle	Sanford Health
Alexander Miller	Stanford University
Beatriz Anguiano	Stanford University
Beth A. Martin	Stanford University
Brianna Tucker	Stanford University
Chloe M. Reuter	Stanford University
Devon Bonner	Stanford University
Elijah Kravets	Stanford University
Hector Rodrigo Mendez	Stanford University
Holly K. Tabor	Stanford University
Jacinda B. Sampson	Stanford University
Jason Hom	Stanford University
Jennefer N. Kohler	Stanford University
Jennifer Schymick	Stanford University
John E. Gorzynski	Stanford University
Jonathan A. Bernstein	Stanford University
Kevin S. Smith	Stanford University
Laura Keehan	Stanford University
Laurens Wiel	Stanford University
Matthew T. Wheeler	Stanford University
Meghan C. Halley	Stanford University
Mia Levanto	Stanford University
Page C. Goddard	Stanford University
Paul G. Fisher	Stanford University
Rachel A. Ungar	Stanford University
Raquel L. Alvarez	Stanford University
Sara Emami	Stanford University
Shruti Marwaha	Stanford University
Stephen B Montgomery	Stanford University
Suha Bachir	Stanford University
Tanner D Jensen	Stanford University
Taylor Maurer	Stanford University
Terra R. Coakley	Stanford University
Euan A. Ashley	Stanford University, DMCC
Ali Al-Beshri	University of Alabama
Anna Hurst	University of Alabama
Brandon M Wilk	University of Alabama
Bruce Korf	University of Alabama
Elizabeth A Worthey	University of Alabama
Kaitlin Callaway	University of Alabama
Martin Rodriguez	University of Alabama
Tammi Skelton	University of Alabama

Tarun KK Mamidi	University of Alabama
Andrew B. Crouse	University of Alabama, DMCC
Jordan Whitlock	University of Alabama, DMCC
Mariko Nakano-Okuno	University of Alabama, DMCC
Matthew Might	University of Alabama, DMCC
William E. Byrd	University of Alabama, DMCC
Albert R. La Spada	University of California Irvine, Children's Hospital of Orange County
Changrui Xiao	UC Irvine, Children's Hospital of Orange County
Elizabeth C. Chao	UC Irvine, Children's Hospital of Orange County
Eric Vilain	UC Irvine, Children's Hospital of Orange County
Jose Abdenur	UC Irvine, Children's Hospital of Orange County
Kathyrn Singh	UC Irvine, Children's Hospital of Orange County
Maija-Rikka Steenari	UC Irvine, Children's Hospital of Orange County
Rebekah Barrick	UC Irvine, Children's Hospital of Orange County
Sanaz Attaripour	UC Irvine, Children's Hospital of Orange County
Suzanne Sandmeyer	UC Irvine, Children's Hospital of Orange County
Tahseen Mozaffar	UC Irvine, Children's Hospital of Orange County
Alden Huang	University of California Los Angeles
Andres Vargas	University of California Los Angeles
Bianca E. Russell	University of California Los Angeles
Brent L. Fogel	University of California Los Angeles
Esteban C. Dell'Angelica	University of California Los Angeles
George Carvalho	University of California Los Angeles
Julian A. Martínez-Agosto	University of California Los Angeles
Layal F. Abi Farraj	University of California Los Angeles
Manish J. Butte	University of California Los Angeles
Martin G. Martin	University of California Los Angeles
Naghmeh Dorrani	University of California Los Angeles
Neil H. Parker	University of California Los Angeles
Rosario I. Corona	University of California Los Angeles
Stanley F. Nelson	University of California Los Angeles
Yigit Karasozen	University of California Los Angeles
Aaron Quinlan	University of Utah
Alistair Ward	University of Utah
Ashley Andrews	University of Utah
Corrine K. Welt	University of Utah
Dave Viskochil	University of Utah
Erin E. Baldwin	University of Utah
John Carey	University of Utah
Justin Alvey	University of Utah
Laura Pace	University of Utah
Lorenzo Botto	University of Utah
Nicola Longo	University of Utah
Paolo Moretti	University of Utah
Rebecca Overbury	University of Utah

Russell Butterfield	University of Utah
Steven Boyden	University of Utah
Thomas J. Nicholas	University of Utah
Matt Velinder	University of Utah
Gabor Marth	University of Utah, DMCC
Pinar Bayrak-Toydemir	University of Utah, ARUP laboratories
Rong Mao	University of Utah, ARUP laboratories
Monte Westerfield	University of Oregon, Model Organism Screening Center
Brian Corner	Vanderbilt University
John A. Phillips III	Vanderbilt University
Kimberly Ezell	Vanderbilt University
Lynette Rives	Vanderbilt University
Rizwan Hamid	Vanderbilt University
Serena Neumann	Vanderbilt University
Ashley McMinn	Vanderbilt University
Joy D. Cogan	Vanderbilt University
Thomas Cassini	Vanderbilt University
Alex Paul	Washington University in St Louis, Clinical Site
Dana Kiley	Washington University in St Louis, Clinical Site
Daniel Wegner	Washington University in St Louis, Clinical Site
Erin McRoy	Washington University in St Louis, Clinical Site
Jennifer Wambach	Washington University in St Louis, Clinical Site
Kathy Sisco	Washington University in St Louis, Clinical Site
Patricia Dickson	Washington University in St Louis, Clinical Site
F. Sessions Cole	Washington University in St Louis, DMCC
Dustin Baldridge	Washington University in St Louis, Model Organism Screening Center
Jimann Shin	Washington University in St Louis, Model Organism Screening Center
Lilianna Solnica-Krezel	Washington University in St Louis, Model Organism Screening Center
Stephen C. Pak	Washington University in St Louis, Model Organism Screening Center
Timothy Schedl	Washington University in St Louis, Model Organism Screening Center
Allen Bale	Yale University
Carol Oladele	Yale University
Caroline Hendry	Yale University
Emily Wang	Yale University
Hua Xu	Yale University
Hui Zhang	Yale University
Lauren Jeffries	Yale University
María José Ortuño Romero	Yale University
Mark Gerstein	Yale University
Michele Spencer-Manzon	Yale University
Monkol Lek	Yale University
Nada Derar	Yale University
Odelya Kaufman	Yale University
Shrikant Mane	Yale University
Teodoro Jerves Serrano	Yale University

Vasilis Vasiliou	Yale University
Winston Halstead	Yale University
Yong-Hui Jiang	Yale University
