# Supplementary material for: Dominant negative ATP5F1A variants disrupt oxidative phosphorylation causing neurological disorders
Source: EMBO Mol Med. 2025 Aug 26;17(10):2562–85. doi: 10.1038/s44321-025-00290-8 (PMC12514044; doi:10.1038/s44321-025-00290-8)

Fig. 3E - All images were rotated and/or flipped horizontally to present the animals in the same orientation (i.e., the anterior to the left, and the vulva pointing down). Images were also cropped (red box) to reduce empty space. Brightness and contrast were adjusted to show the internal organs and to highlight the age of the animals.


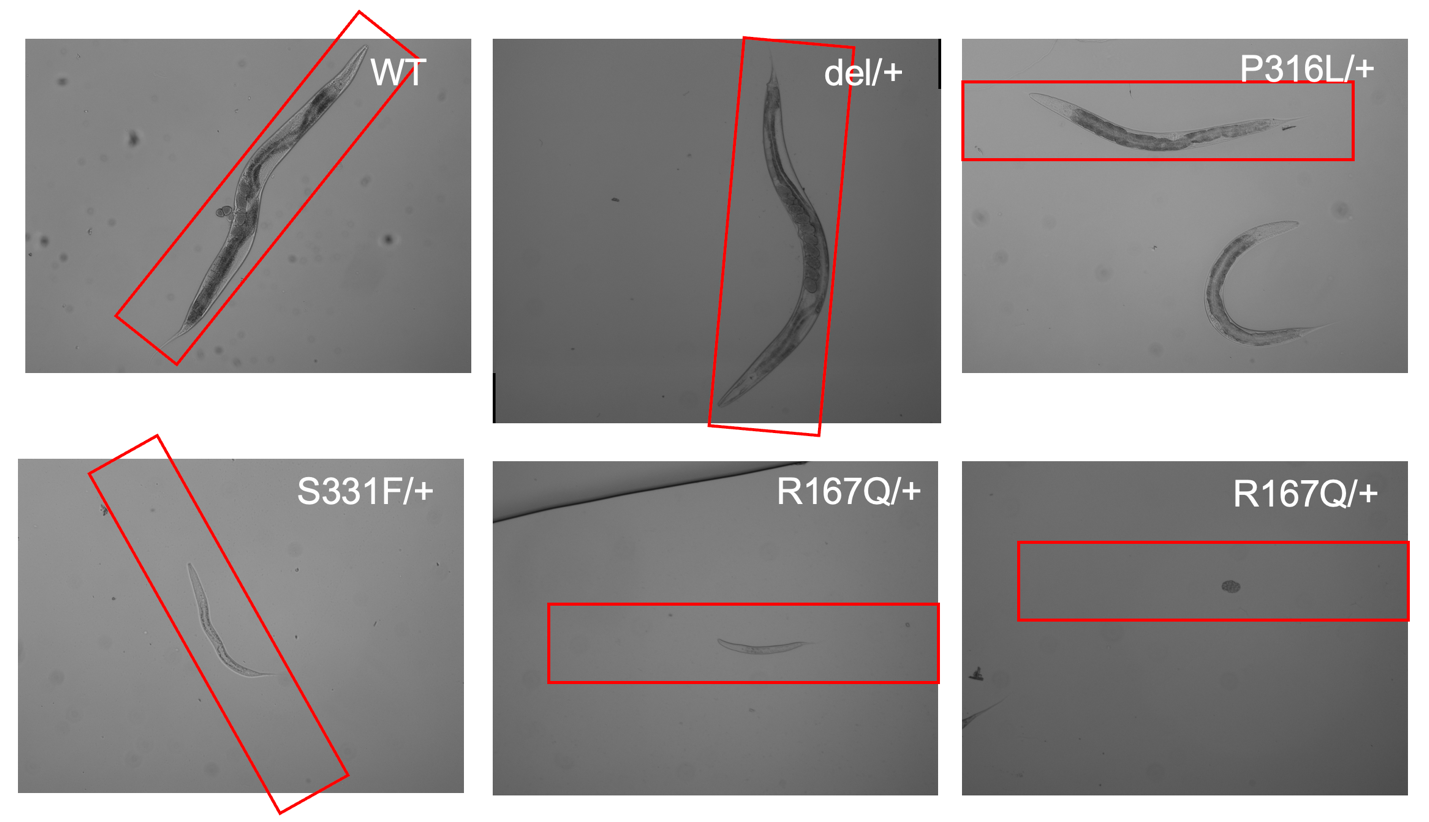

Supplement: Supplementary file 3 — Source data Fig. 3 [file 44321_2025_290_MOESM3_ESM.zip › Figure 3/Fig. 3E/Fig 3E_Readme.docx]
