# Supplementary material for: Dominant negative ATP5F1A variants disrupt oxidative phosphorylation causing neurological disorders
Source: EMBO Mol Med. 2025 Aug 26;17(10):2562–85. doi: 10.1038/s44321-025-00290-8 (PMC12514044; doi:10.1038/s44321-025-00290-8)
Supplement: Supplementary file 3 — Source data Fig. 3 [file 44321_2025_290_MOESM3_ESM.zip › Figure 3/Fig. 3A/Fig 3A_Multiple Sequence Alignment_Marvel.pptx]

## Slide 1
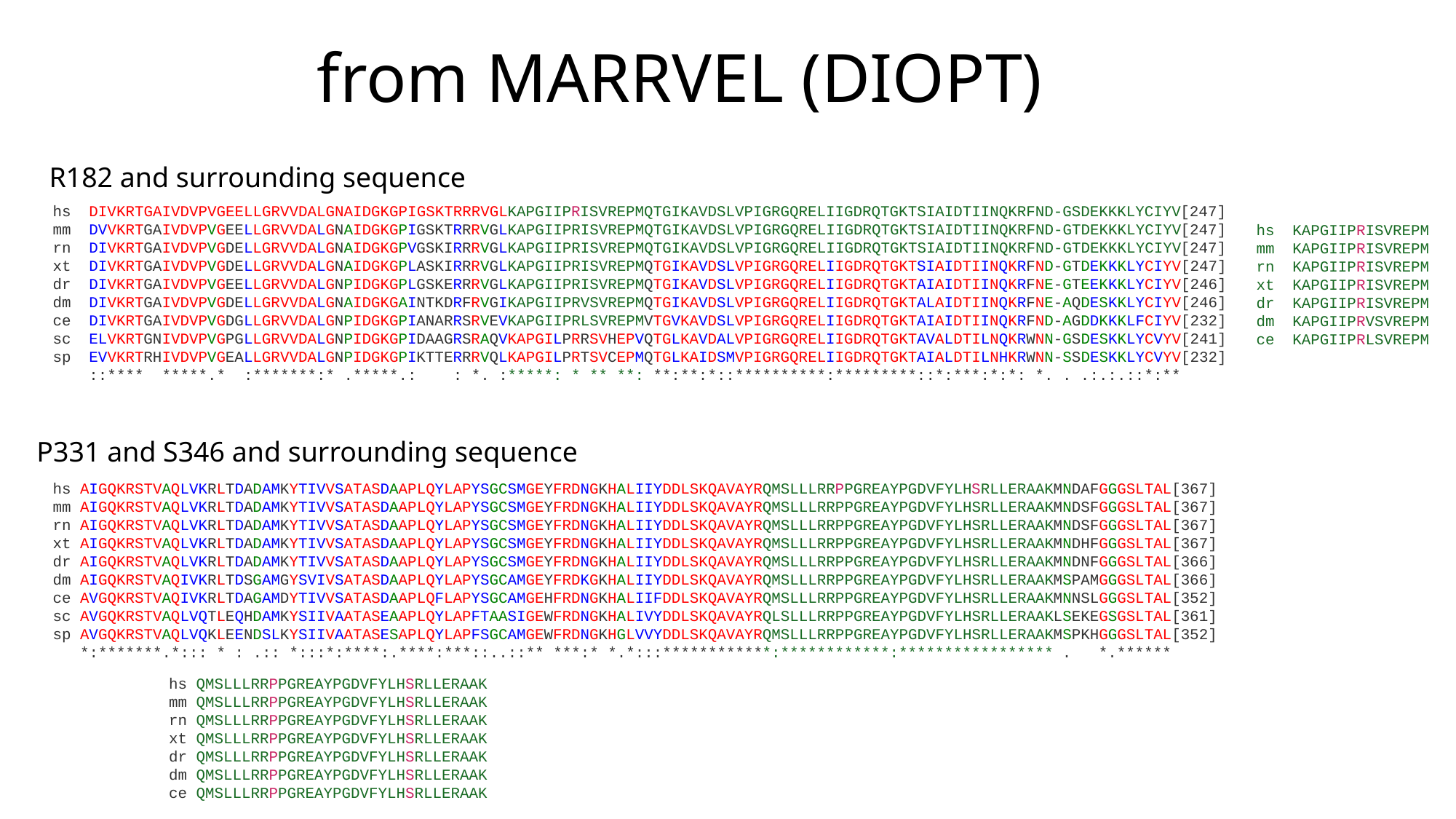

# from MARRVEL (DIOPT)
R182 and surrounding sequence
hs DIVKRTGAIVDVPVGEELLGRVVDALGNAIDGKGPIGSKTRRRVGLKAPGIIPRISVREPMQTGIKAVDSLVPIGRGQRELIIGDRQTGKTSIAIDTIINQKRFND-GSDEKKKLYCIYV[247]
mm DVVKRTGAIVDVPVGEELLGRVVDALGNAIDGKGPIGSKTRRRVGLKAPGIIPRISVREPMQTGIKAVDSLVPIGRGQRELIIGDRQTGKTSIAIDTIINQKRFND-GTDEKKKLYCIYV[247]
rn DIVKRTGAIVDVPVGDELLGRVVDALGNAIDGKGPVGSKIRRRVGLKAPGIIPRISVREPMQTGIKAVDSLVPIGRGQRELIIGDRQTGKTSIAIDTIINQKRFND-GTDEKKKLYCIYV[247]
xt DIVKRTGAIVDVPVGDELLGRVVDALGNAIDGKGPLASKIRRRVGLKAPGIIPRISVREPMQTGIKAVDSLVPIGRGQRELIIGDRQTGKTSIAIDTIINQKRFND-GTDEKKKLYCIYV[247]
dr DIVKRTGAIVDVPVGEELLGRVVDALGNPIDGKGPLGSKERRRVGLKAPGIIPRISVREPMQTGIKAVDSLVPIGRGQRELIIGDRQTGKTAIAIDTIINQKRFNE-GTEEKKKLYCIYV[246]
dm DIVKRTGAIVDVPVGDELLGRVVDALGNAIDGKGAINTKDRFRVGIKAPGIIPRVSVREPMQTGIKAVDSLVPIGRGQRELIIGDRQTGKTALAIDTIINQKRFNE-AQDESKKLYCIYV[246]
ce DIVKRTGAIVDVPVGDGLLGRVVDALGNPIDGKGPIANARRSRVEVKAPGIIPRLSVREPMVTGVKAVDSLVPIGRGQRELIIGDRQTGKTAIAIDTIINQKRFND-AGDDKKKLFCIYV[232]
sc ELVKRTGNIVDVPVGPGLLGRVVDALGNPIDGKGPIDAAGRSRAQVKAPGILPRRSVHEPVQTGLKAVDALVPIGRGQRELIIGDRQTGKTAVALDTILNQKRWNN-GSDESKKLYCVYV[241]
sp EVVKRTRHIVDVPVGEALLGRVVDALGNPIDGKGPIKTTERRRVQLKAPGILPRTSVCEPMQTGLKAIDSMVPIGRGQRELIIGDRQTGKTAIALDTILNHKRWNN-SSDESKKLYCVYV[232]
 ::****  *****.*  :*******:* .*****.:    : *. :*****: * ** **: **:**:*::**********:*********::*:***:*:*: *. . .:.:.::*:**
hs KAPGIIPRISVREPM
mm KAPGIIPRISVREPM
rn KAPGIIPRISVREPM
xt KAPGIIPRISVREPM
dr KAPGIIPRISVREPM
dm KAPGIIPRVSVREPM
ce KAPGIIPRLSVREPM
P331 and S346 and surrounding sequence
hs AIGQKRSTVAQLVKRLTDADAMKYTIVVSATASDAAPLQYLAPYSGCSMGEYFRDNGKHALIIYDDLSKQAVAYRQMSLLLRRPPGREAYPGDVFYLHSRLLERAAKMNDAFGGGSLTAL[367]
mm AIGQKRSTVAQLVKRLTDADAMKYTIVVSATASDAAPLQYLAPYSGCSMGEYFRDNGKHALIIYDDLSKQAVAYRQMSLLLRRPPGREAYPGDVFYLHSRLLERAAKMNDSFGGGSLTAL[367]
rn AIGQKRSTVAQLVKRLTDADAMKYTIVVSATASDAAPLQYLAPYSGCSMGEYFRDNGKHALIIYDDLSKQAVAYRQMSLLLRRPPGREAYPGDVFYLHSRLLERAAKMNDSFGGGSLTAL[367]
xt AIGQKRSTVAQLVKRLTDADAMKYTIVVSATASDAAPLQYLAPYSGCSMGEYFRDNGKHALIIYDDLSKQAVAYRQMSLLLRRPPGREAYPGDVFYLHSRLLERAAKMNDHFGGGSLTAL[367]
dr AIGQKRSTVAQLVKRLTDADAMKYTIVVSATASDAAPLQYLAPYSGCSMGEYFRDNGKHALIIYDDLSKQAVAYRQMSLLLRRPPGREAYPGDVFYLHSRLLERAAKMNDNFGGGSLTAL[366]
dm AIGQKRSTVAQIVKRLTDSGAMGYSVIVSATASDAAPLQYLAPYSGCAMGEYFRDKGKHALIIYDDLSKQAVAYRQMSLLLRRPPGREAYPGDVFYLHSRLLERAAKMSPAMGGGSLTAL[366]
ce AVGQKRSTVAQIVKRLTDAGAMDYTIVVSATASDAAPLQFLAPYSGCAMGEHFRDNGKHALIIFDDLSKQAVAYRQMSLLLRRPPGREAYPGDVFYLHSRLLERAAKMNNSLGGGSLTAL[352]
sc AVGQKRSTVAQLVQTLEQHDAMKYSIIVAATASEAAPLQYLAPFTAASIGEWFRDNGKHALIVYDDLSKQAVAYRQLSLLLRRPPGREAYPGDVFYLHSRLLERAAKLSEKEGSGSLTAL[361]
sp AVGQKRSTVAQLVQKLEENDSLKYSIIVAATASESAPLQYLAPFSGCAMGEWFRDNGKHGLVVYDDLSKQAVAYRQMSLLLRRPPGREAYPGDVFYLHSRLLERAAKMSPKHGGGSLTAL[352]
 *:*******.*::: * : .:: *:::*:****:.****:***::..::** ***:* *.*:::************:************:***************** .   *.******
hs QMSLLLRRPPGREAYPGDVFYLHSRLLERAAK
mm QMSLLLRRPPGREAYPGDVFYLHSRLLERAAK
rn QMSLLLRRPPGREAYPGDVFYLHSRLLERAAK
xt QMSLLLRRPPGREAYPGDVFYLHSRLLERAAK
dr QMSLLLRRPPGREAYPGDVFYLHSRLLERAAK
dm QMSLLLRRPPGREAYPGDVFYLHSRLLERAAK
ce QMSLLLRRPPGREAYPGDVFYLHSRLLERAAK
