# Supplementary material for: Dominant negative ATP5F1A variants disrupt oxidative phosphorylation causing neurological disorders
Source: EMBO Mol Med. 2025 Aug 26;17(10):2562–85. doi: 10.1038/s44321-025-00290-8 (PMC12514044; doi:10.1038/s44321-025-00290-8)
Supplement: Supplementary file 3 — Source data Fig. 3 [file 44321_2025_290_MOESM3_ESM.zip › Figure 3/Fig. 3A/Fig 3A_Multiple Sequence Alignment_DIOPT.pdf]

```
CLUSTAL format alignment by MAFFT FFT-NS-2 (v7.305b)


hs|498|243893|9 ------------------------------MLSVRVAAAVVRALPR--------------
dr|553755|50776 ------------------------------MLSVRVAAALARTLPR--------------
mm|11946|385122 ------------------------------MLSVRVAAAVARALPR--------------
rn|65262|460366 ------------------------------MLSVRIAAAVARALPR--------------
dm|37617|33961| ----------------------------MSIFSARLASSVARNLPK--------------
sc|852177|9707| ---------------------------------MLARTAAIRSLSR--------------
ce|173134|49849 ------------------------------MLSKRIVTALNTAV----------------
sp|2541526|7611 ----------------------------------MLRQAGTRLLKV--------------
at|815374|10713 MITRLFAQLVSLSIVTYWNDAIVATNFSWLFITFFVMTFTFRTFSRYFKKPIIWTFYFFL
ag|1274812|7649 ----------------------------MSMISVRLAASVARSLPR--------------
ec|948242|1068| ------------------------------------------------------------
xt|594998|13905 ------------------------------MLSVRVATALARTLPR--------------


hs|498|243893|9 --------------------------------------------------RAGLVS-RNA
dr|553755|50776 --------------------------------------------------RAGFVS-KN-
mm|11946|385122 --------------------------------------------------RAGLVS-KNA
rn|65262|460366 --------------------------------------------------RAGLVS-KNA
dm|37617|33961| --------------------------------------------------AANQVACKAA
sc|852177|9707| --------------------------------------------------TLINST-KAA
ce|173134|49849 ---------------------------------------------------------KVQ
sp|2541526|7611 --------------------------------------------------PVC-----GL
at|815374|10713 CLIAFLLLWAARIHINILFSFAFGDVYSFFMAGVFLFYGFGELLPIGSDSDVGEAS-WVV
ag|1274812|7649 --------------------------------------------------TATQVA-KIA
ec|948242|1068| ------------------------------------------------------------
xt|594998|13905 --------------------------------------------------QAGLVS-KKA


hs|498|243893|9 LGSSF-------------------------------------------------------
dr|553755|50776 VAAAC-------------------------------------------------------
mm|11946|385122 LGSSF-------------------------------------------------------
rn|65262|460366 LGSSF-------------------------------------------------------
dm|37617|33961| YPAAS-------------------------------------------------------
sc|852177|9707| RPAAAA------------------------------------------------------
ce|173134|49849 NAGIA-------------------------------------------------------
sp|2541526|7611 RPS---------------------------------------------------------
at|815374|10713 NPATGASGSGGNGWTESAANDPAREVSLAPFPLQLTHPVPFPAEPGSPDPVSPPPPIASF
ag|1274812|7649 VPAVS-------------------------------------------------------
ec|948242|1068| ------------------------------------------------------------
xt|594998|13905 LGAAF-------------------------------------------------------


hs|498|243893|9 ----IAARNFHASNTHL-------------------------------------------
dr|553755|50776 ----VGAKNLHTARPWL-------------------------------------------
mm|11946|385122 ----VGARNLHASNTRL-------------------------------------------
rn|65262|460366 ----VGTRNLHASNTRL-------------------------------------------
dm|37617|33961| ----LAARKLHVASTQ--------------------------------------------
sc|852177|9707| ---LASTRRLAST-----------------------------------------------
ce|173134|49849 ----TTARGMAGA-----------------------------------------------
sp|2541526|7611 ----ITLKRGYAE-----------------------------------------------
at|815374|10713 YSRIERAESLHAGNIELAEDLQRIQEMERNLENERSPYRGRELAARIDWEVRELEGKVAR
ag|1274812|7649 ----VAARNFHVSTAH--------------------------------------------
ec|948242|1068| ------------------------------------------------------------
xt|594998|13905 ----VATRNIHASGAWL-------------------------------------------


hs|498|243893|9 ---------------------------------QKTGTAEMSSILEERILGADTSVDLEE
dr|553755|50776 ---------------------------------QKTGTAEVSSILEEKILGADTGAELEE
mm|11946|385122 ---------------------------------QKTGTAEMSSILEERILGADTSVDLEE
rn|65262|460366 ---------------------------------QKTGTAEMSSILEERILGADTSVDLEE
dm|37617|33961| ------------------------------------RSAEISNILEERILGVAPKADLEE
```

```
sc|852177|9707| ----------------------------------KAQPTEVSSILEERIKGVSDEANLNE
ce|173134|49849 ------------------------------------SGSEVSKILEERILGTETGINLEE
sp|2541526|7611 ----------------------------------KAAPTEVPSILEERIRGAYNQAQMME
at|815374|10713 NRAWDMVRDAQLDIWRQGLDQELVRQQENESRLEERRAAELTNLFESRIRNFYANFQVDE
ag|1274812|7649 ------------------------------------RGAEISAILEERILGAAPKADLEE
ec|948242|1068| ---------------------------------MQLNSTEISELIKQRIAQFNVVSEAHN
xt|594998|13905 ---------------------------------QKSGTAEVSSILEERILGADTSADLEE
                                                      :*:. :::.:*       :  :

hs|498|243893|9 TGRVLSIGDGIARVHGLRNVQAEEMVEFSSGLKGMSLNLEPDNVGVVVFGNDKLIKEGDI
dr|553755|50776 TGRVLSIGDGIARVYGLRNVQAEEMVEFSSGLKGMSLNLEPDNVGVVVFGNDKLIKEGDI
mm|11946|385122 TGRVLSIGDGIARVHGLRNVQAEEMVEFSSGLKGMSLNLEPDNVGVVVFGNDKLIKEGDV
rn|65262|460366 TGRVLSIGDGIARVHGLRNVQAEEMVEFSSGLKGMSLNLEPDNVGVVVFGNDKLIKEGDI
dm|37617|33961| TGRVLSIGDGIARVYGLNNIQADEMVEFSSGLKGMALNLEPDNVGVVVFGNDKLIKQGDI
sc|852177|9707| TGRVLAVGDGIARVFGLNNIQAEELVEFSSGVKGMALNLEPGQVGIVLFGSDRLVKEGEL
ce|173134|49849 TGKVLSIGDGIARVYGLKNIQAEEMVEFDSGIKGMAMNLDVDNVGVVVFGNDKVIREGDI
sp|2541526|7611 SGRVLSIGDGIARISGLSNVQAEELVEFSSGIKGMALNLEADTVGCVLFGNDRLVREGEV
at|815374|10713 IGRVVSVGDGIAQVYGLNEIQAGEMVLFANGVKGMALNLENENVGIVVFGGDTAIKEGDL
ag|1274812|7649 TGRVLSIGDGIARVYGLKNIQADEMVEFSSGLKGMALNLEPDNVGVVVFGNDKLIKEGDI
ec|948242|1068| EGTIVSVSDGVIRIHGLADCMQGEMISLPGNRYAIALNLERDSVGAVVMGPYADLAEGMK
xt|594998|13905 TGRVLSIGDGIARVYGLRNVQAEEMVEFSSGLKGMSLNLEPDNVGVVVFGNDKLIKEGDI
                 * ::::.**: :: ** :    *:: : ..  .:::**:   ** *::*    : :*

hs|498|243893|9 VKRTGAIVDVPVGEELLGRVVDALGNAIDGKGPIGSKTRRRVGLKAPGIIPRISVREPMQ
dr|553755|50776 VKRTGAIVDVPVGEELLGRVVDALGNPIDGKGPLGSKERRRVGLKAPGIIPRISVREPMQ
mm|11946|385122 VKRTGAIVDVPVGEELLGRVVDALGNAIDGKGPIGSKTRRRVGLKAPGIIPRISVREPMQ
rn|65262|460366 VKRTGAIVDVPVGDELLGRVVDALGNAIDGKGPVGSKIRRRVGLKAPGIIPRISVREPMQ
dm|37617|33961| VKRTGAIVDVPVGDELLGRVVDALGNAIDGKGAINTKDRFRVGIKAPGIIPRVSVREPMQ
sc|852177|9707| VKRTGNIVDVPVGPGLLGRVVDALGNPIDGKGPIDAAGRSRAQVKAPGILPRRSVHEPVQ
ce|173134|49849 VKRTGAIVDVPVGDGLLGRVVDALGNPIDGKGPIANARRSRVEVKAPGIIPRLSVREPMV
sp|2541526|7611 VKRTRHIVDVPVGEALLGRVVDALGNPIDGKGPIKTTERRRVQLKAPGILPRTSVCEPMQ
at|815374|10713 VKRTGSIVDVPAGKAMLGRVVDAMGVPIDGKGALSDHEQRRVEVKAPGILERKSVHEPMQ
ag|1274812|7649 VKRTGAIVDVPVGDEILGRVVDALGNAIDGKGEIKTKQRFRVGIKAPGIIPRVSVREPMQ
ec|948242|1068| VKCTGRILEVPVGRGLLGRVVNTLGAPIDGKGPLDHDGFSAVEAIAPGVIERQSVDQPVQ
xt|594998|13905 VKRTGAIVDVPVGDELLGRVVDALGNAIDGKGPLASKIRRRVGLKAPGIIPRISVREPMQ
                ** *  *::**.*  :*****:::* .***** :       .   ***:: * ** :*:

hs|498|243893|9 TGIKAVDSLVPIGRGQRELIIGDRQTGKTSIAIDTIINQKRFND-GSDEKKKLYCIYVAI
dr|553755|50776 TGIKAVDSLVPIGRGQRELIIGDRQTGKTAIAIDTIINQKRFNE-GTEEKKKLYCIYVAI
mm|11946|385122 TGIKAVDSLVPIGRGQRELIIGDRQTGKTSIAIDTIINQKRFND-GTDEKKKLYCIYVAI
rn|65262|460366 TGIKAVDSLVPIGRGQRELIIGDRQTGKTSIAIDTIINQKRFND-GTDEKKKLYCIYVAI
dm|37617|33961| TGIKAVDSLVPIGRGQRELIIGDRQTGKTALAIDTIINQKRFNE-AQDESKKLYCIYVAI
sc|852177|9707| TGLKAVDALVPIGRGQRELIIGDRQTGKTAVALDTILNQKRWNN-GSDESKKLYCVYVAV
ce|173134|49849 TGVKAVDSLVPIGRGQRELIIGDRQTGKTAIAIDTIINQKRFND-AGDDKKKLFCIYVAV
sp|2541526|7611 TGLKAIDSMVPIGRGQRELIIGDRQTGKTAIALDTILNHKRWNN-SSDESKKLYCVYVAV
at|815374|10713 TGLKAVDSLVPIGRGQRELLIGDRQTGKTTIAIDTILNQKQINSRATSESETMYCVYVAI
ag|1274812|7649 TGIKAVDSLVPIGRGQRELIIGDRQTGKTALAIDTIINQKRFND-GQDESKKLYCIYVAI
ec|948242|1068| TGYKAVDSMIPIGRGQRELIIGDRQTGKTALAIDAIINQR---------DSGIKCIYVAI
xt|594998|13905 TGIKAVDSLVPIGRGQRELIIGDRQTGKTSIAIDTIINQKRFND-GTDEKKKLYCIYVAI
                ** **:*:::*********:*********::*:*:*:*::         .. : *:***:

hs|498|243893|9 GQKRSTVAQLVKRLTDADAMKYTIVVSATASDAAPLQYLAPYSGCSMGEYFRDNGKHALI
dr|553755|50776 GQKRSTVAQLVKRLTDADAMKYTIVVSATASDAAPLQYLAPYSGCSMGEYFRDNGKHALI
mm|11946|385122 GQKRSTVAQLVKRLTDADAMKYTIVVSATASDAAPLQYLAPYSGCSMGEYFRDNGKHALI
rn|65262|460366 GQKRSTVAQLVKRLTDADAMKYTIVVSATASDAAPLQYLAPYSGCSMGEYFRDNGKHALI
dm|37617|33961| GQKRSTVAQIVKRLTDSGAMGYSVIVSATASDAAPLQYLAPYSGCAMGEYFRDKGKHALI
sc|852177|9707| GQKRSTVAQLVQTLEQHDAMKYSIIVAATASEAAPLQYLAPFTAASIGEWFRDNGKHALI
ce|173134|49849 GQKRSTVAQIVKRLTDAGAMDYTIVVSATASDAAPLQFLAPYSGCAMGEHFRDNGKHALI
sp|2541526|7611 GQKRSTVAQLVQKLEENDSLKYSIIVAATASESAPLQYLAPFSGCAMGEWFRDNGKHGLV
at|815374|10713 GQKRSTVGQLIQTLEEANALEYSILVAATASDPAPLQFLAPYSGCAMGEYFRDNGMHALI
ag|1274812|7649 GQKRSTVAQIVKRLTDSGAMNYTIIVSATASDAAPLQYLAPYSGCAMGEYFRDNGKHALI
ec|948242|1068| GQKASTISNVVRKLEEHGALANTIVVVATASESAALQYLAPYAGCAMGEYFRDRGEDALI
xt|594998|13905 GQKRSTVAQLVKRLTDADAMKYTIVVSATASDAAPLQYLAPYSGCSMGEYFRDNGKHALI
                *** **:.:::: * : .::  :::* ****:.*.**:***::..::** ***.* ..*:
```

```
hs|498|243893|9 IYDDLSKQAVAYRQMSLLLRRPPGREAYPGDVFYLHSRLLERAAKMNDAF----------
dr|553755|50776 IYDDLSKQAVAYRQMSLLLRRPPGREAYPGDVFYLHSRLLERAAKMNDNF----------
mm|11946|385122 IYDDLSKQAVAYRQMSLLLRRPPGREAYPGDVFYLHSRLLERAAKMNDSF----------
rn|65262|460366 IYDDLSKQAVAYRQMSLLLRRPPGREAYPGDVFYLHSRLLERAAKMNDSF----------
dm|37617|33961| IYDDLSKQAVAYRQMSLLLRRPPGREAYPGDVFYLHSRLLERAAKMSPAM----------
sc|852177|9707| VYDDLSKQAVAYRQLSLLLRRPPGREAYPGDVFYLHSRLLERAAKLSEKE----------
ce|173134|49849 IFDDLSKQAVAYRQMSLLLRRPPGREAYPGDVFYLHSRLLERAAKMNNSL----------
sp|2541526|7611 VYDDLSKQAVAYRQMSLLLRRPPGREAYPGDVFYLHSRLLERAAKMSPKH----------
at|815374|10713 IYDDLSKQAVAYRQMSLLLRRPPGREAFPGDVFYLHSRLLERAAKRSDQT----------
ag|1274812|7649 IYDDLSKQAVAYRQMSLLLRRPPGREAYPGDVFYLHSRLLERAAKMSPTL----------
ec|948242|1068| IYDDLSKQAVAYRQISLLLRRPPGREAFPGDVFYLHSRLLERAARVNAEYVEAFTKGEVK
xt|594998|13905 IYDDLSKQAVAYRQMSLLLRRPPGREAYPGDVFYLHSRLLERAAKMNDHF----------
                ::************:************:****************: .

hs|498|243893|9 -GGGSLTALPVIETQAGDVSAYIPTNVISITDGQIFLETELFYKGIRPAINVGLSVSRVG
dr|553755|50776 -GGGSLTALPVIETQAGDVSAYIPTNVISITDGQIFLETELFYKGIRPAINVGLSVSRVG
mm|11946|385122 -GGGSLTALPVIETQAGDVSAYIPTNVISITDGQIFLETELFYKGIRPAINVGLSVSRVG
rn|65262|460366 -GGGSLTALPVIETQAGDVSAYIPTNVISITDGQIFLETELFYKGIRPAINVGLSVSRVG
dm|37617|33961| -GGGSLTALPVIETQAGDVSAYIPTNVISITDGQIFLETELFYKGIRPAINVGLSVSRVG
sc|852177|9707| -GSGSLTALPVIETQGGDVSAYIPTNVISITDGQIFLEAELFYKGIRPAINVGLSVSRVG
ce|173134|49849 -GGGSLTALPVIETQAGDVSAYIPTNVISITDGQIFLETELFYKGVRPAINVGLSVSRVG
sp|2541526|7611 -GGGSLTALPVIETQGGDVSAYIPTNVISITDGQIFLESELFFKGIRPAINVGLSVSRVG
at|815374|10713 -GAGSLTALPVIETQAGDVSAYIPTNVISITDGQICLETELFYRGIRPAINVGLSVSRVG
ag|1274812|7649 -GGGSLTALPVIETQAGDVSAYIPTNVISITDGQIFLETELFYKGIRPAINVGLSVSRVG
ec|948242|1068| GKTGSLTALPIIETQAGDVSAFVPTNVISITDGQIFLETNLFNAGIRPAVNPGISVSRVG
xt|594998|13905 -GGGSLTALPVIETQAGDVSAYIPTNVISITDGQIFLETELFYKGIRPAINVGLSVSRVG
                   *******:****.*****::************ **::**  *:***:* *:******

hs|498|243893|9 SAAQTRAMKQVAGTMKLELAQYREVAAFAQFGSDLDAATQQLLSRGVRLTELLKQGQYS-
dr|553755|50776 SAAQTRAMKQVAGTMKLELAQYREVAAFAQFGSDLDAATQQLLNRGVRLTELLKQGQYS-
mm|11946|385122 SAAQTRAMKQVAGTMKLELAQYREVAAFAQFGSDLDAATQQLLSRGVRLTELLKQGQYS-
rn|65262|460366 SAAQTRAMKQVAGTMKLELAQYREVAAFAQFGSDLDAATQQLLSRGVRLTELLKQGQYS-
dm|37617|33961| SAAQTKAMKQVAGSMKLELAQYREVAAFAQFGSDLDAATQQLLNRGVRLTELLKQGQYV-
sc|852177|9707| SAAQVKALKQVAGSLKLFLAQYREVAAFAQFGSDLDASTKQTLVRGERLTQLLKQNQYS-
ce|173134|49849 SAAQTKAMKQVAGSMKLELAQYREVAAFAQFGSDLDASTQQLLNRGVRLTELLKQGQYV-
sp|2541526|7611 SAAQVKAMKQVAGQIKLFLAQYREVASFAQFGSDLDAGTRATLDRGLRLTELLKQPQYS-
at|815374|10713 SAAQLKAMKQVCGSSKLELAQYREVAAFAQFGSDLDAATQALLNRGARLTEVPKQPQYA-
ag|1274812|7649 SAAQTKAMKQVAGSMKLELAQYREVAAFAQFGSDLDAATQQLLNRGVRLTELLKQGQYV-
ec|948242|1068| GAAQTKIMKKLSGGIRTALAQYRELAAFSQFASDLDDATRKQLDHGQKVTELLKQKQYA-
xt|594998|13905 SAAQTRAMKQVAGTMKLELAQYREVAAFAQFGSDLDAATQQLLNRGVRLTELLKQGQYGK
                .*** : :*::.*  :  ******:*:*:**.**** .*:  * :* ::*:: ** **

hs|498|243893|9 ---------------------------------PMAIEEQVAVIYAGVRGYLDKLEPSKI
dr|553755|50776 ---------------------------------PMAIEEQVAVIYAGVRGHLDKMEPSKI
mm|11946|385122 ---------------------------------PMAIEEQVAVIYAGVRGYLDKLEPSKI
rn|65262|460366 ---------------------------------PMAIEEQVAVIYAGVRGYLDKLEPSKI
dm|37617|33961| ---------------------------------PMAIEDQVAVIYCGVRGHLDKMDPAKI
sc|852177|9707| ---------------------------------PLATEEQVPLIYAGVNGHLDGIELSRI
ce|173134|49849 ---------------------------------PMGIEEQVGVIYAGVKGYLDKVDPSAI
sp|2541526|7611 ---------------------------------PLAVEEQVPLIYCGVKGYLDKIPVDRV
at|815374|10713 ---------------------------------PLPIEKQILVIYAAVNGFCDRMPLDRI
ag|1274812|7649 ---------------------------------PMAIEEQVAVIYCGVRGYLDKMDPSKI
ec|948242|1068| ---------------------------------PMSVAQQSLVLFAAERGYLADVELSKI
xt|594998|13905 YLLGPQRLSRLPICVSACSSRAISFHQCAFVAVPMAIEEQVTVIYAGVRGHLDKMEPSKI
                                                 *:   .*  :::.. .*.   :    :

hs|498|243893|9 TKFENAFLSHVVSQHQALLGTIRYECNCWHLFLKLCLRYEVKIIFKSVVRLVIKTFSRSE
dr|553755|50776 TKFEKAFLQHVISQHQDLLAAIRSDG-------KISEASDAKL--KEIVLNFLSSFE---
mm|11946|385122 TKFENAFLSHVISQHQSLLGNIRSDG-------KISEQSDAKL--KEIVTNFLAGFEP--
rn|65262|460366 TKFESAFLSHVVSQHQSLLGNIRSDG-------KISEQSDAKL--KEIVTNFLAGFEP--
dm|37617|33961| TKFEKEFLQHIKTSEQALLDTIAKDG-------AISEASDAKL--KDIVAKFMSTFQG--
sc|852177|9707| GEFESSFLSYLKSNHNELLTEIREKG-------ELSKELLASL--KSATESFVATF----
ce|173134|49849 TKFEKEFLAHLRSSQQALLKTIREEG-------QISPQTDAQL--KDVVVNFLATFKP--
sp|2541526|7611 VEFEHKFIPYLRSSGAEIMEAIRKEG-------VLSKTTEDSL--KAVIKEFLSSF----
at|815374|10713 SQYEKAIPNSVKP---ELLQALKGGLTN-----ERKMEPDAFL--KERALALI-------
```

```
ag|1274812|7649 TKFEREFLAHVKTNEKALLQQIASEG-------KISDDADAKL--KSVVTSFMSTFSA--
ec|948242|1068| GSFEAALLAYVDRDHAPLMQEINQTG-------GYNDEIEGKL--KGILDSFKATQSW--
xt|594998|13905 TKFENAFLAHVKSQHQELLATIRADG-------KISEQADAKL--KEIVLSFLSTFEA--
                 .:*  :   :      ::  :                    :  *     .

hs|498|243893|9 LTTCFYFQIGLMERSQNNQMQS
dr|553755|50776 ----------------------
mm|11946|385122 ----------------------
rn|65262|460366 ----------------------
dm|37617|33961| ----------------------
sc|852177|9707| ----------------------
ce|173134|49849 ----------------------
sp|2541526|7611 ----------------------
at|815374|10713 ----------------------
ag|1274812|7649 ----------------------
ec|948242|1068| ----------------------
xt|594998|13905 ----------------------
```
