# Supplementary figures and images for: Dominant negative ATP5F1A variants disrupt oxidative phosphorylation causing neurological disorders
Source: EMBO Mol Med. 2025 Aug 26;17(10):2562–85. doi: 10.1038/s44321-025-00290-8 (PMC12514044; doi:10.1038/s44321-025-00290-8)

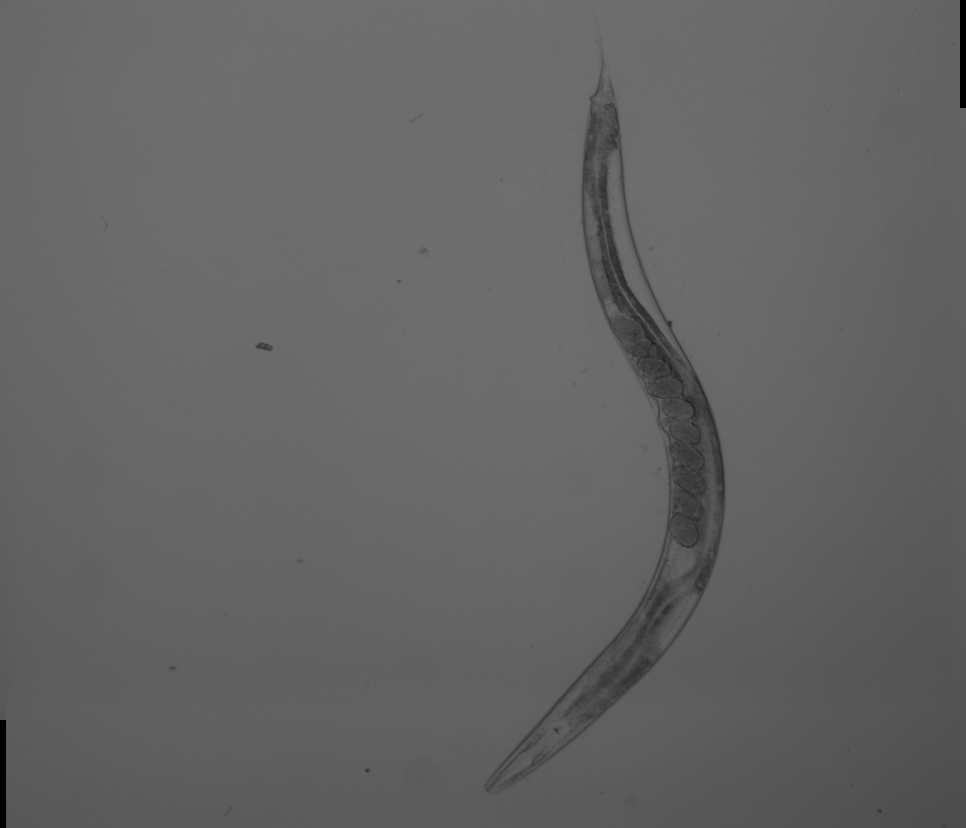

Supplement: Supplementary file 3 — Source data Fig. 3 [file 44321_2025_290_MOESM3_ESM.zip › Figure 3/Fig. 3E/Fig 3E_2_del het.tif]

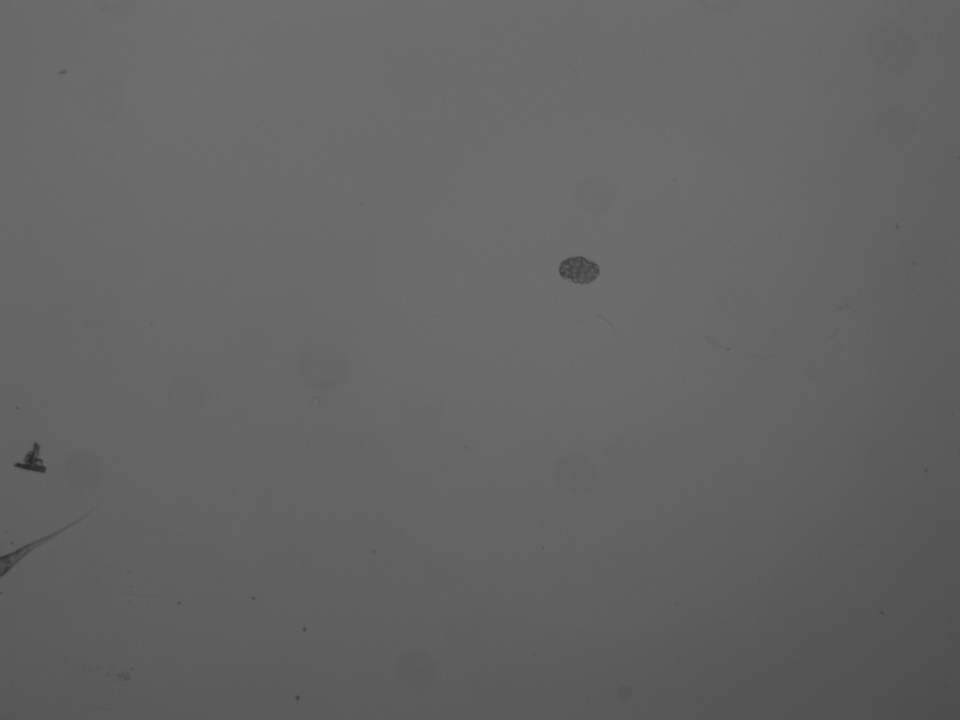

Supplement: Supplementary file 3 — Source data Fig. 3 [file 44321_2025_290_MOESM3_ESM.zip › Figure 3/Fig. 3E/Fig 3E_6_R167Q dead emb.tif]

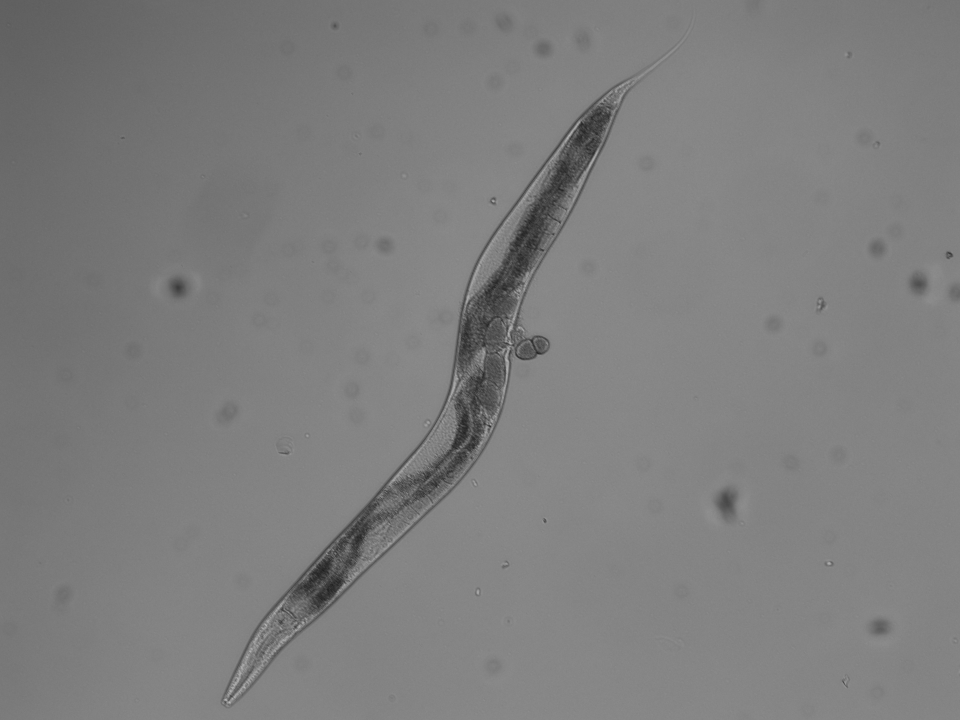

Supplement: Supplementary file 3 — Source data Fig. 3 [file 44321_2025_290_MOESM3_ESM.zip › Figure 3/Fig. 3E/Fig 3E_1_WT.tif]

## Slide 1
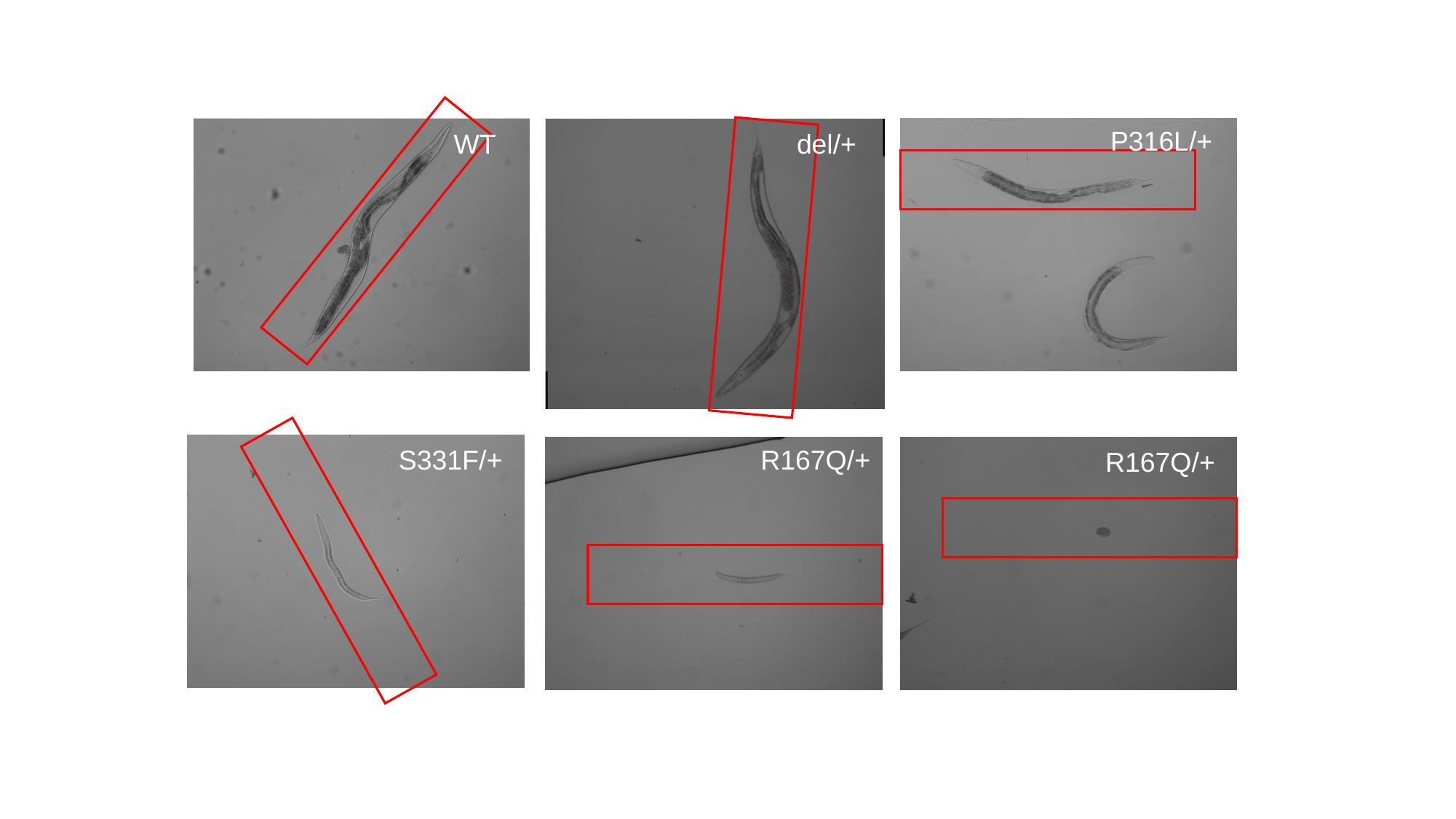

P316L/+
del/+
WT
S331F/+
R167Q/+
R167Q/+

Supplement: Supplementary file 3 — Source data Fig. 3 [file 44321_2025_290_MOESM3_ESM.zip › Figure 3/Fig. 3E/Fig 3E_Image processing and cropping.pptx]

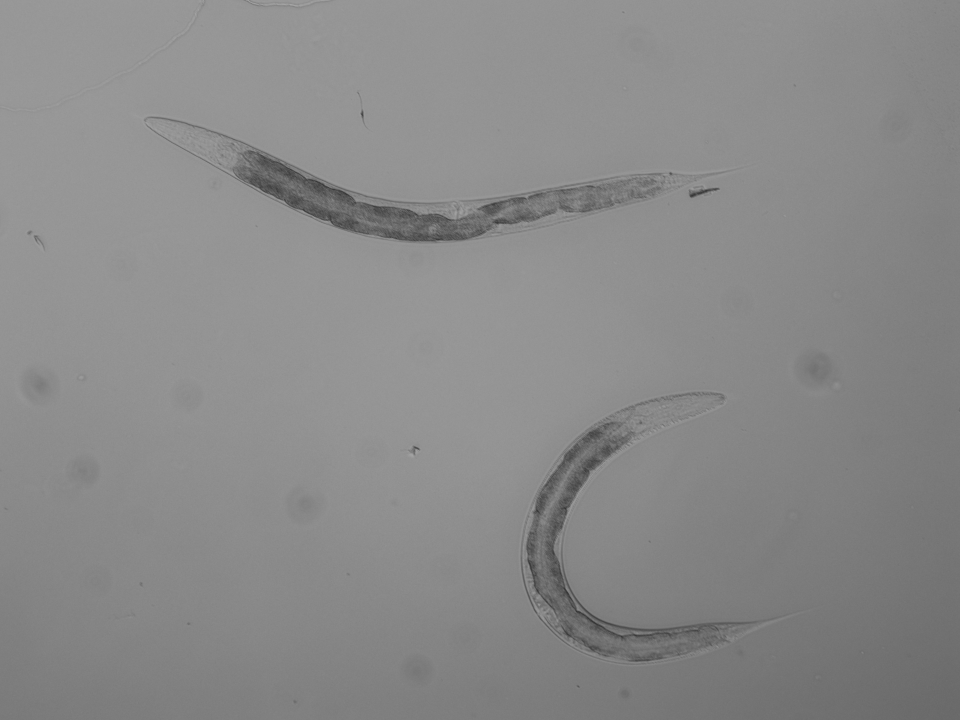

Supplement: Supplementary file 3 — Source data Fig. 3 [file 44321_2025_290_MOESM3_ESM.zip › Figure 3/Fig. 3E/Fig 3E_3_P316L het.tif]

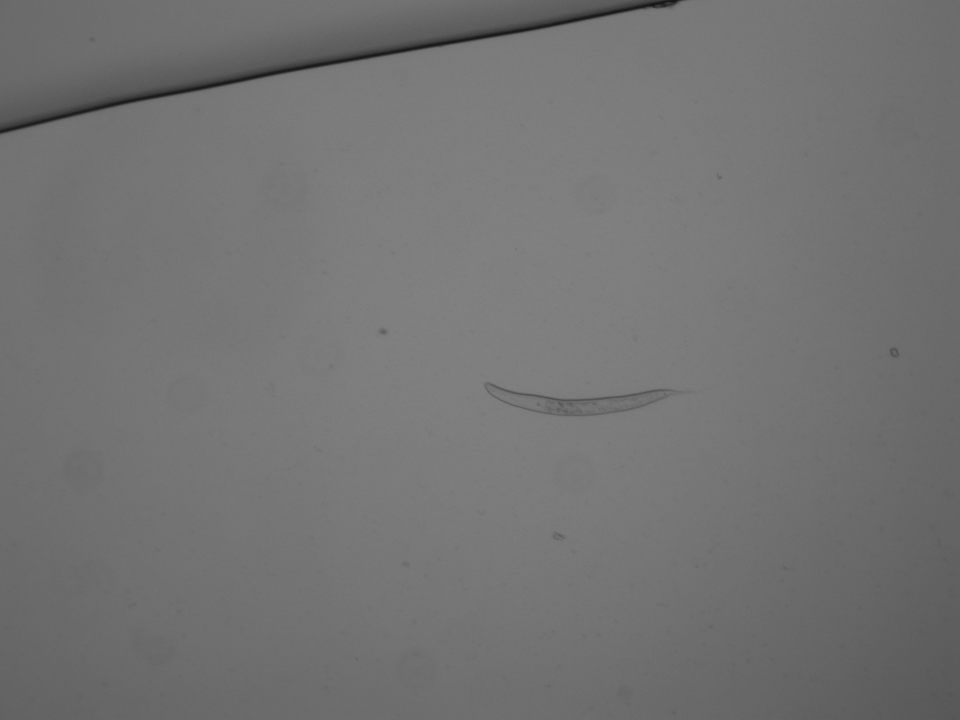

Supplement: Supplementary file 3 — Source data Fig. 3 [file 44321_2025_290_MOESM3_ESM.zip › Figure 3/Fig. 3E/Fig 3E_5_R167Q het.tif]

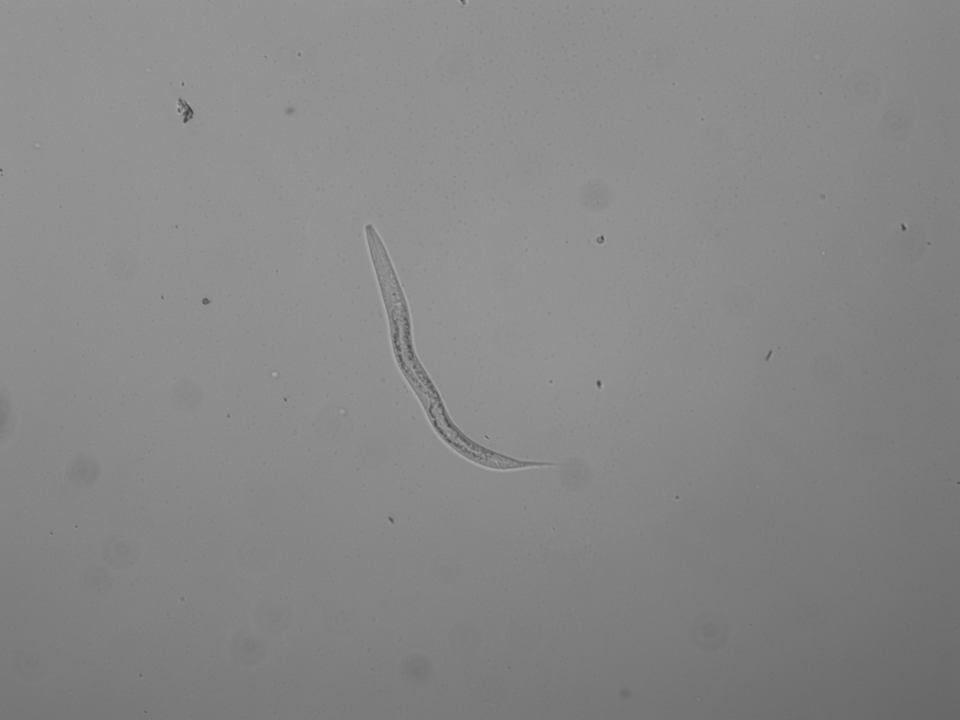

Supplement: Supplementary file 3 — Source data Fig. 3 [file 44321_2025_290_MOESM3_ESM.zip › Figure 3/Fig. 3E/Fig 3E_4_S331F het.tif]

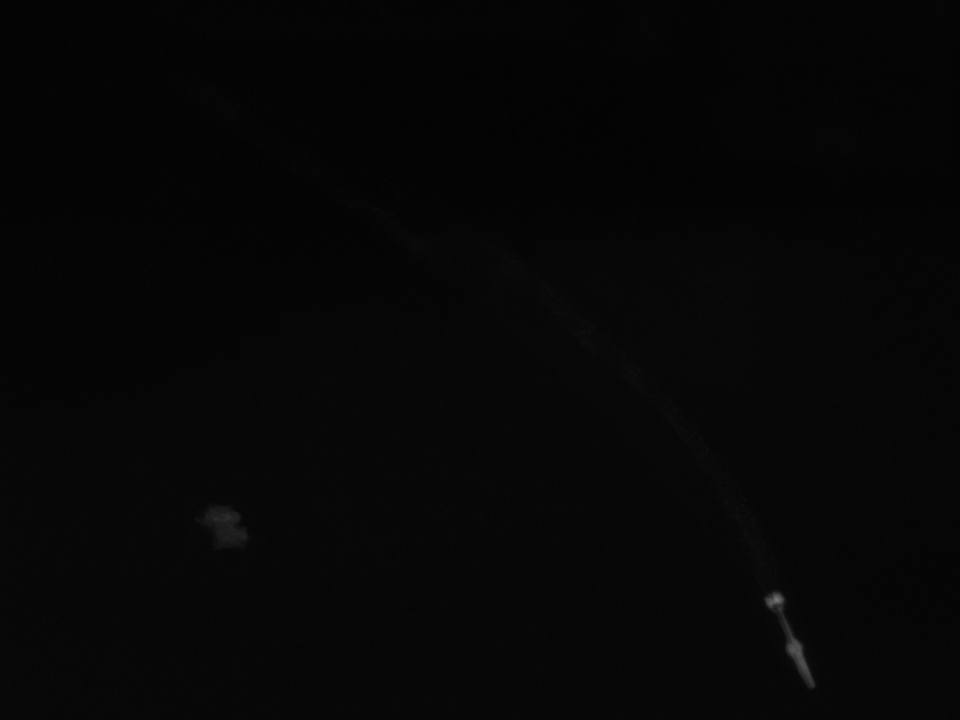

Supplement: Supplementary file 4 — Source data Fig. 4 [file 44321_2025_290_MOESM4_ESM.zip › Figure 4/Fig. 4E/Fig 4E_1_MitoStress_P316P.tif]

## Slide 1
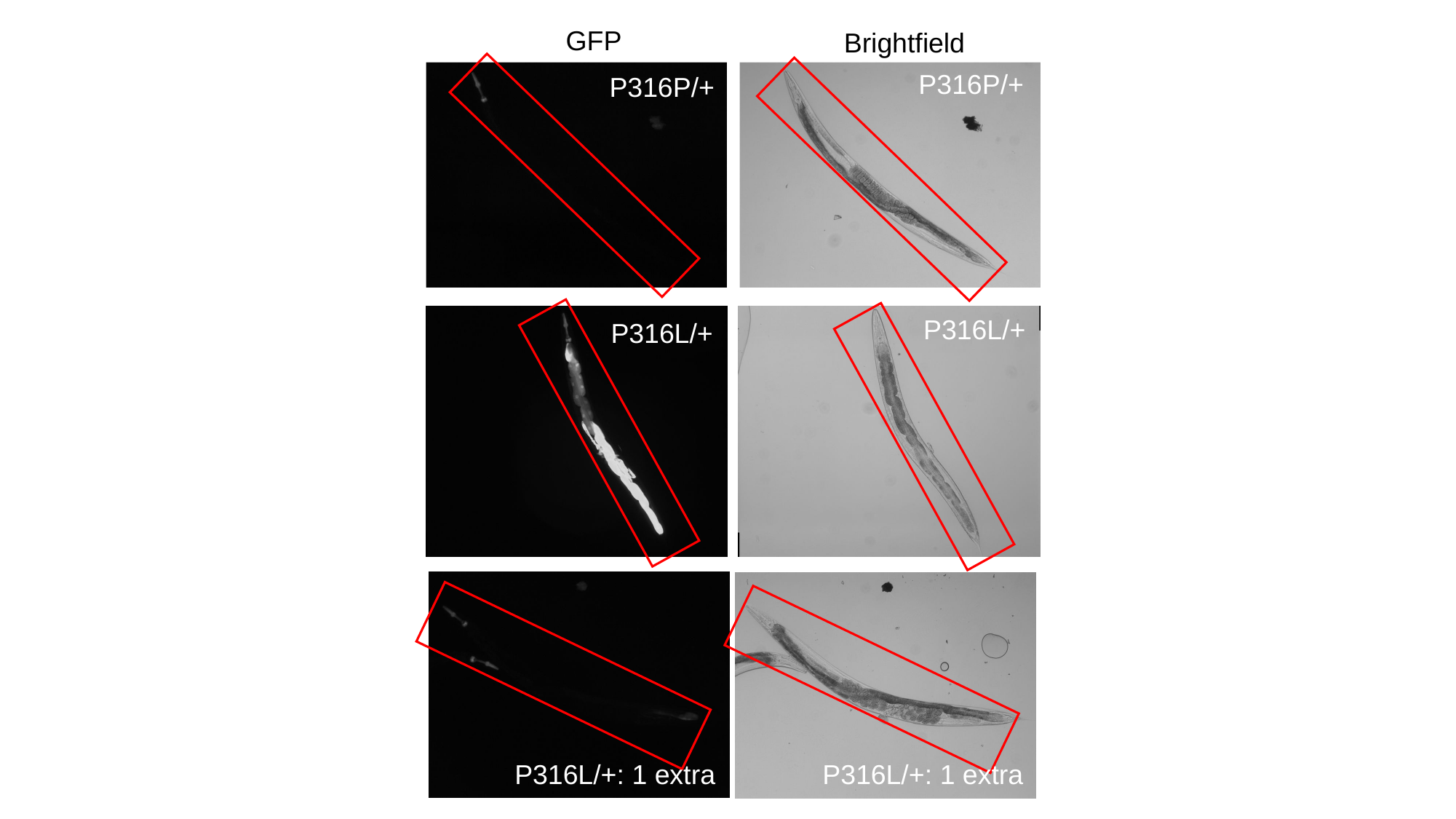

GFP
Brightfield
P316P/+
P316P/+
P316L/+
P316L/+
P316L/+: 1 extra
P316L/+: 1 extra

Supplement: Supplementary file 4 — Source data Fig. 4 [file 44321_2025_290_MOESM4_ESM.zip › Figure 4/Fig. 4E/Fig 4E_Image processing and cropping.pptx]

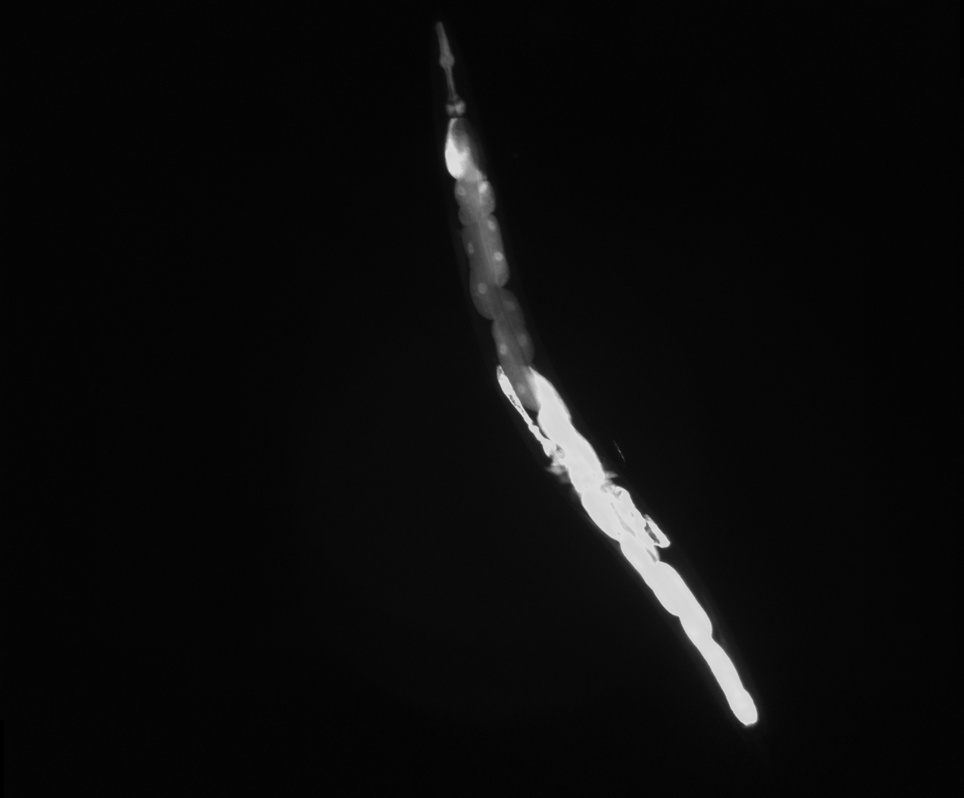

Supplement: Supplementary file 4 — Source data Fig. 4 [file 44321_2025_290_MOESM4_ESM.zip › Figure 4/Fig. 4E/Fig 4E_2_MitoStress_P316L.tif]

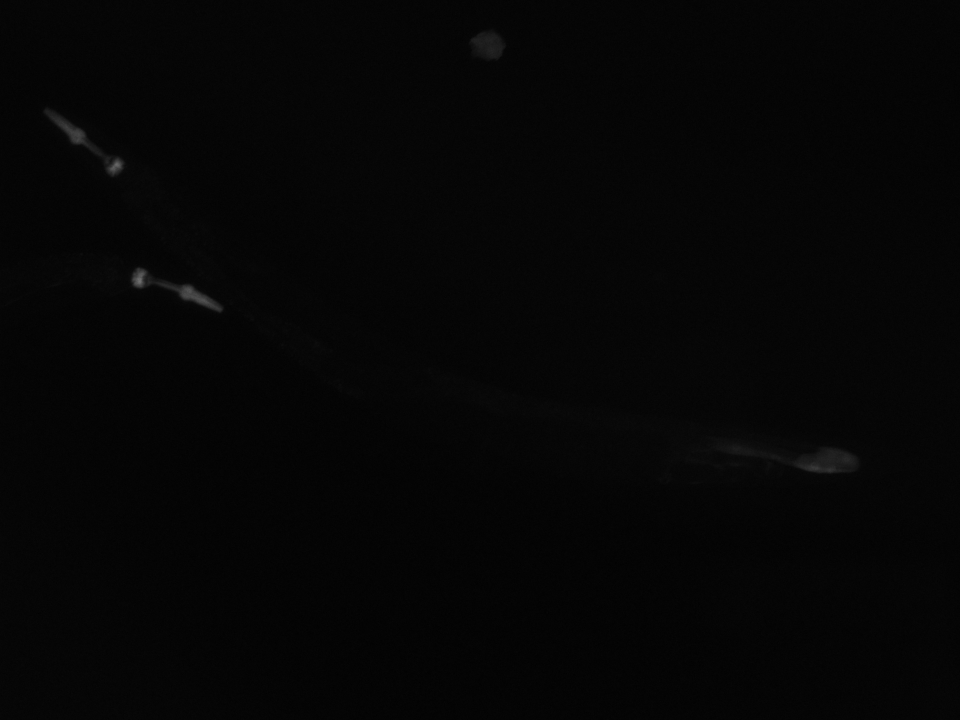

Supplement: Supplementary file 4 — Source data Fig. 4 [file 44321_2025_290_MOESM4_ESM.zip › Figure 4/Fig. 4E/Fig 4E_3_MitoStress_P316L_1extra.tif]

## Slide 1
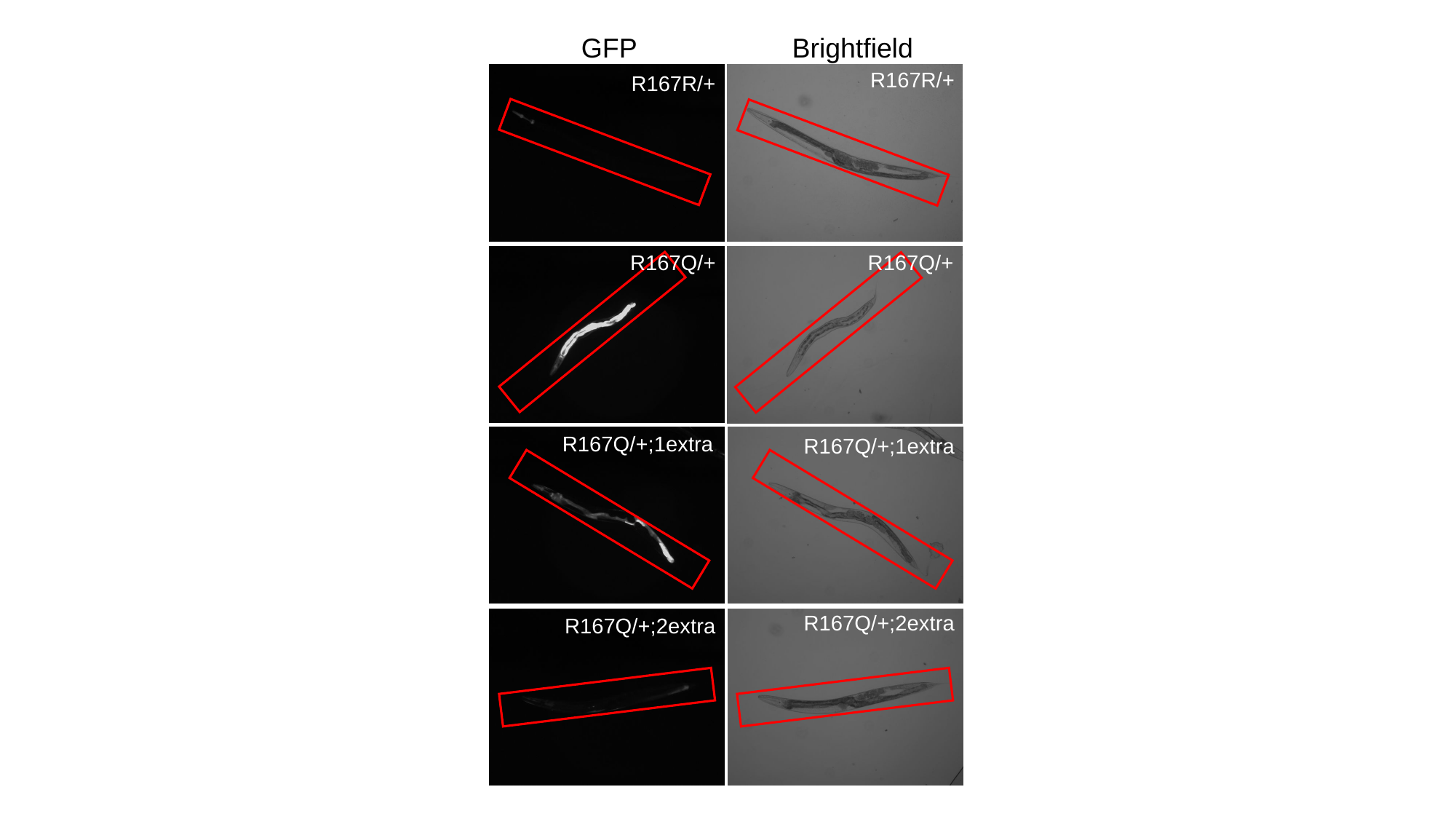

GFP
Brightfield
R167R/+
R167R/+
R167Q/+
R167Q/+
R167Q/+;1extra
R167Q/+;1extra
R167Q/+;2extra
R167Q/+;2extra

Supplement: Supplementary file 4 — Source data Fig. 4 [file 44321_2025_290_MOESM4_ESM.zip › Figure 4/Fig. 4G/Fig 4G_Image processing and cropping.pptx]

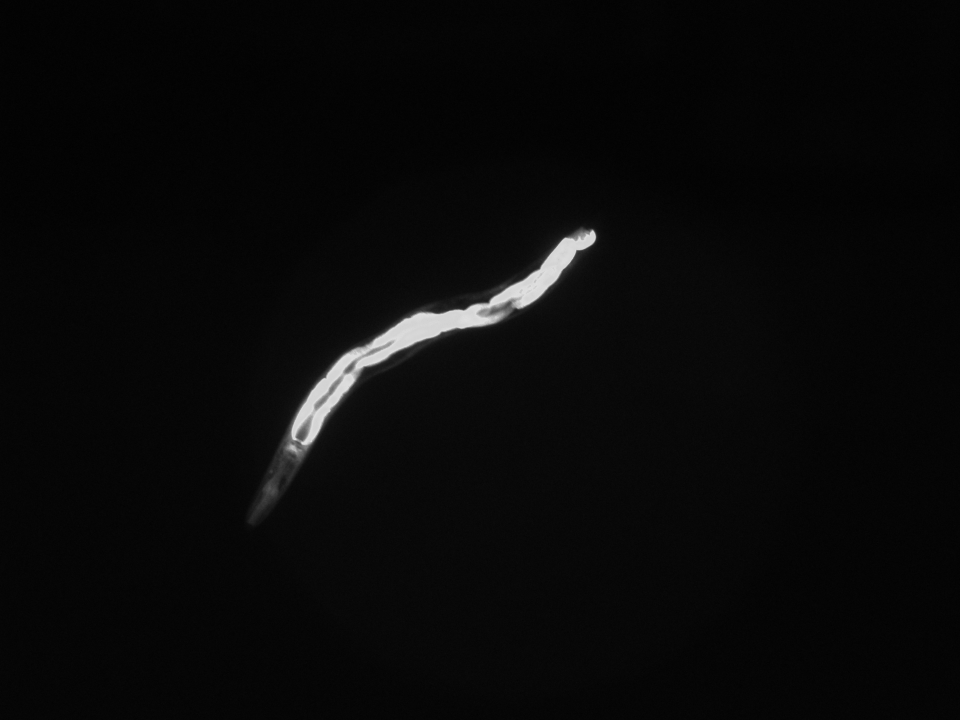

Supplement: Supplementary file 4 — Source data Fig. 4 [file 44321_2025_290_MOESM4_ESM.zip › Figure 4/Fig. 4G/Fig 4G_2_MitoStress_R167Q.tif]

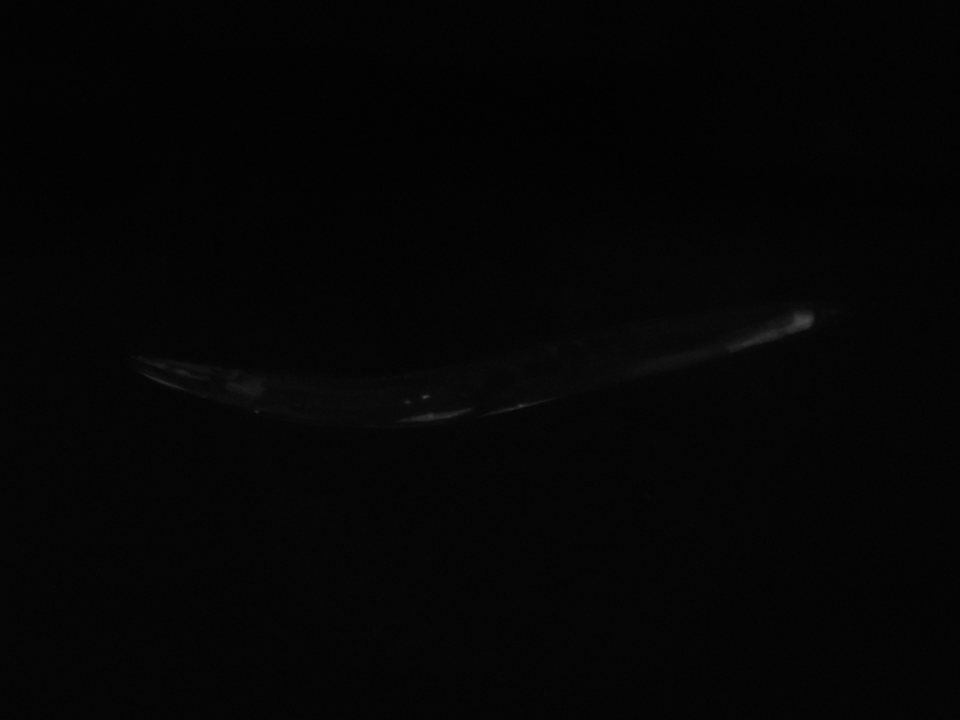

Supplement: Supplementary file 4 — Source data Fig. 4 [file 44321_2025_290_MOESM4_ESM.zip › Figure 4/Fig. 4G/Fig 4G_4_MitoStress_R167Q_2extra.tif]

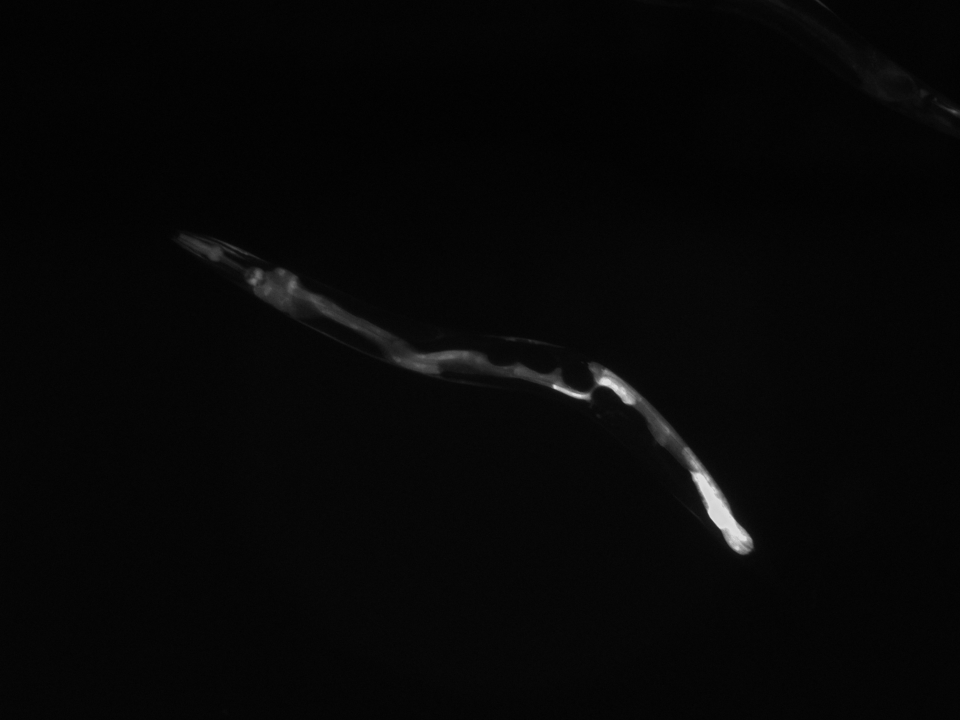

Supplement: Supplementary file 4 — Source data Fig. 4 [file 44321_2025_290_MOESM4_ESM.zip › Figure 4/Fig. 4G/Fig 4G_3_MitoStress_R167Q_1extra.tif]

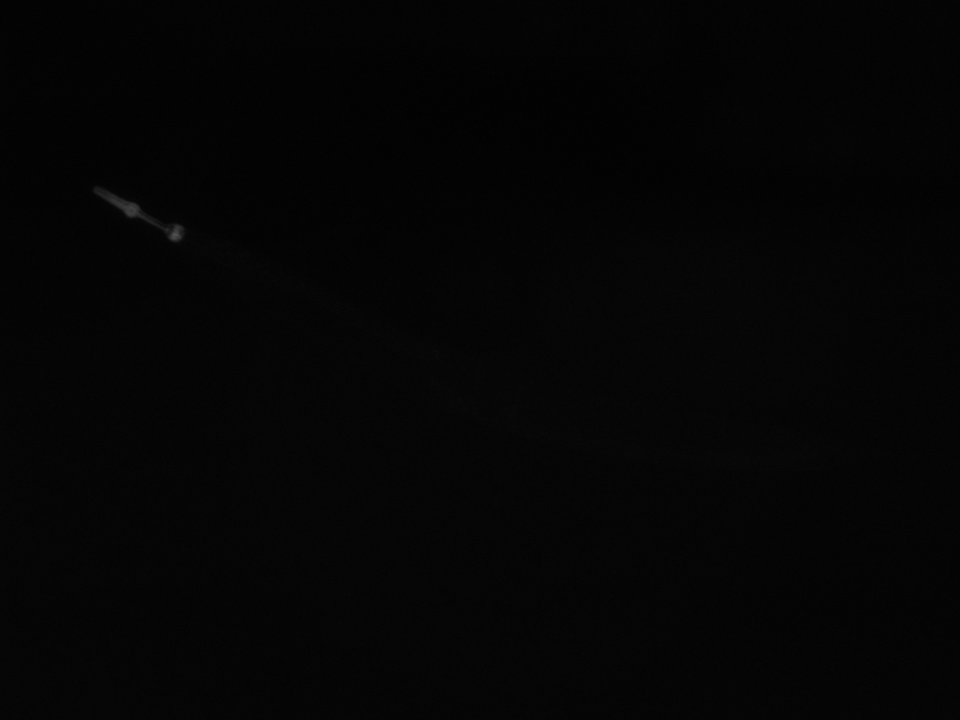

Supplement: Supplementary file 4 — Source data Fig. 4 [file 44321_2025_290_MOESM4_ESM.zip › Figure 4/Fig. 4G/Fig 4G_1_MitoStress_R167R.tif]

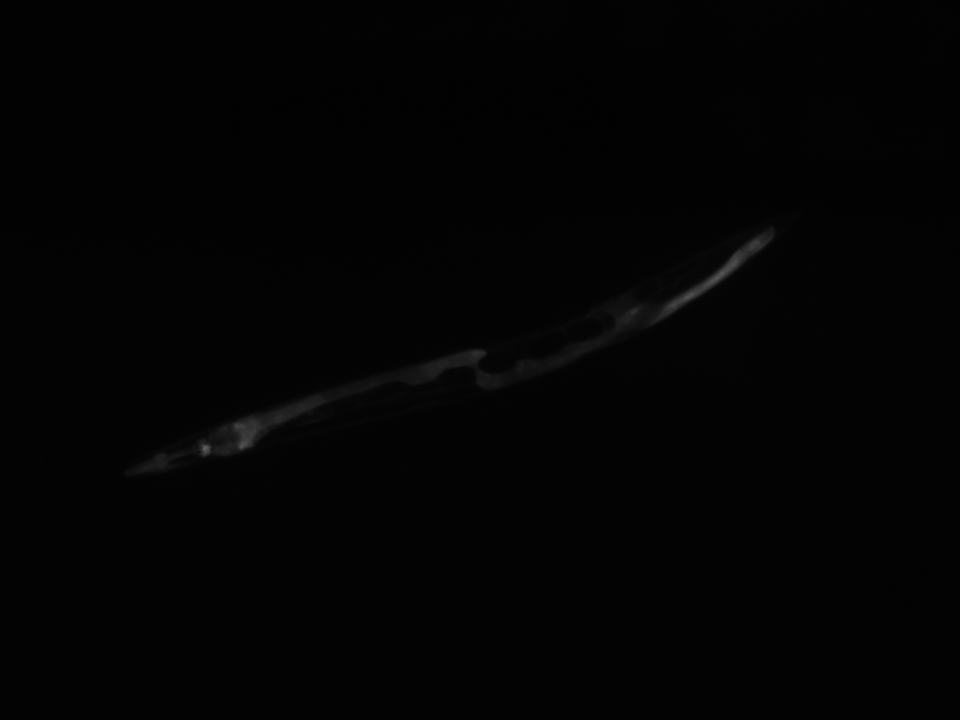

Supplement: Supplementary file 4 — Source data Fig. 4 [file 44321_2025_290_MOESM4_ESM.zip › Figure 4/Fig. 4F/Fig 4F_3_MitoStress_S331F_1extra.tif]

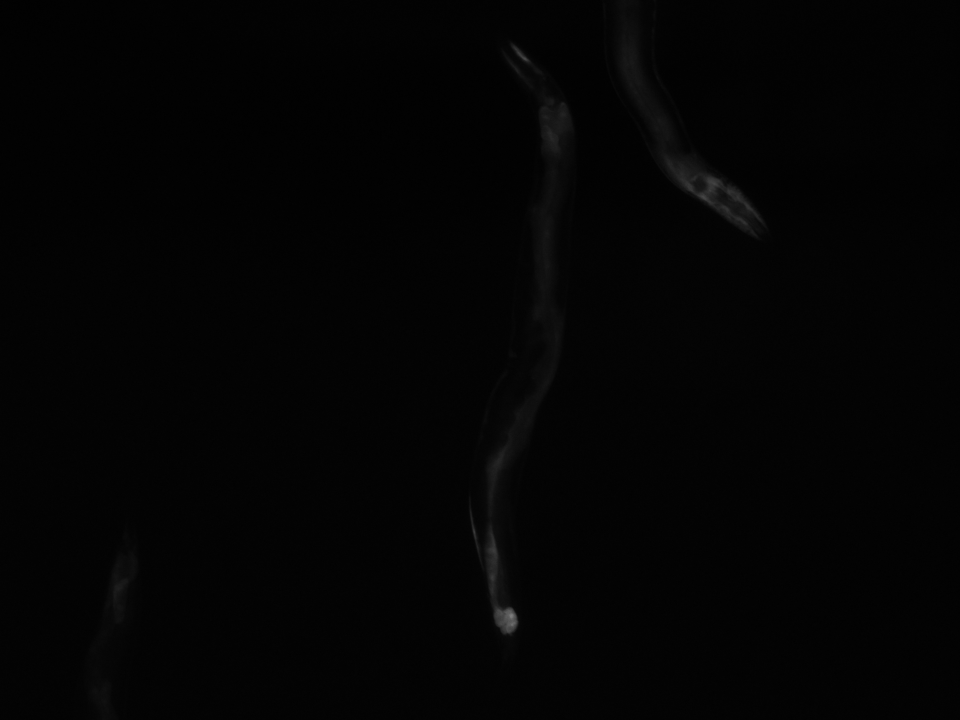

Supplement: Supplementary file 4 — Source data Fig. 4 [file 44321_2025_290_MOESM4_ESM.zip › Figure 4/Fig. 4F/Fig 4F_4_MitoStress_S331F_2extra.tif]

## Slide 1
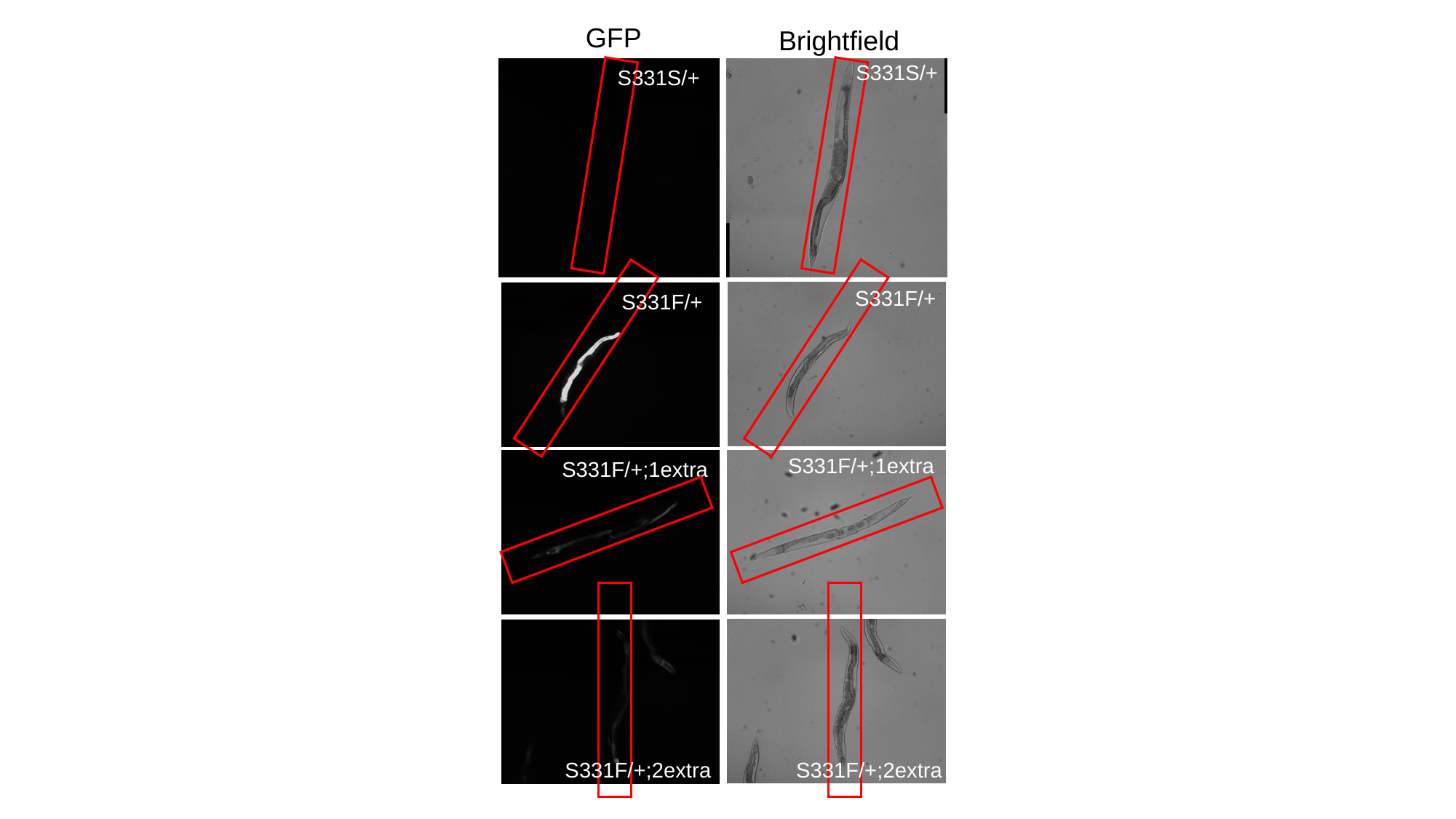

GFP
Brightfield
S331S/+
S331S/+
S331F/+
S331F/+
S331F/+;1extra
S331F/+;1extra
S331F/+;2extra
S331F/+;2extra

Supplement: Supplementary file 4 — Source data Fig. 4 [file 44321_2025_290_MOESM4_ESM.zip › Figure 4/Fig. 4F/Fig 4F_Image processing and cropping.pptx]

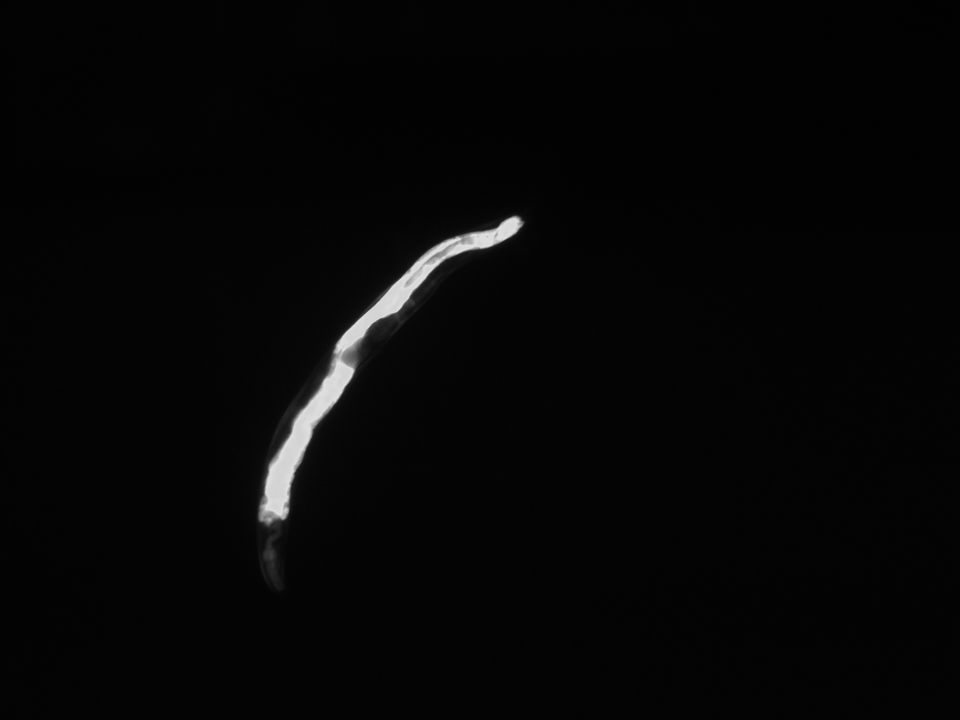

Supplement: Supplementary file 4 — Source data Fig. 4 [file 44321_2025_290_MOESM4_ESM.zip › Figure 4/Fig. 4F/Fig 4F_2_MitoStress_S331F.tif]

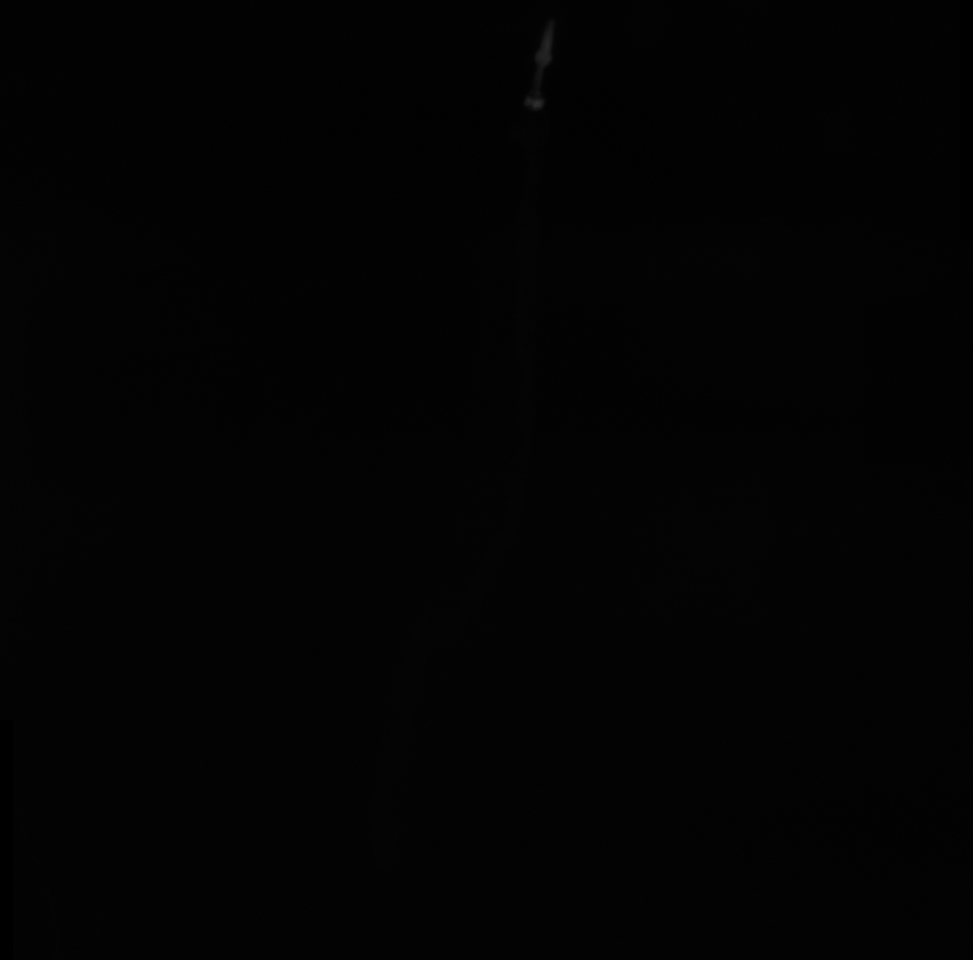

Supplement: Supplementary file 4 — Source data Fig. 4 [file 44321_2025_290_MOESM4_ESM.zip › Figure 4/Fig. 4F/Fig 4F_1_MitoStress_S331S.tif]

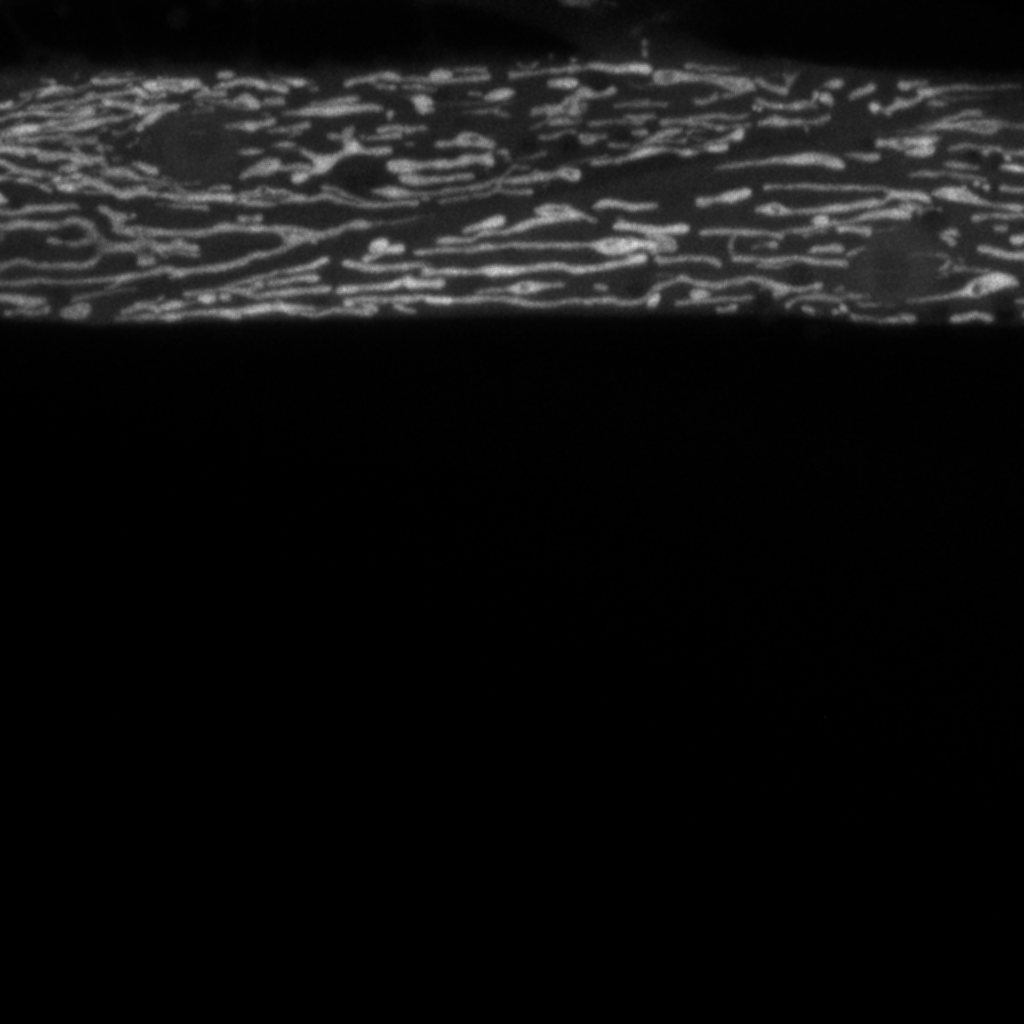

Supplement: Supplementary file 5 — Source data Fig. 5 [file 44321_2025_290_MOESM5_ESM.zip › Figure 5/Fig. 5A/Fig 5A_2_del_mitochondria.tif]

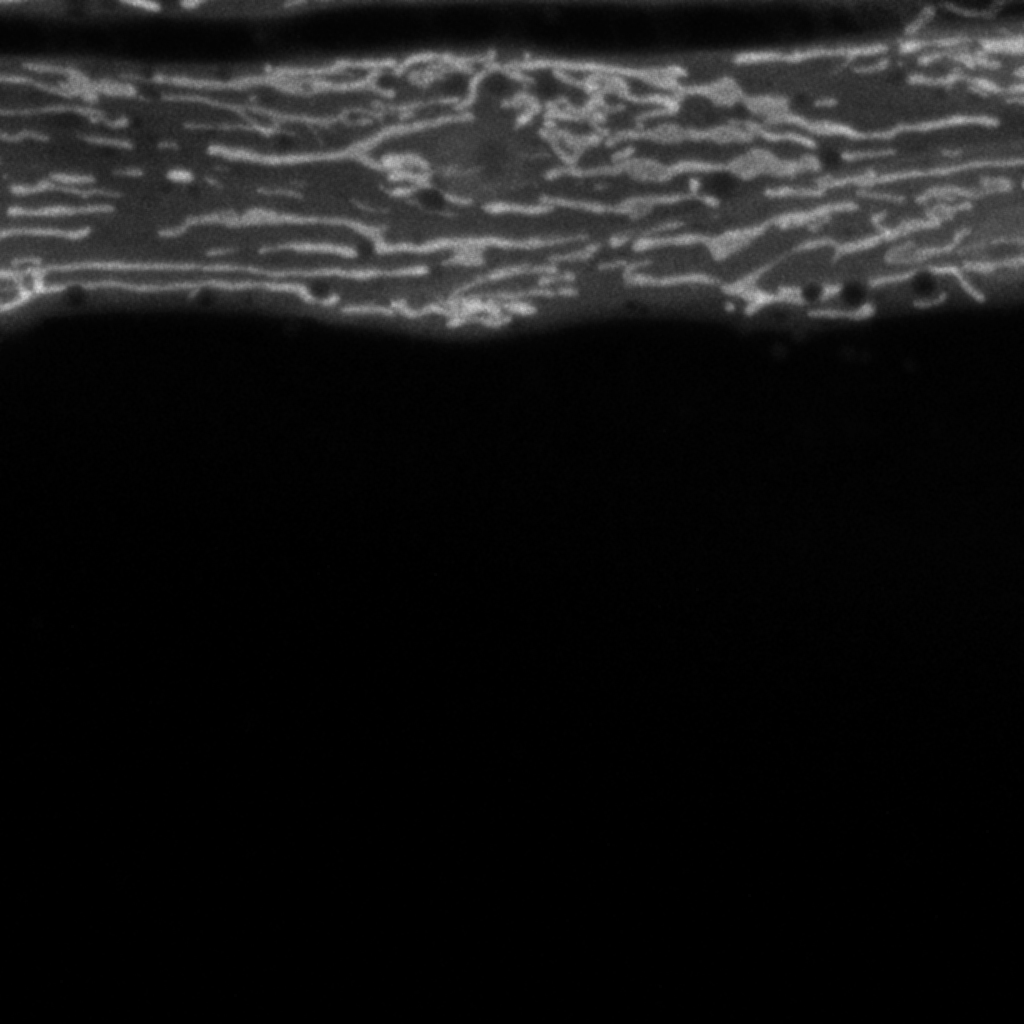

Supplement: Supplementary file 5 — Source data Fig. 5 [file 44321_2025_290_MOESM5_ESM.zip › Figure 5/Fig. 5A/Fig 5A_1_P316P_mitochondria.tif]

## Slide 1
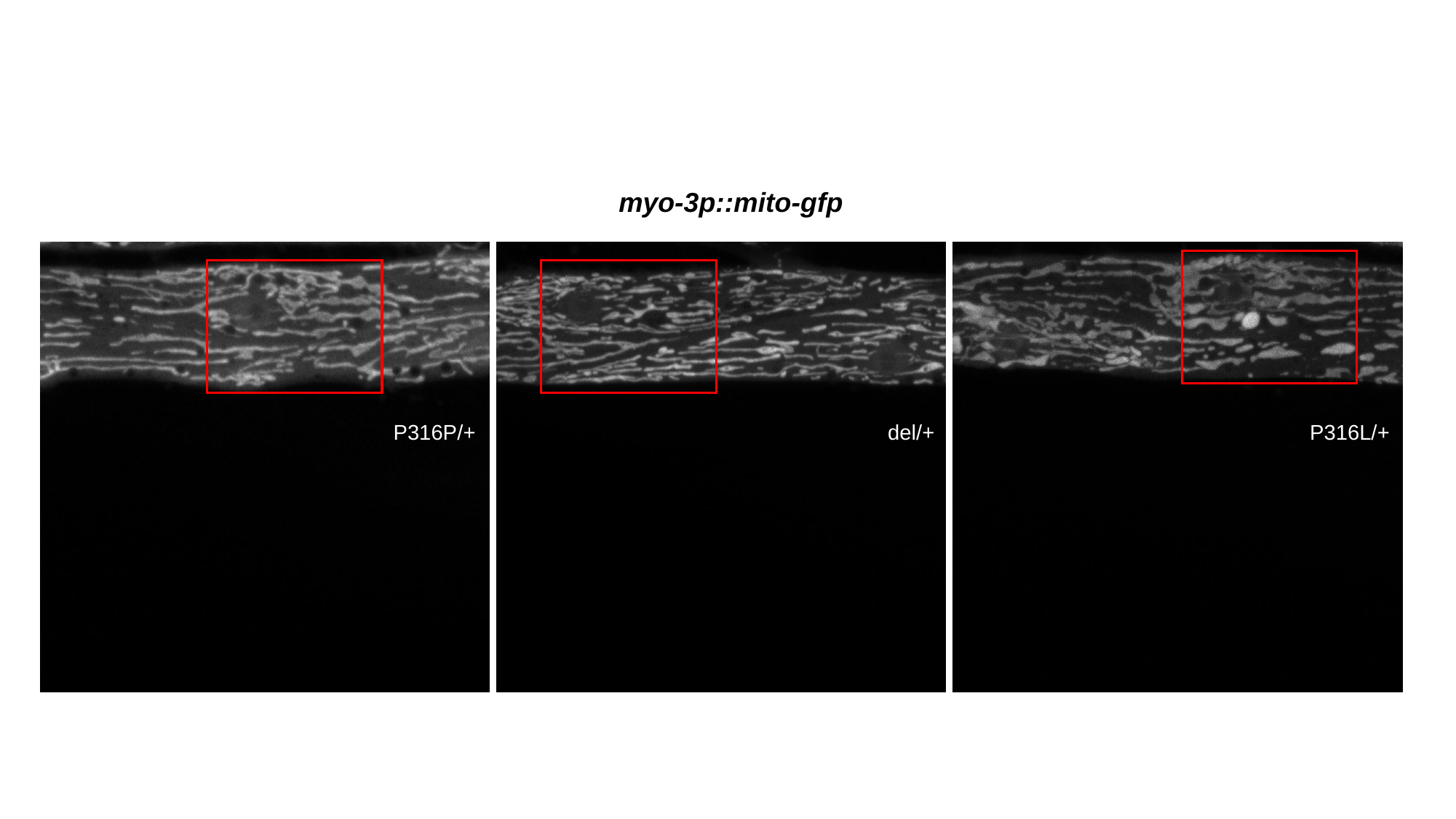

myo-3p::mito-gfp
P316P/+
del/+
P316L/+

Supplement: Supplementary file 5 — Source data Fig. 5 [file 44321_2025_290_MOESM5_ESM.zip › Figure 5/Fig. 5A/Fig 5A_Image processing and cropping.pptx]

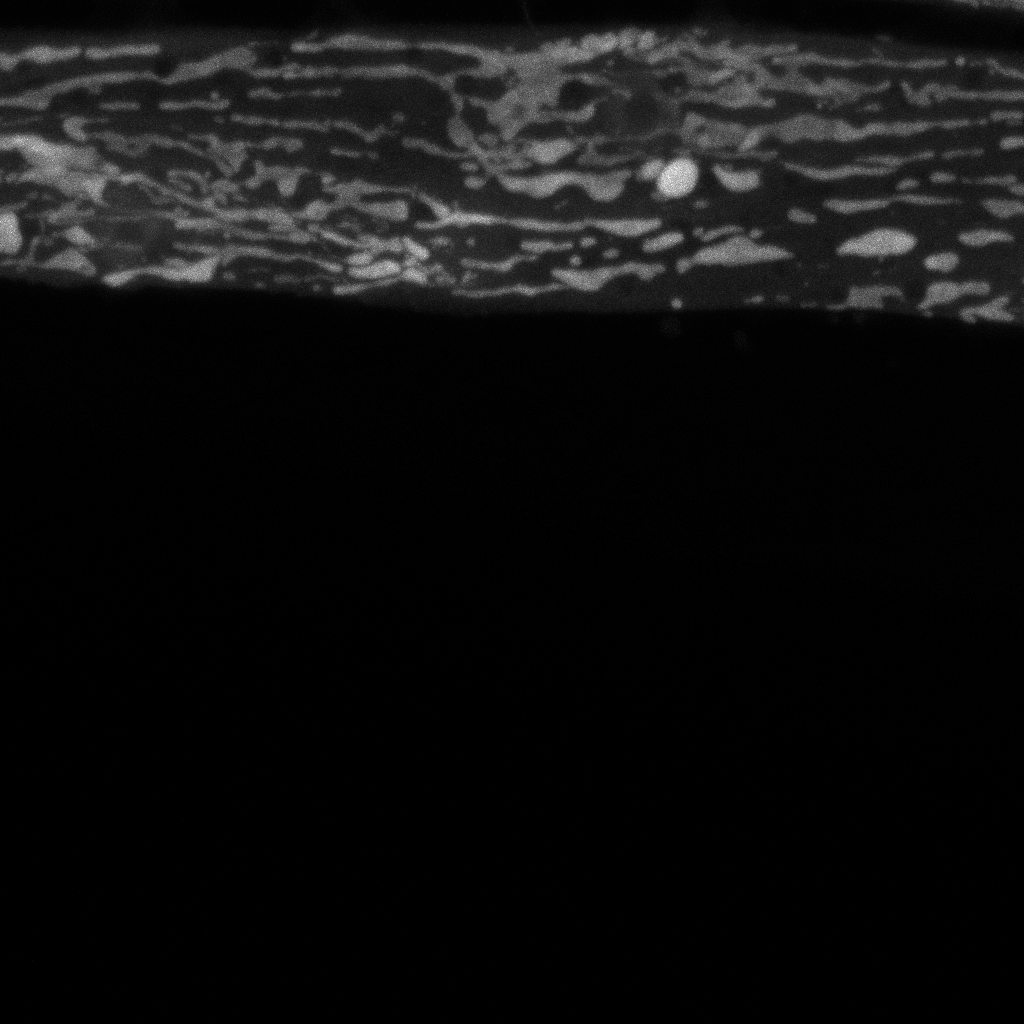

Supplement: Supplementary file 5 — Source data Fig. 5 [file 44321_2025_290_MOESM5_ESM.zip › Figure 5/Fig. 5A/Fig 5A_3_P316L_mitochondria.tif]

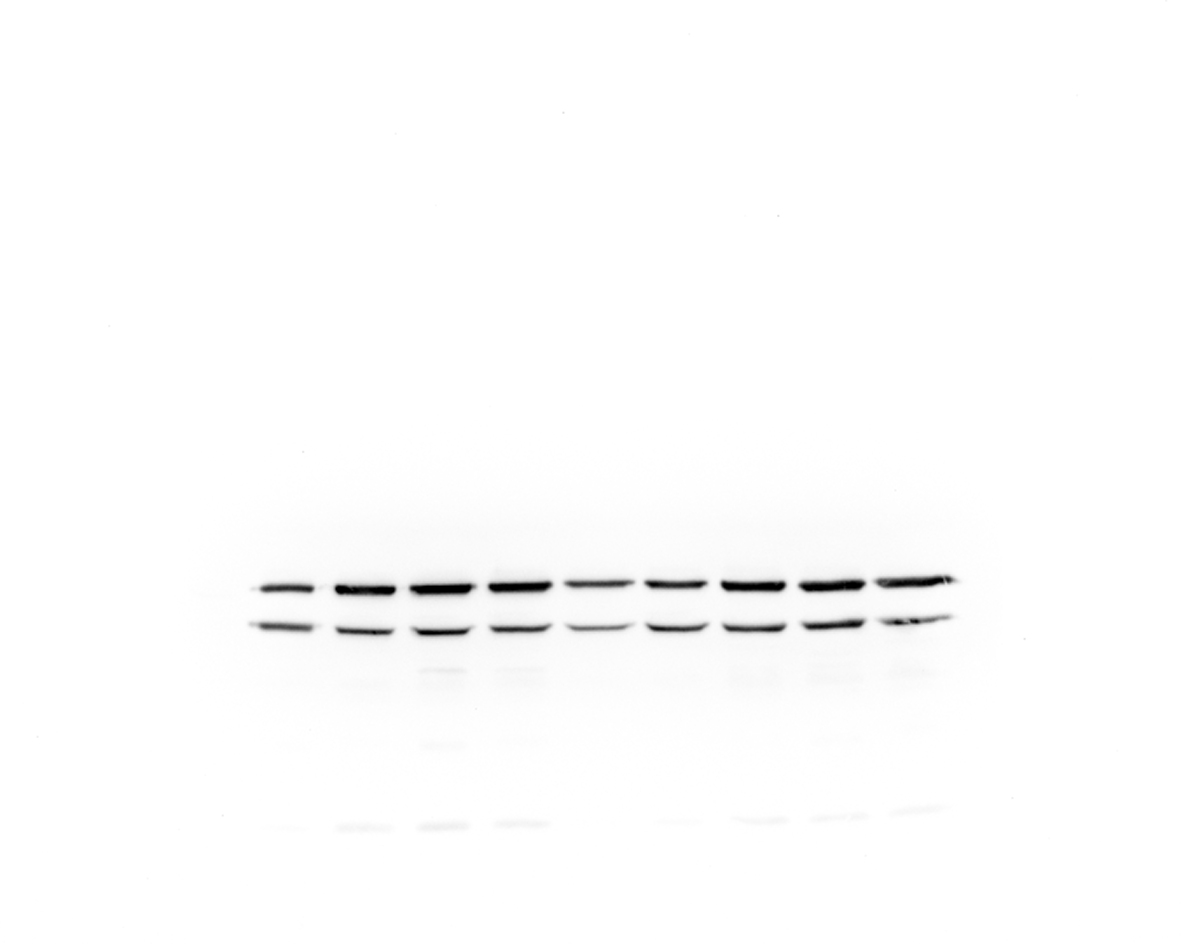

Supplement: Supplementary file 6 — Source data Fig. 6 [file 44321_2025_290_MOESM6_ESM.zip › Figure 6/Fig. 6C/Fig 6C_ATP5F1B .tif]

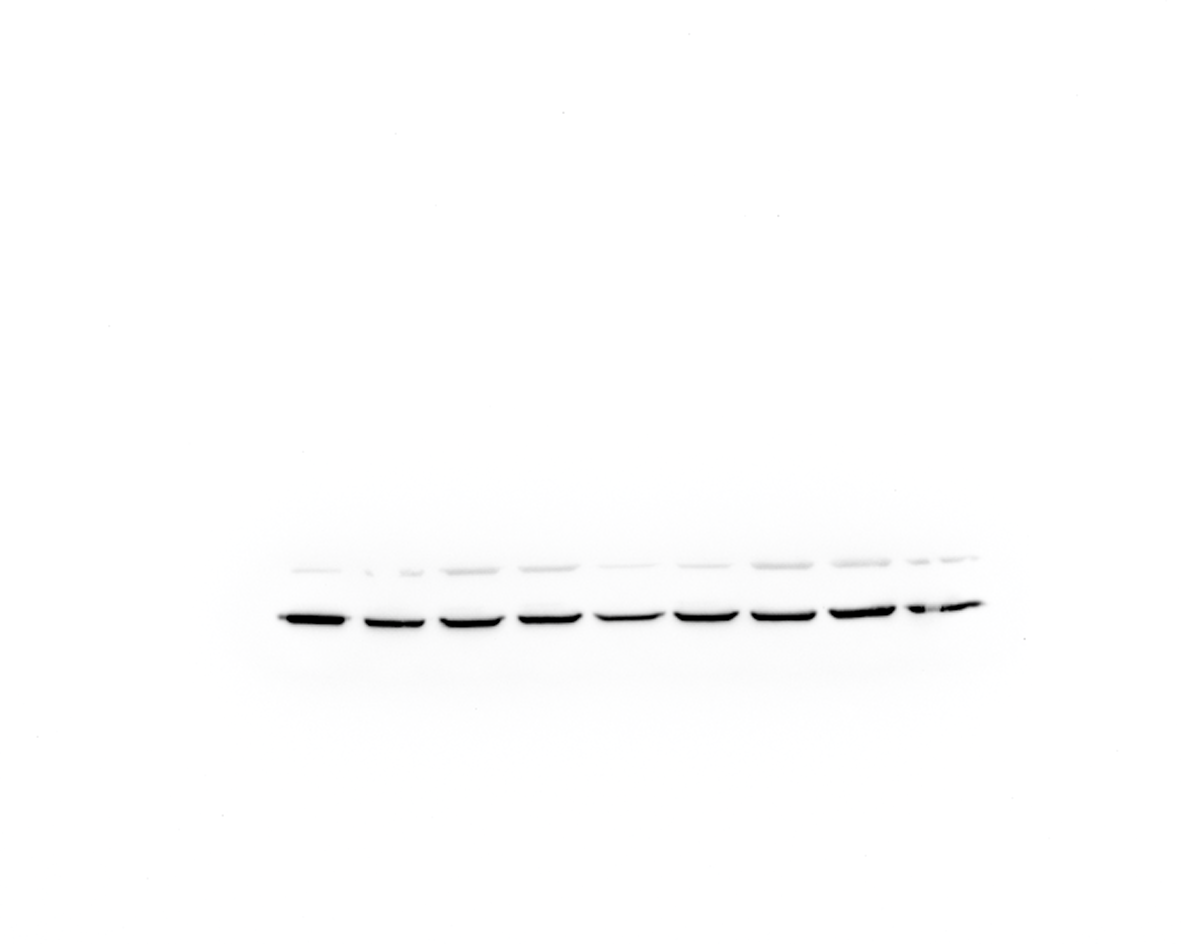

Supplement: Supplementary file 6 — Source data Fig. 6 [file 44321_2025_290_MOESM6_ESM.zip › Figure 6/Fig. 6C/Fig 6C_Citrate Synthase.tif]

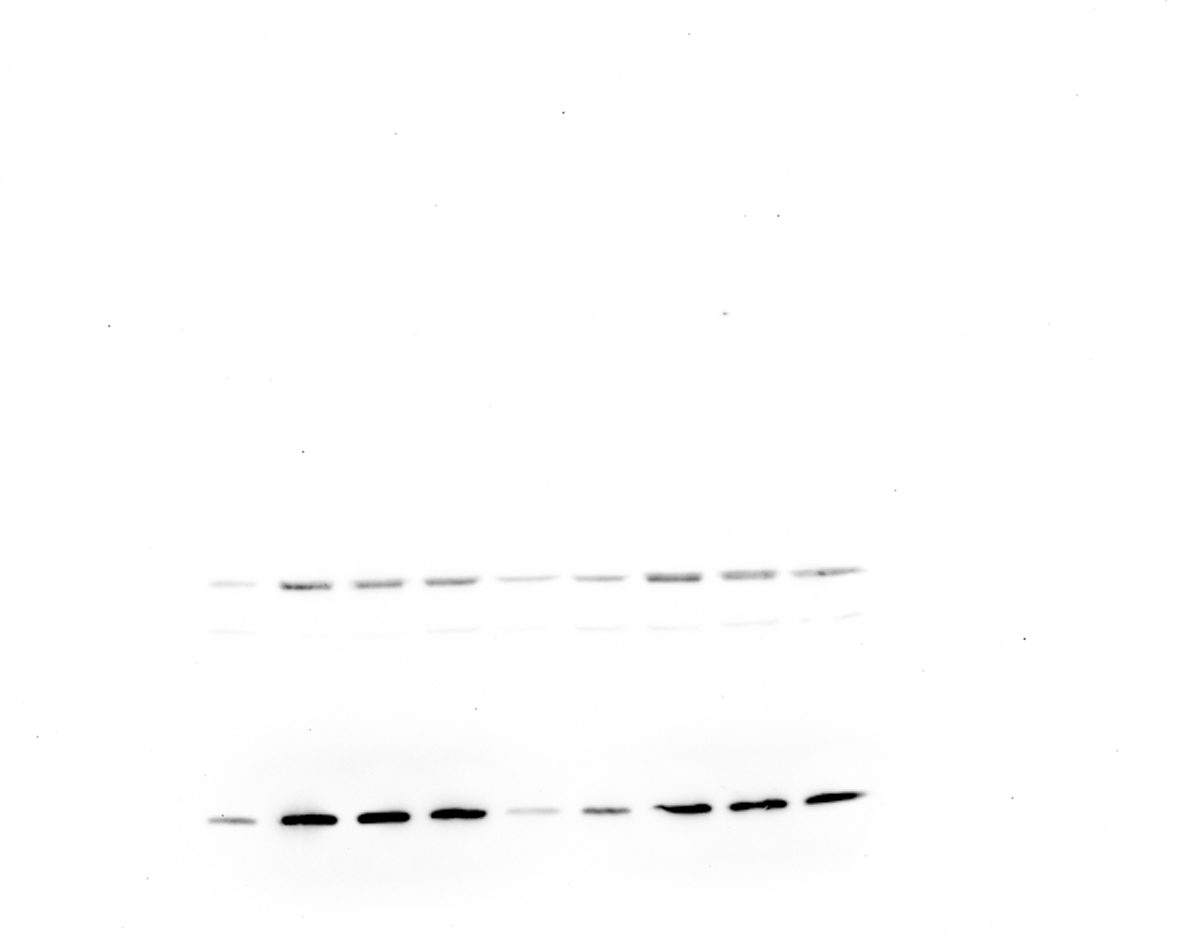

Supplement: Supplementary file 6 — Source data Fig. 6 [file 44321_2025_290_MOESM6_ESM.zip › Figure 6/Fig. 6C/Fig 6C_ATP5O.tif]

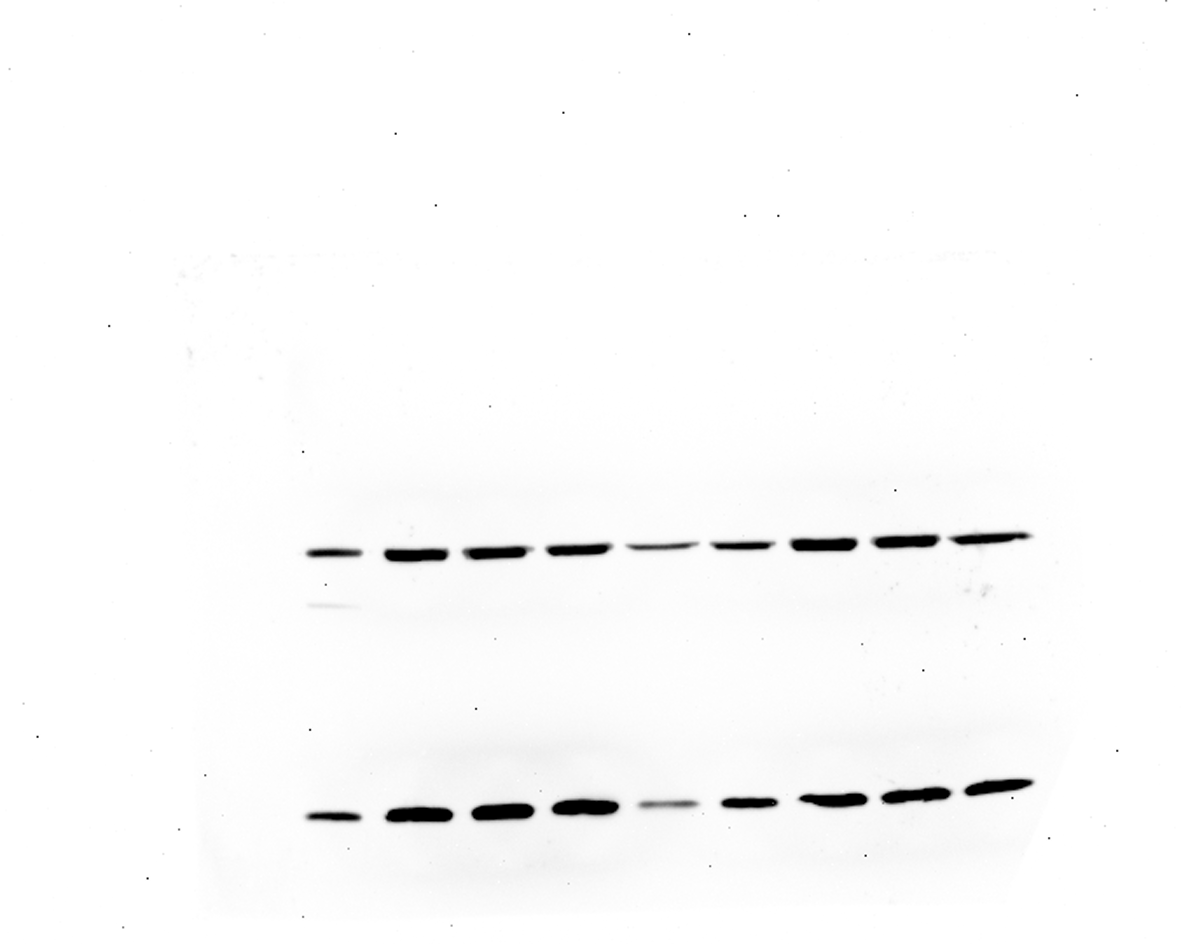

Supplement: Supplementary file 6 — Source data Fig. 6 [file 44321_2025_290_MOESM6_ESM.zip › Figure 6/Fig. 6C/Fig 6C_ATP5F1A .tif]

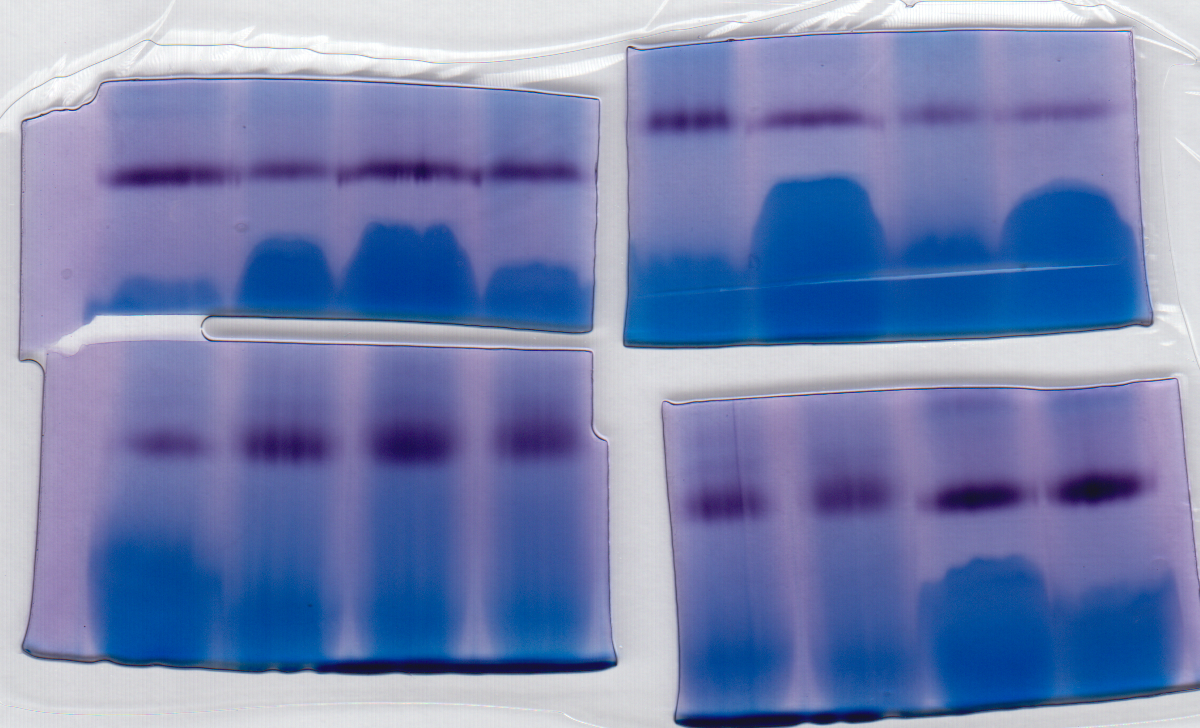

Supplement: Supplementary file 6 — Source data Fig. 6 [file 44321_2025_290_MOESM6_ESM.zip › Figure 6/Fig. 6B/Fig 6B_Proband 1_Complex II.tif]

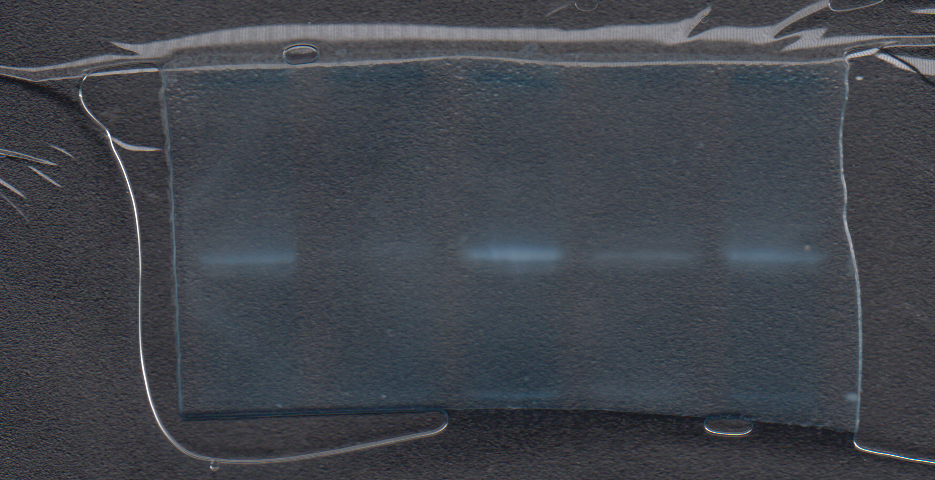

Supplement: Supplementary file 6 — Source data Fig. 6 [file 44321_2025_290_MOESM6_ESM.zip › Figure 6/Fig. 6B/Fig 6B_Proband 6_Complex V.tif]

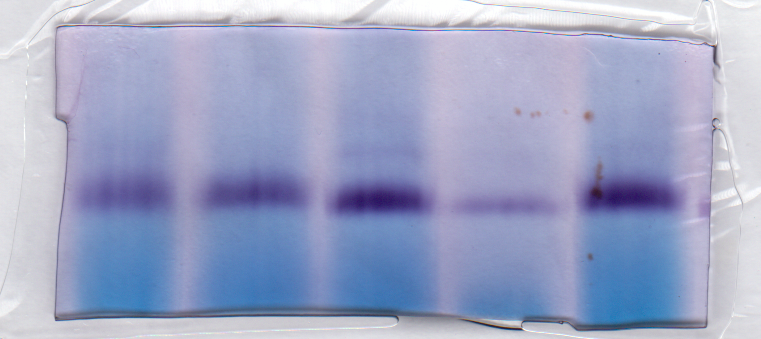

Supplement: Supplementary file 6 — Source data Fig. 6 [file 44321_2025_290_MOESM6_ESM.zip › Figure 6/Fig. 6B/Fig 6B_Proband 6_Complex II.tif]

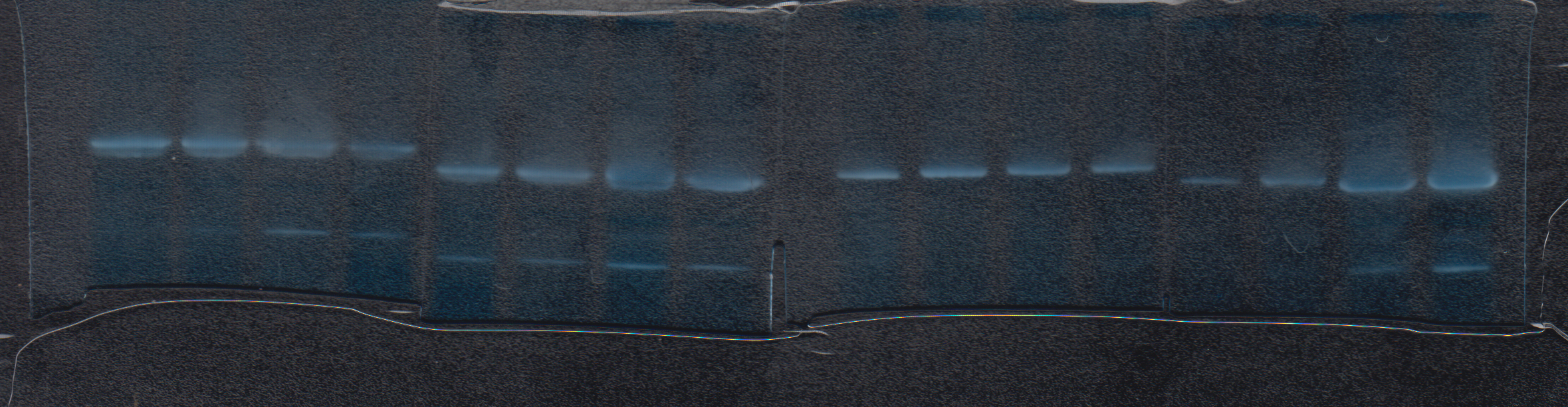

Supplement: Supplementary file 6 — Source data Fig. 6 [file 44321_2025_290_MOESM6_ESM.zip › Figure 6/Fig. 6B/Fig 6B_Proband 1_Complex V.tif]

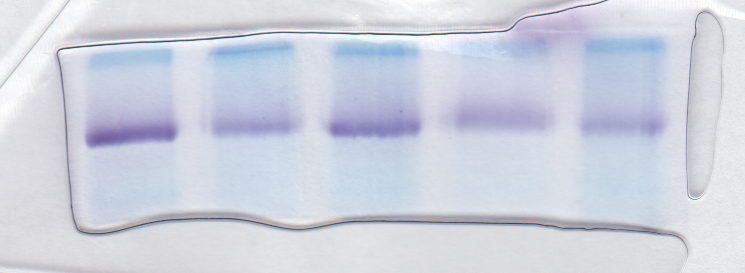

Supplement: Supplementary file 6 — Source data Fig. 6 [file 44321_2025_290_MOESM6_ESM.zip › Figure 6/Fig. 6B/Fig 6B_Proband 6_Complex I.tif]

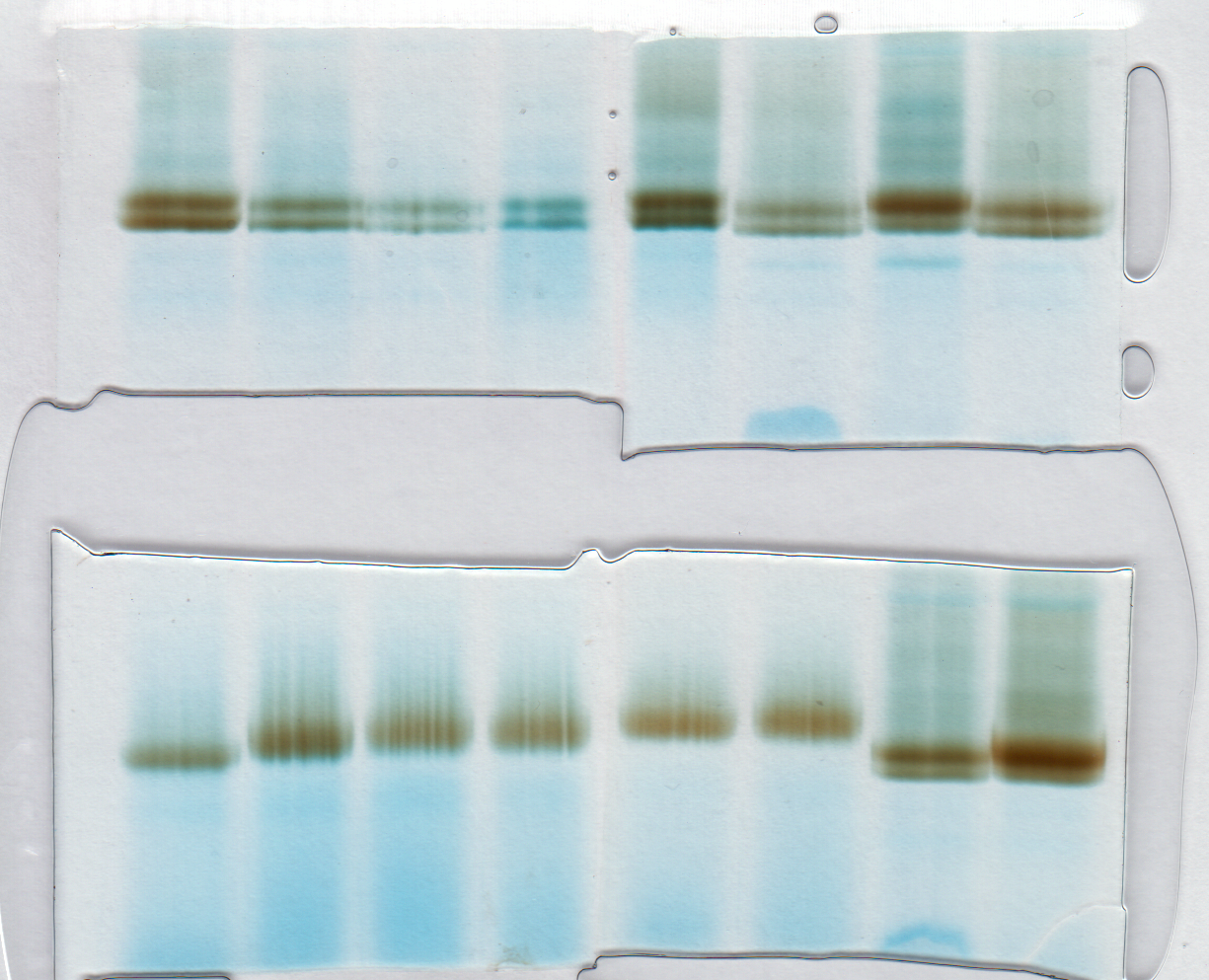

Supplement: Supplementary file 6 — Source data Fig. 6 [file 44321_2025_290_MOESM6_ESM.zip › Figure 6/Fig. 6B/Fig 6B_Proband 1_Complex IV.tif]

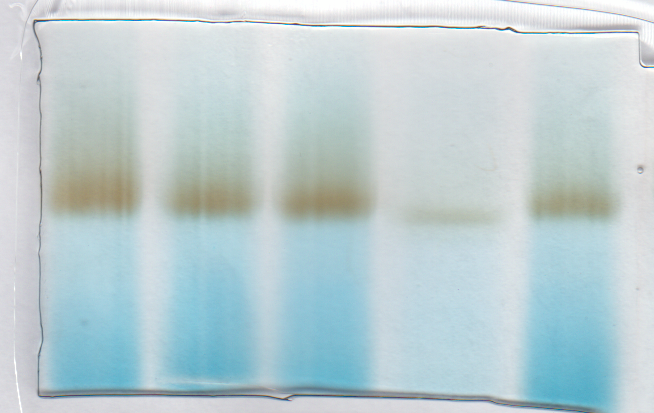

Supplement: Supplementary file 6 — Source data Fig. 6 [file 44321_2025_290_MOESM6_ESM.zip › Figure 6/Fig. 6B/Fig 6B_Proband 6_Complex IV.tif]

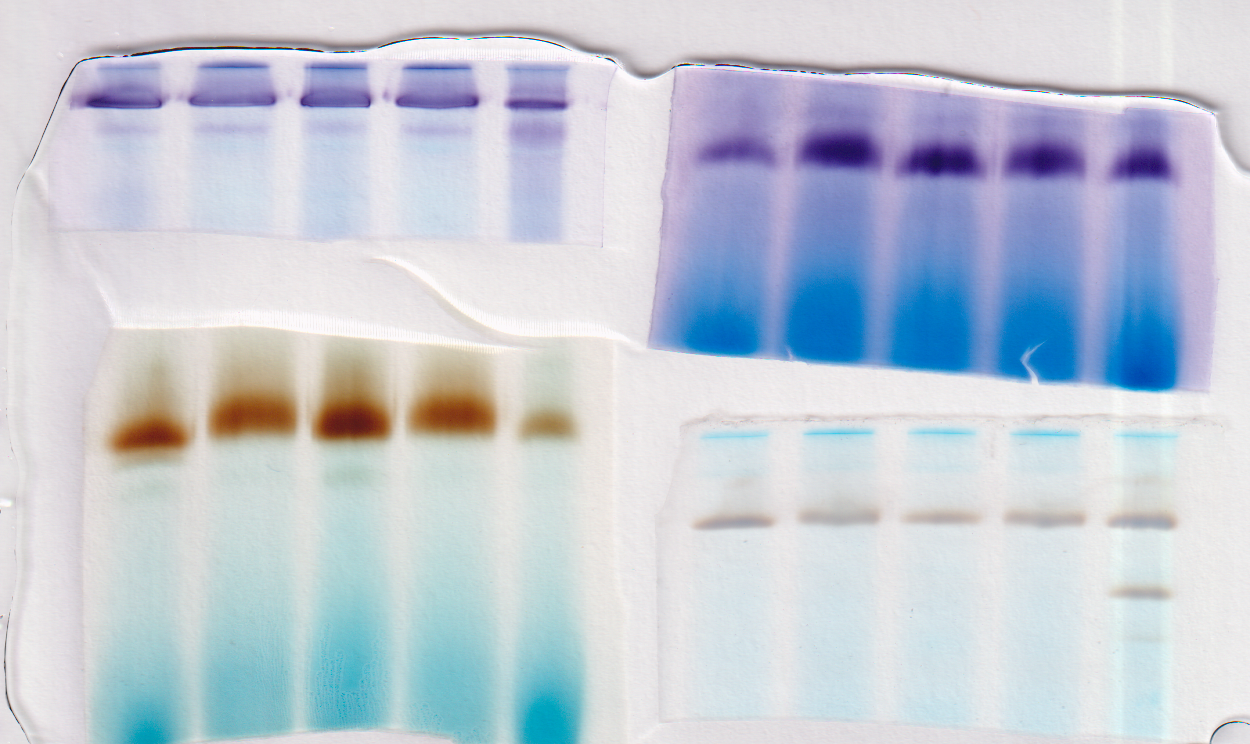

Supplement: Supplementary file 6 — Source data Fig. 6 [file 44321_2025_290_MOESM6_ESM.zip › Figure 6/Fig. 6B/Fig 6B_Proband 4_Complexes I II IV V.tif]

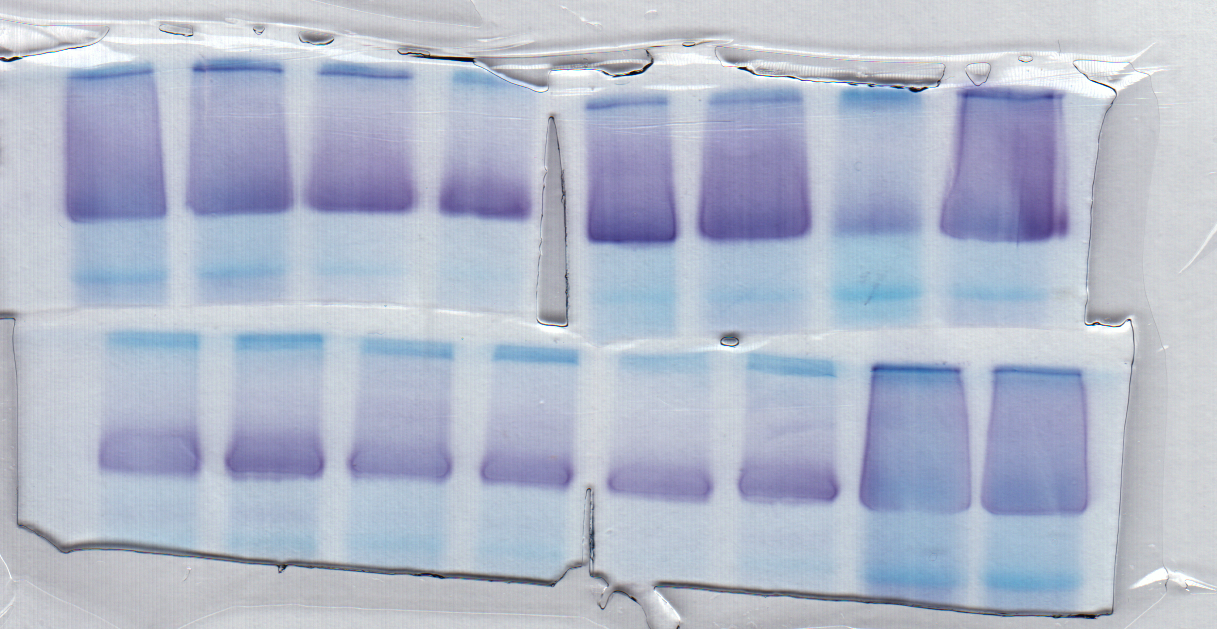

Supplement: Supplementary file 6 — Source data Fig. 6 [file 44321_2025_290_MOESM6_ESM.zip › Figure 6/Fig. 6B/Fig 6B_Proband 1_Complex I.tif]

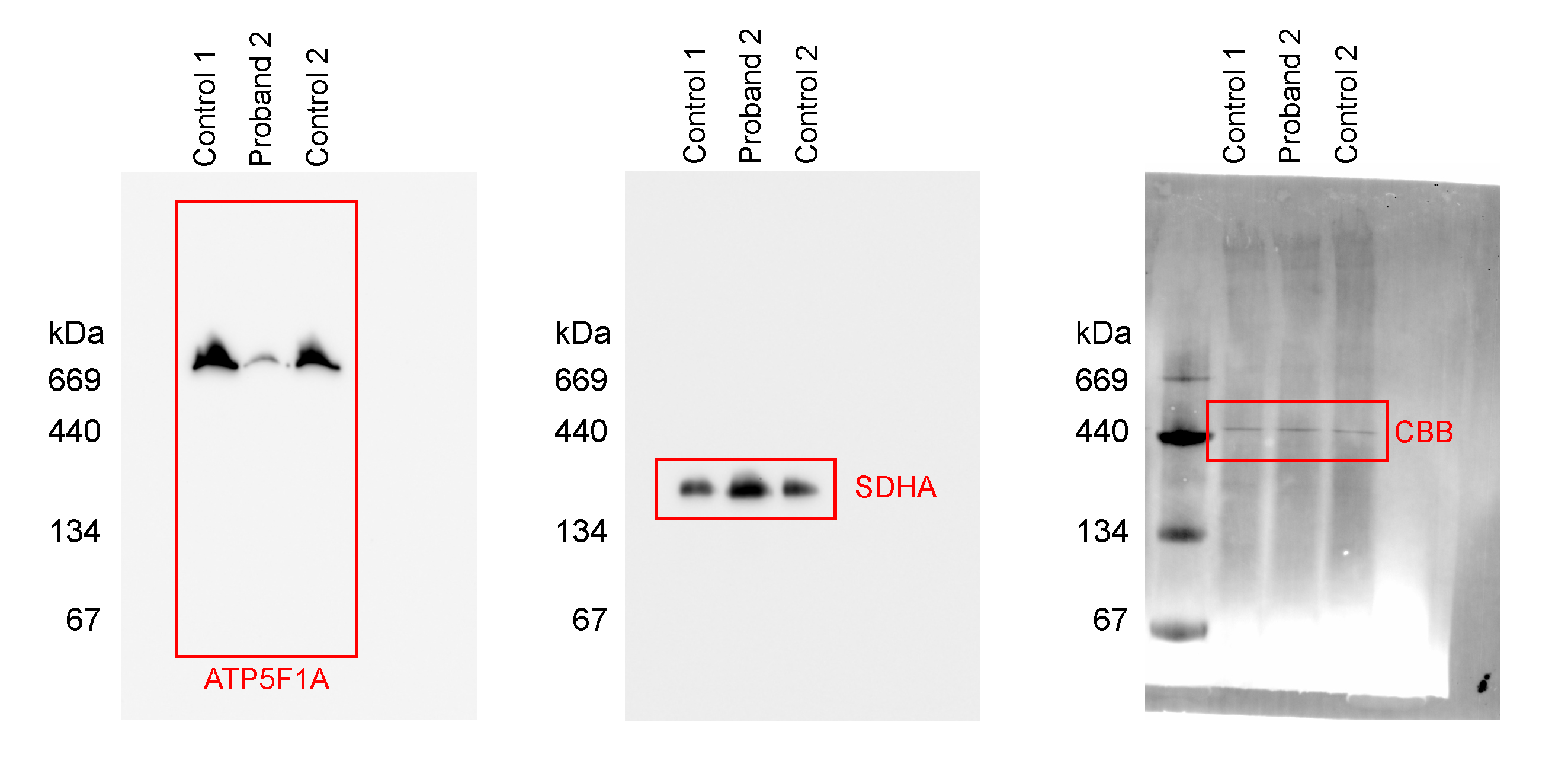

Supplement: Supplementary file 8 — Figure EV2 Source Data [file 44321_2025_290_MOESM8_ESM.zip › Figure EV2/Fig EV2B/Figure_EV2B_BNPAGE(unprocessed images).tiff]
