# Supplementary material for: Dominant negative ATP5F1A variants disrupt oxidative phosphorylation causing neurological disorders
Source: EMBO Mol Med. 2025 Aug 26;17(10):2562–85. doi: 10.1038/s44321-025-00290-8 (PMC12514044; doi:10.1038/s44321-025-00290-8)

Fig. 4E - All images were rotated and/or flipped horizontally to present the animals in the same orientation (i.e., the anterior to the left, and the vulva pointing down). Images were also cropped (red box) to reduce empty space. Brightness and contrast of the GFP images were not adjusted.


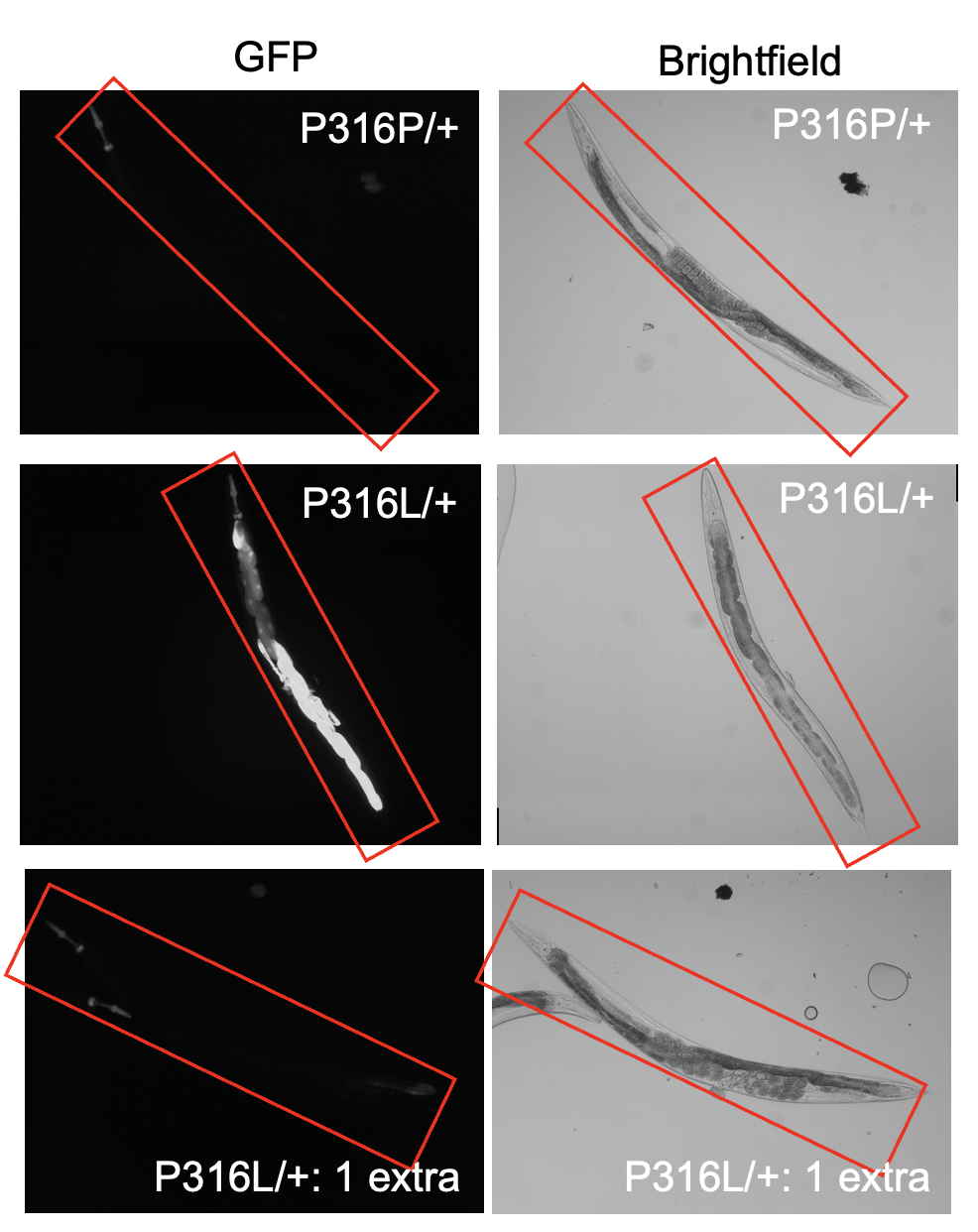

Supplement: Supplementary file 4 — Source data Fig. 4 [file 44321_2025_290_MOESM4_ESM.zip › Figure 4/Fig. 4E/Fig 4E_Readme.docx]
