# Supplementary material for: Dominant negative ATP5F1A variants disrupt oxidative phosphorylation causing neurological disorders
Source: EMBO Mol Med. 2025 Aug 26;17(10):2562–85. doi: 10.1038/s44321-025-00290-8 (PMC12514044; doi:10.1038/s44321-025-00290-8)
Supplement: Supplementary file 6 — Source data Fig. 6 [file 44321_2025_290_MOESM6_ESM.zip › Figure 6/Fig. 6C/Image cropping_Western blot.pptx]

## Slide 1
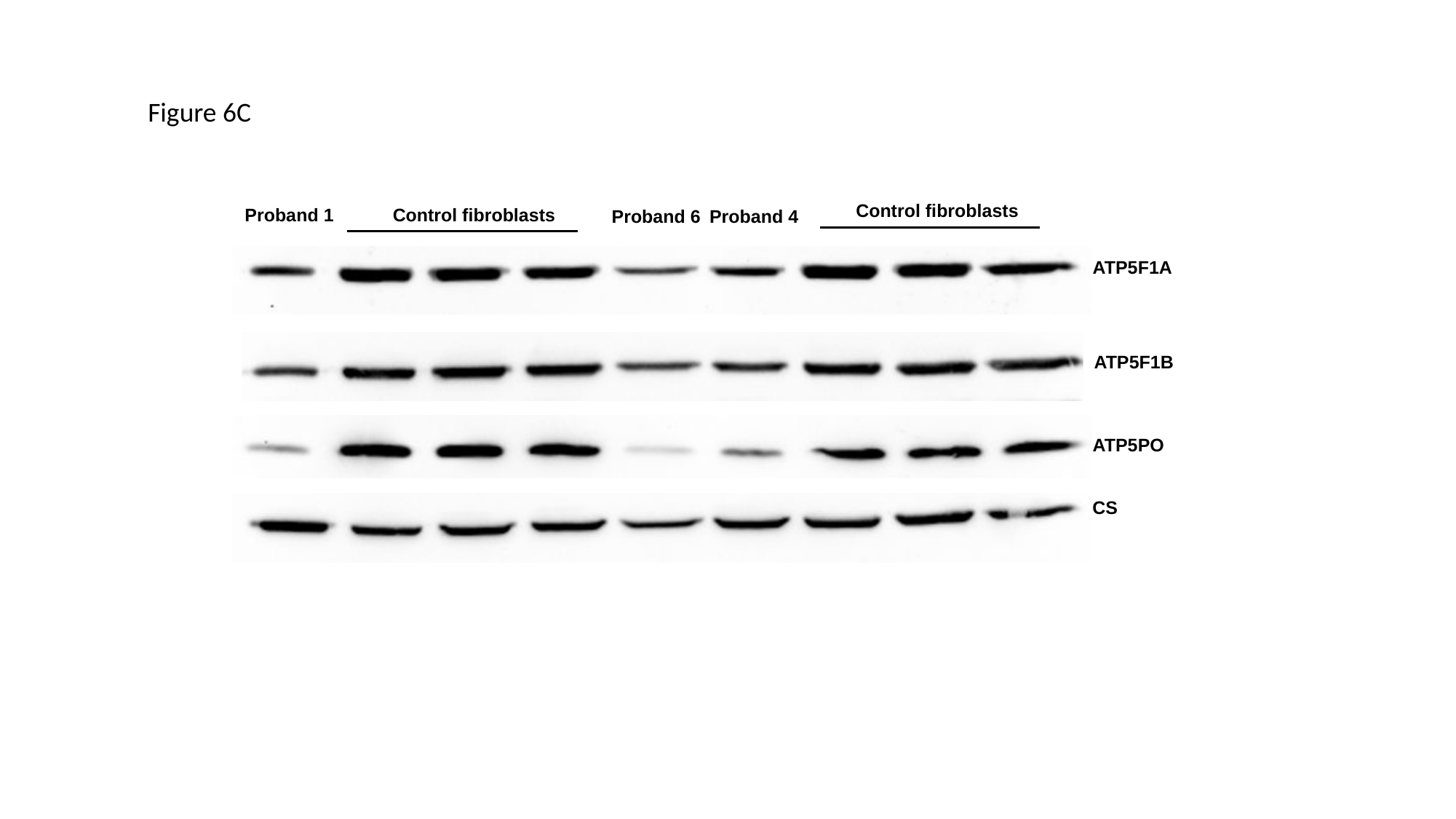

Figure 6C
Control fibroblasts
Control fibroblasts
Proband 1
Proband 6
Proband 4
ATP5F1A
ATP5F1B
ATP5PO
CS

## Slide 2
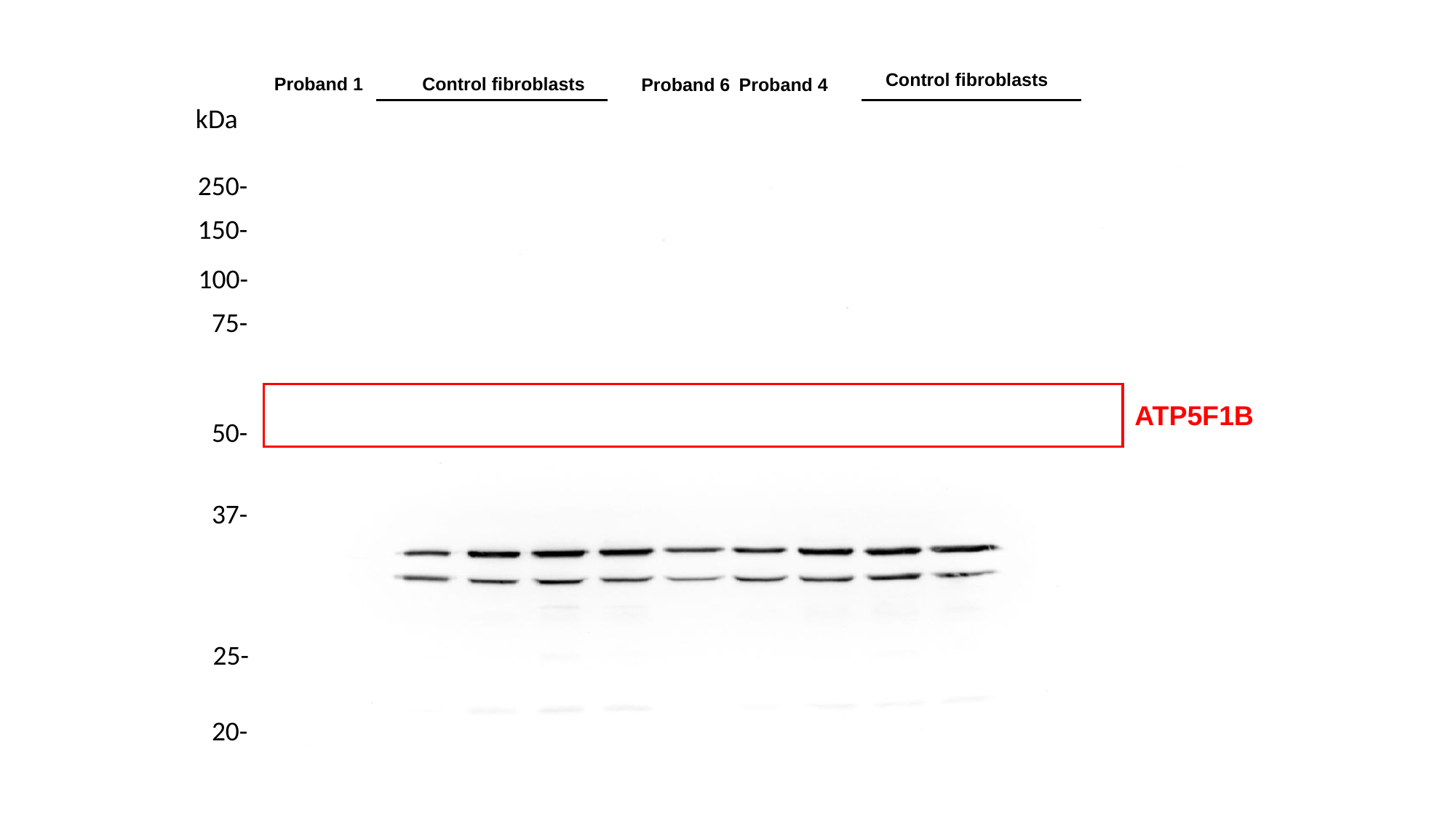

Control fibroblasts
Control fibroblasts
Proband 1
Proband 6
Proband 4
kDa
250-
150-
100-
75-
ATP5F1B
ATP5F1B
50-
37-
25-
20-

## Slide 3
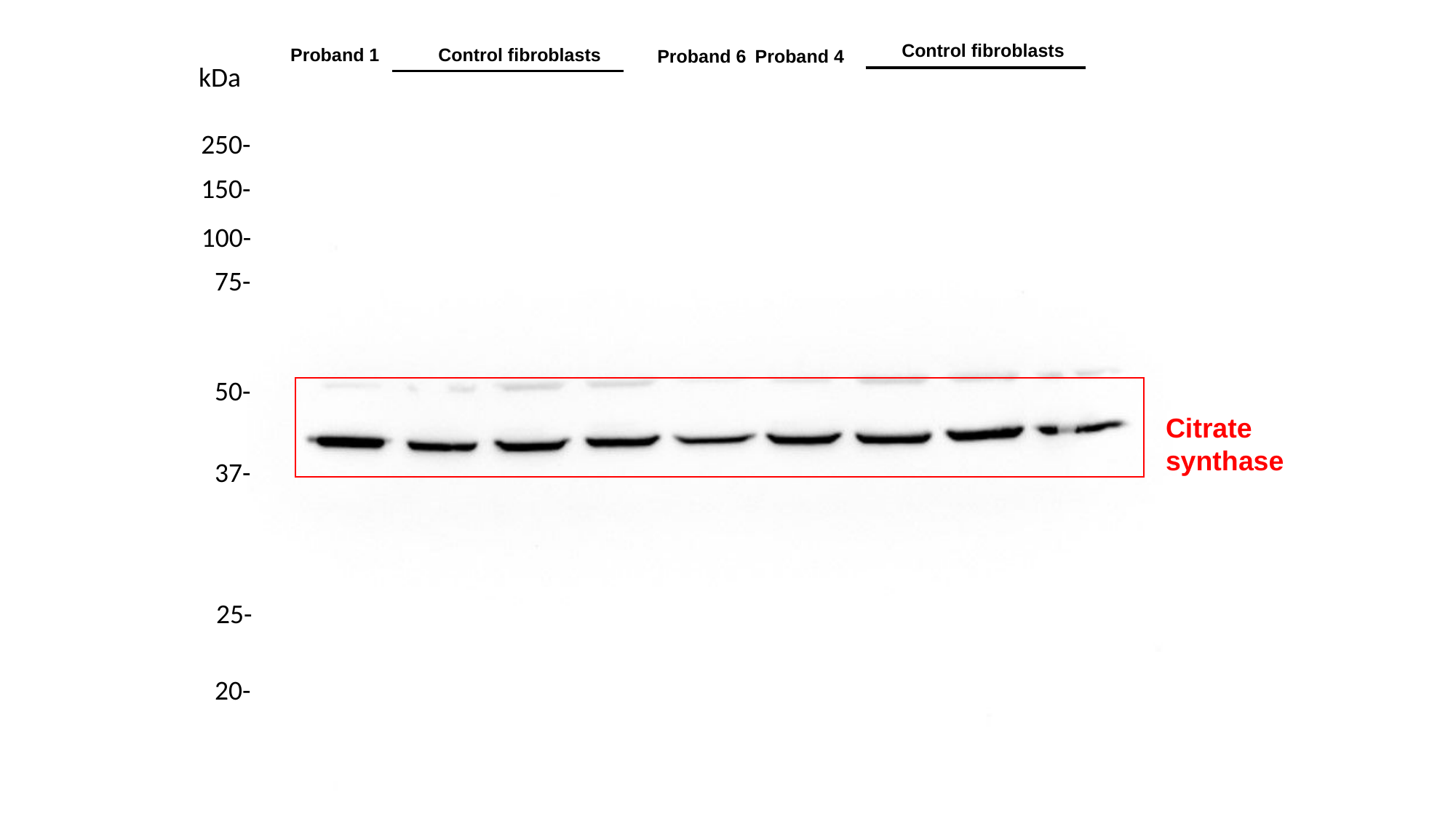

Control fibroblasts
Control fibroblasts
Proband 1
Proband 6
Proband 4
kDa
250-
150-
100-
75-
50-
Citrate synthase
37-
25-
20-

## Slide 4
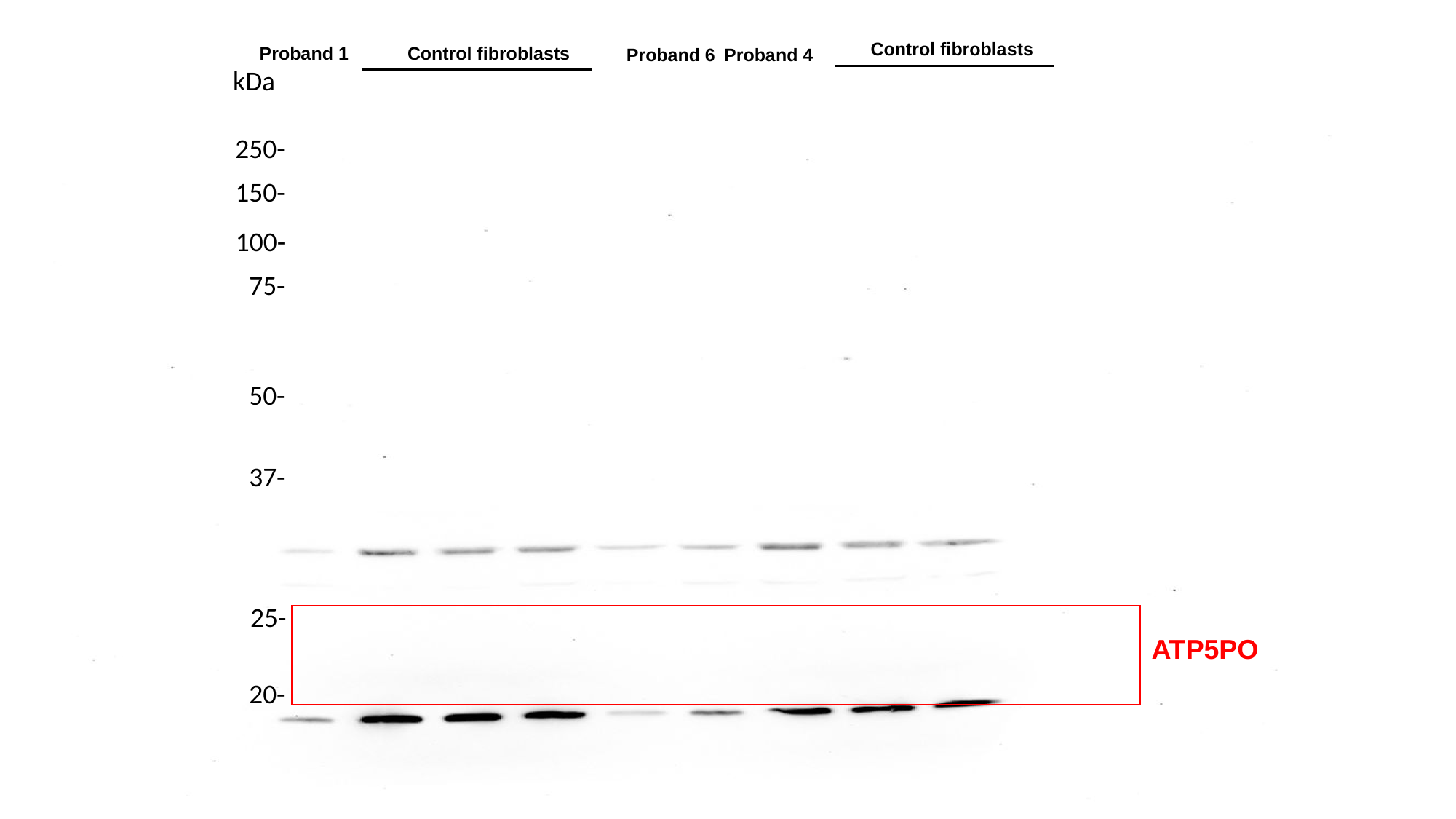

Control fibroblasts
Control fibroblasts
Proband 1
Proband 6
Proband 4
kDa
250-
150-
100-
75-
50-
37-
25-
ATP5PO
20-

## Slide 5
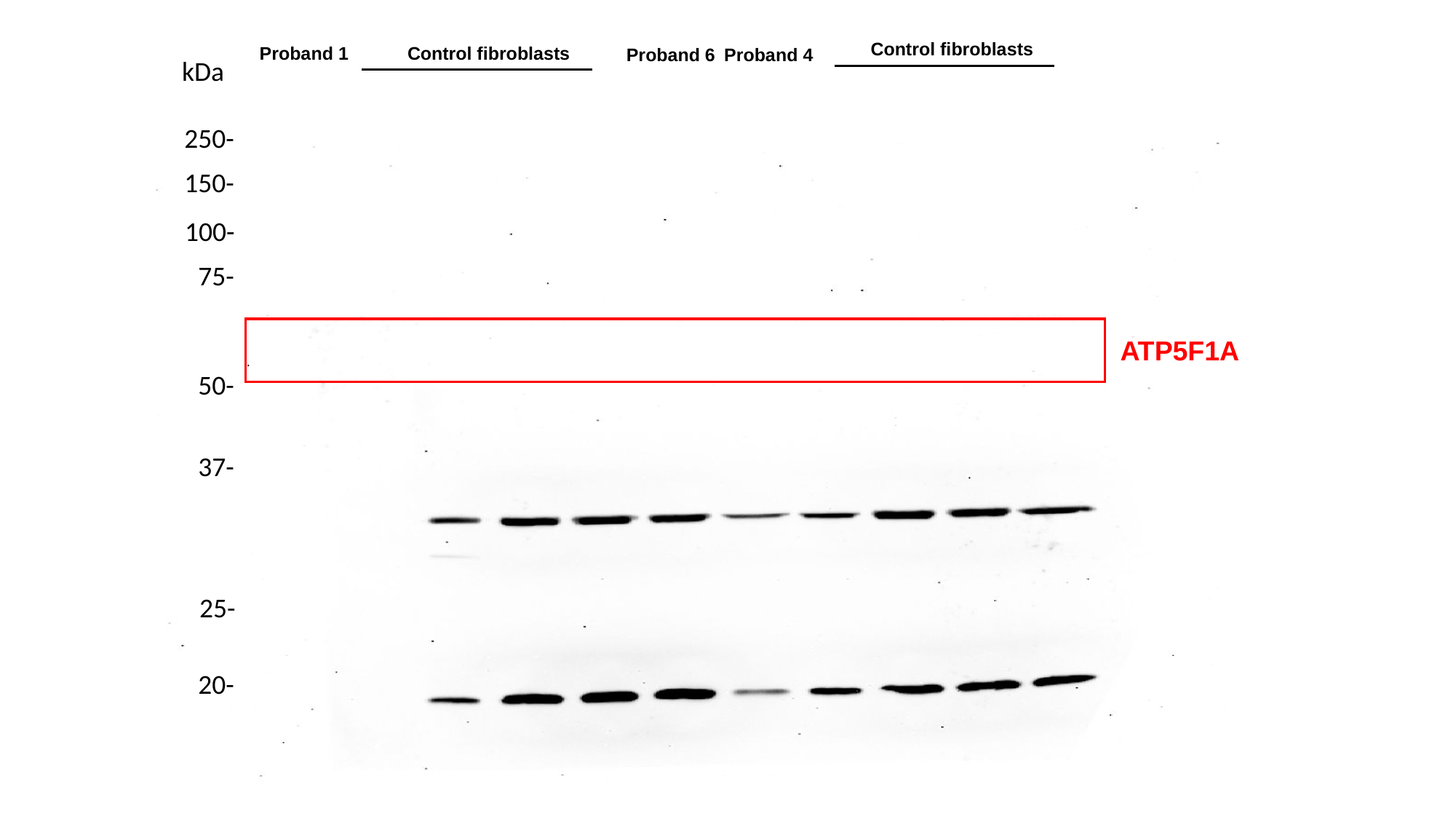

Control fibroblasts
Control fibroblasts
Proband 1
Proband 6
Proband 4
kDa
250-
150-
100-
75-
ATP5F1A
50-
37-
25-
20-

## Slide 6
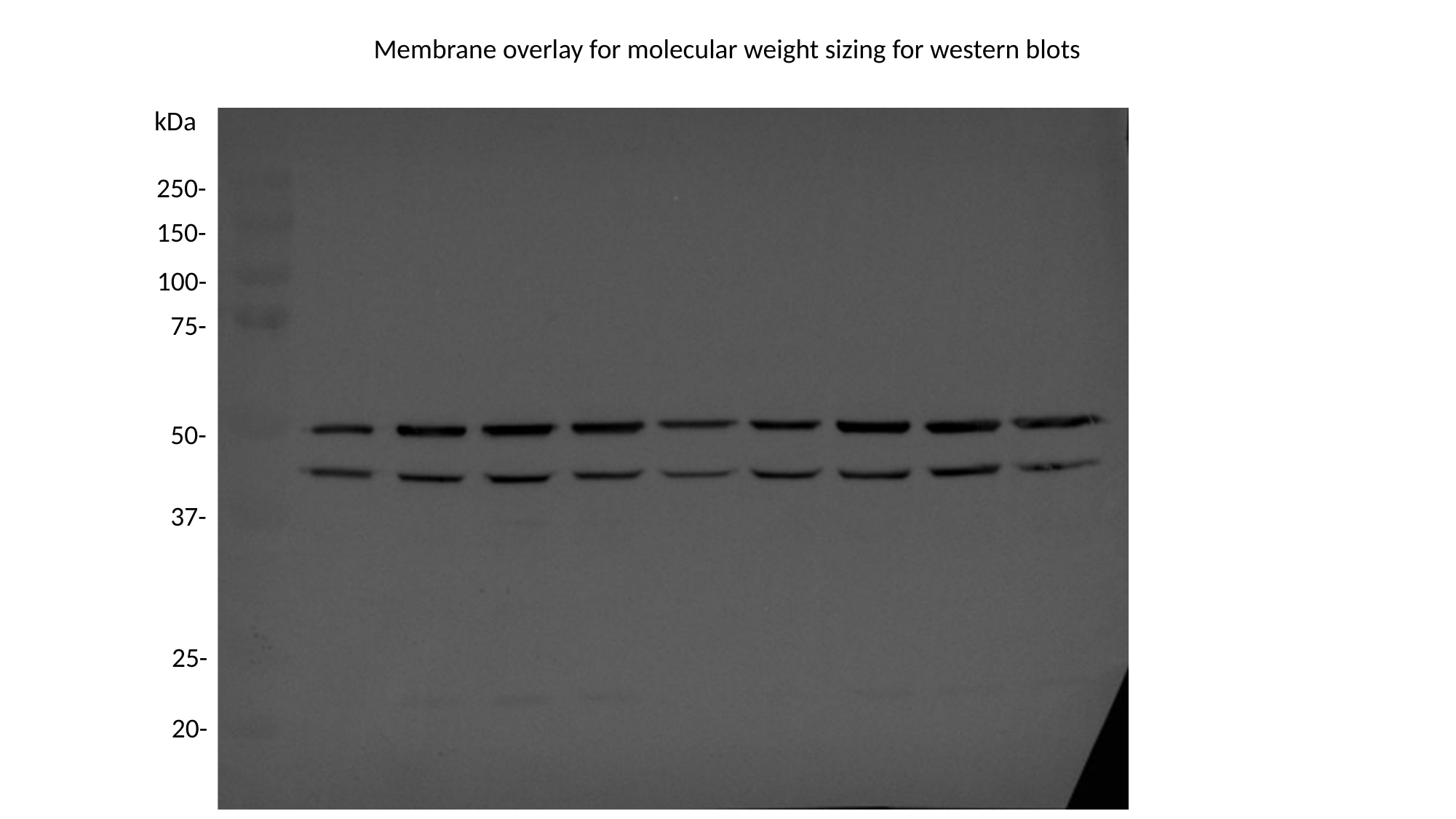

Membrane overlay for molecular weight sizing for western blots
kDa
250-
150-
100-
75-
50-
37-
25-
20-
