# Supplementary material for: Dominant negative ATP5F1A variants disrupt oxidative phosphorylation causing neurological disorders
Source: EMBO Mol Med. 2025 Aug 26;17(10):2562–85. doi: 10.1038/s44321-025-00290-8 (PMC12514044; doi:10.1038/s44321-025-00290-8)
Supplement: Supplementary file 6 — Source data Fig. 6 [file 44321_2025_290_MOESM6_ESM.zip › Figure 6/Fig. 6B/Image cropping_Proband 1.pptx]

## Slide 1
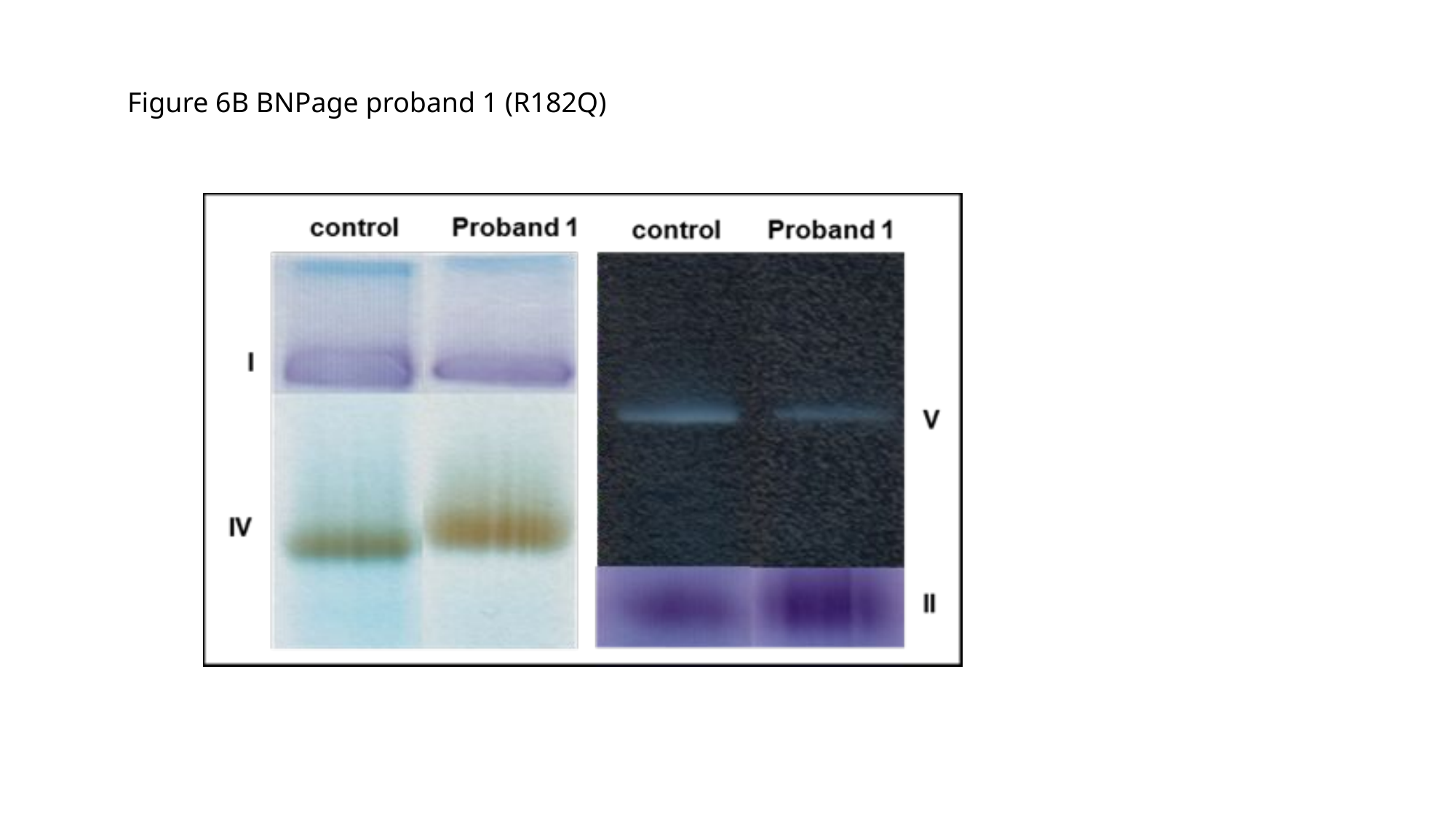

Figure 6B BNPage proband 1 (R182Q)

## Slide 2
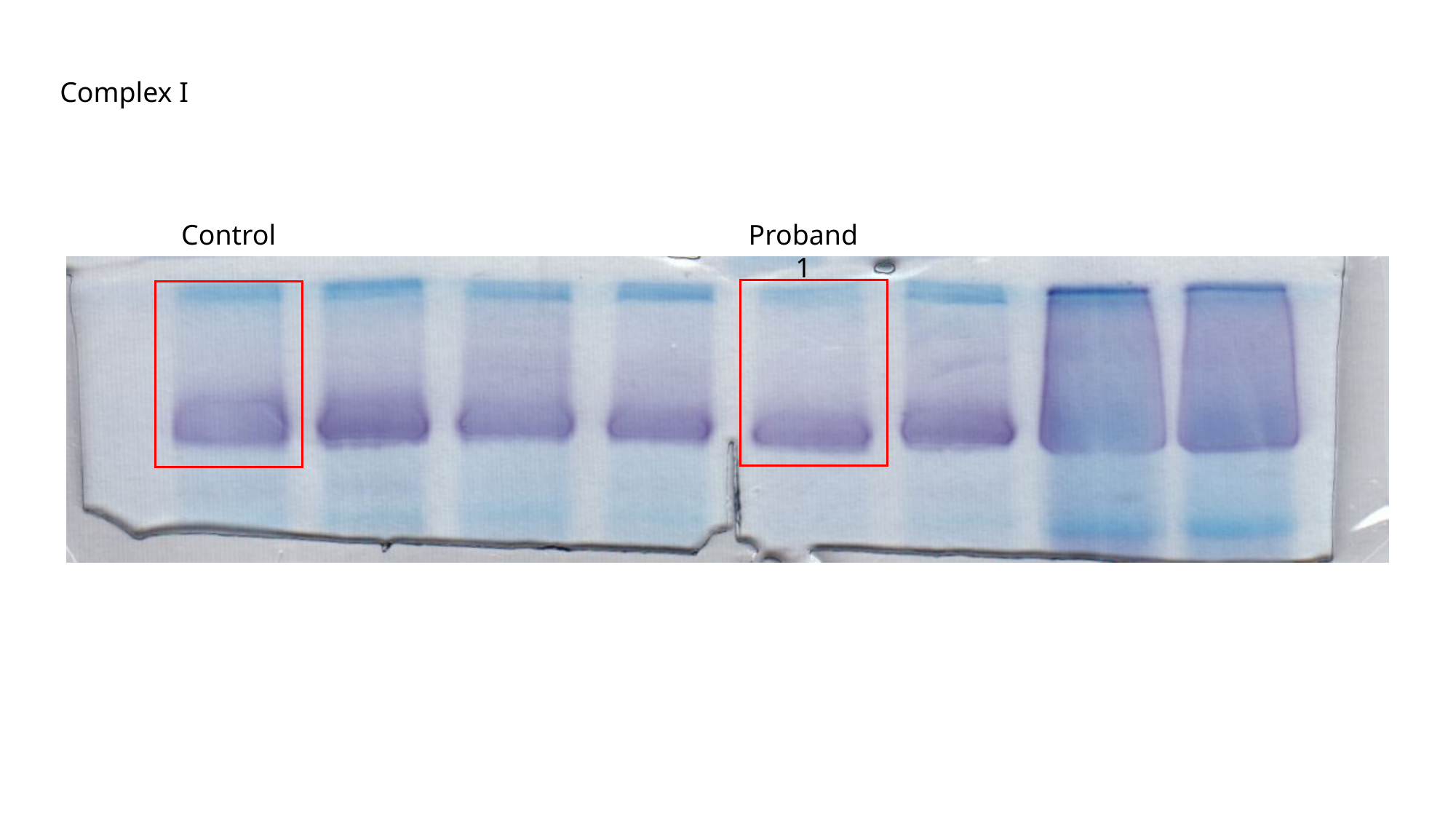

Complex I
Control
Proband 1

## Slide 3
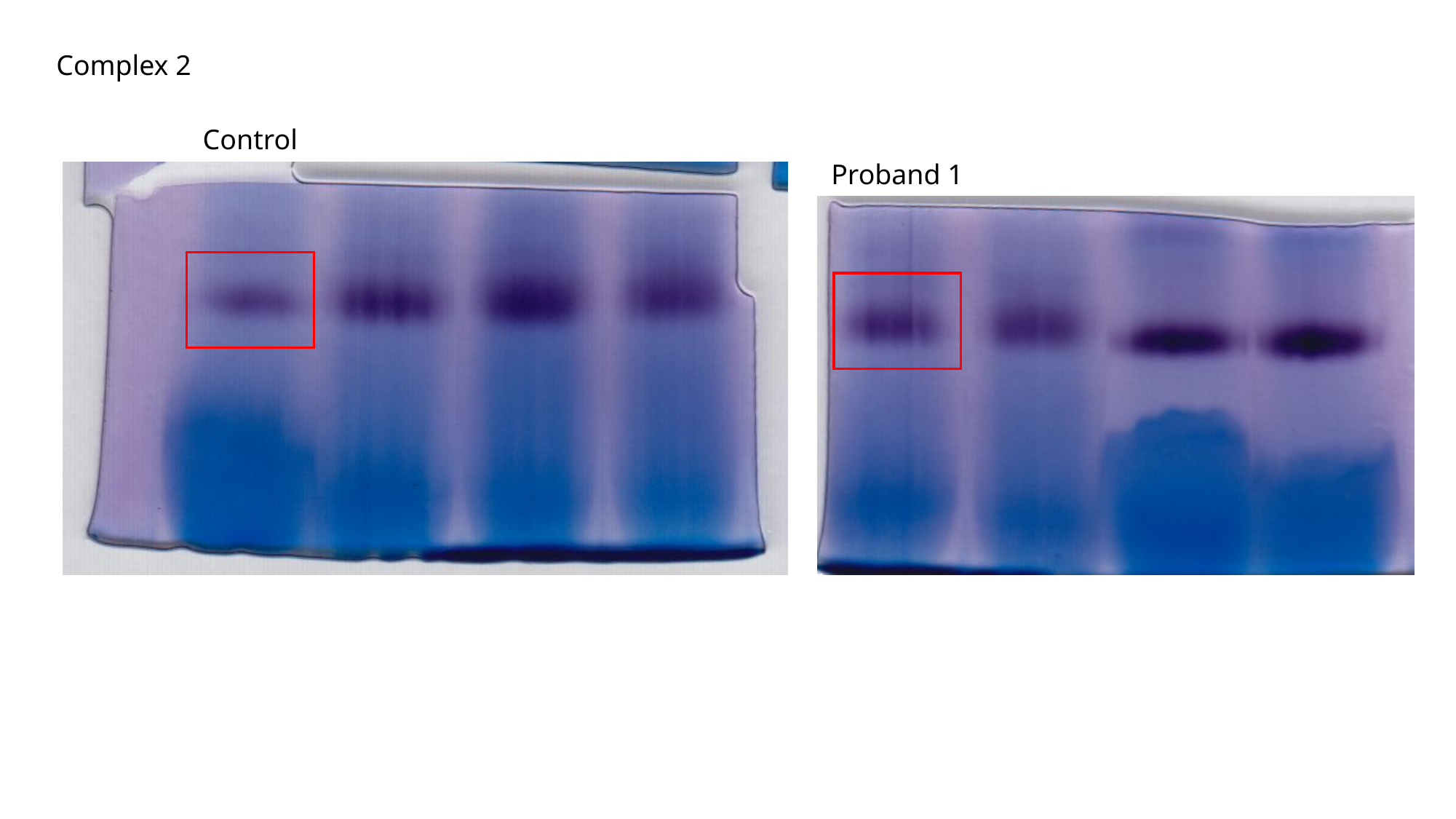

Complex 2
Control
Proband 1

## Slide 4
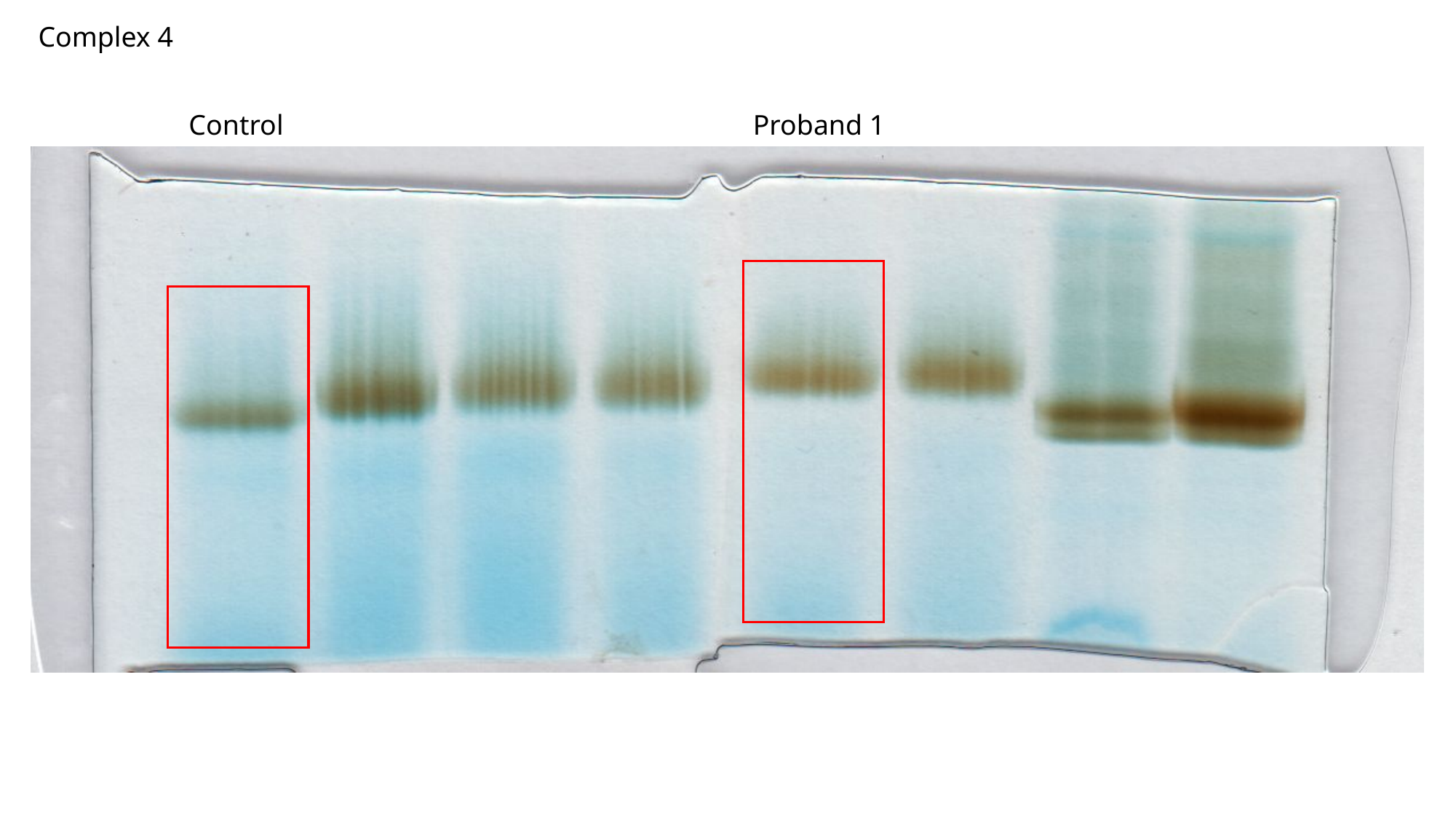

Complex 4
Control
Proband 1

## Slide 5
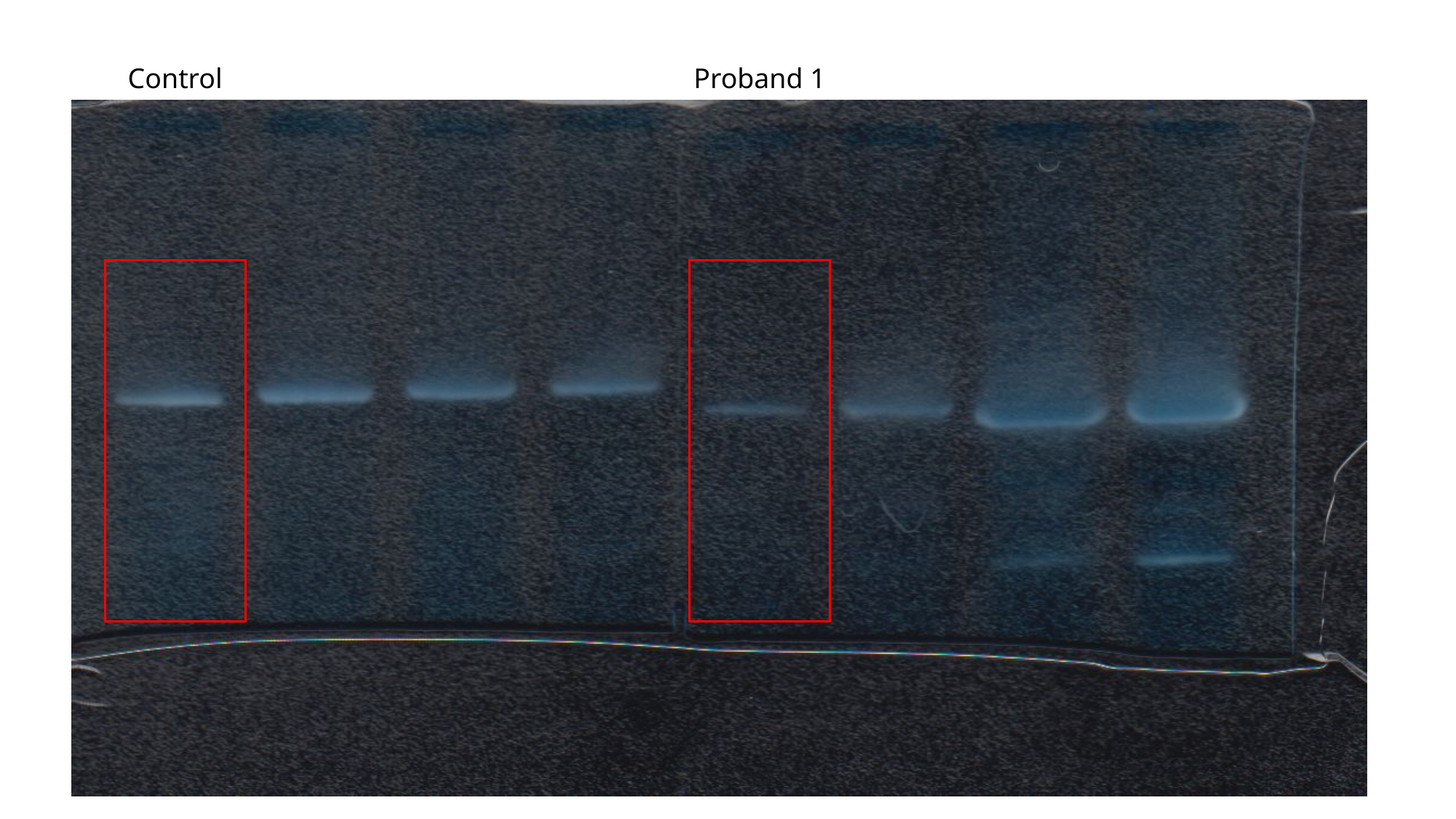

Control
Proband 1
