# Supplementary material for: Dominant negative ATP5F1A variants disrupt oxidative phosphorylation causing neurological disorders
Source: EMBO Mol Med. 2025 Aug 26;17(10):2562–85. doi: 10.1038/s44321-025-00290-8 (PMC12514044; doi:10.1038/s44321-025-00290-8)
Supplement: Supplementary file 8 — Figure EV2 Source Data [file 44321_2025_290_MOESM8_ESM.zip › Figure EV2/Fig EV2B/Figure_EV2B_README.docx]

The PVDF membrane was stained in Coomassie Brilliant Blue (CBB) and imaged to visualise the molecular weight markers and gel loading prior to immunoblotting. The membrane was then incubated in primary and secondary antibodies, followed by visualisation with Clarity Western ECL Substrate (BioRad) on a ChemiDoc XRS+ imaging machine (BioRad). Images were cropped for publication as outlined in source data for Fig. EV2B.
