# Supplementary material for: Dominant negative ATP5F1A variants disrupt oxidative phosphorylation causing neurological disorders
Source: EMBO Mol Med. 2025 Aug 26;17(10):2562–85. doi: 10.1038/s44321-025-00290-8 (PMC12514044; doi:10.1038/s44321-025-00290-8)
Supplement: Supplementary file 10 — Expanded View Figures [file 44321_2025_290_MOESM10_ESM.pdf]

Expanded View Figures

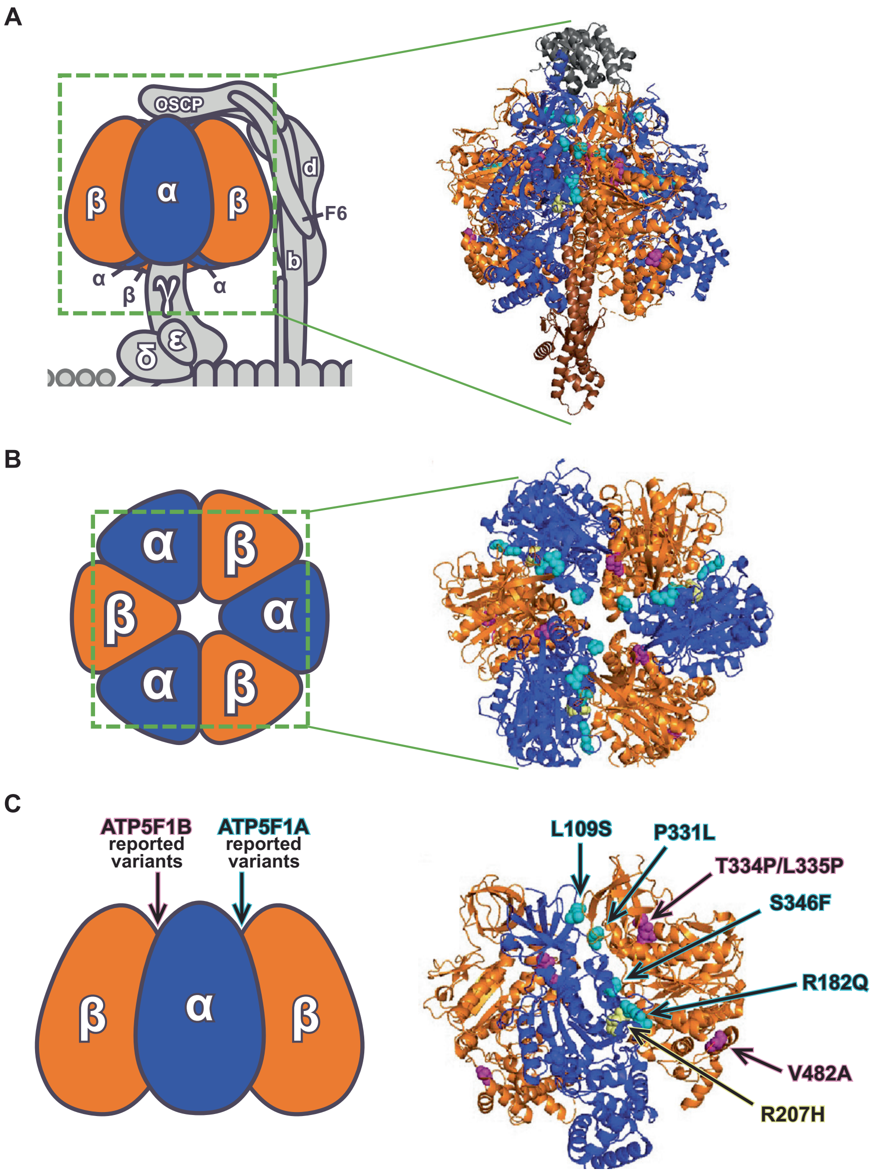

Figure EV1. 3D molecular modeling of dominant variants in ATP5F1A and ATP5F1B.

(**A**) Side and (**B**) top-down views of the cryo-structure of α- and β-subunits of ATP synthase. (**C**) A zoomed in view of the β:α:β subunits. The *ATP5F1A* variants (L109S, R182Q, P331L and S346F) from this study are shown in cyan, the previously published *ATP5F1A* variant (R207H) and *ATP5F1B* variants (T334P, L335P, V482A) are shown in yellow and magenta, respectively. Alignment, visualization, and mutagenesis were performed by using PyMOL (version 2.5.5) using previously described structural models comprising the 10-subunit human ATP synthase (PDB: 8H9S) (Lai et al, 2023).

© The Author(s)

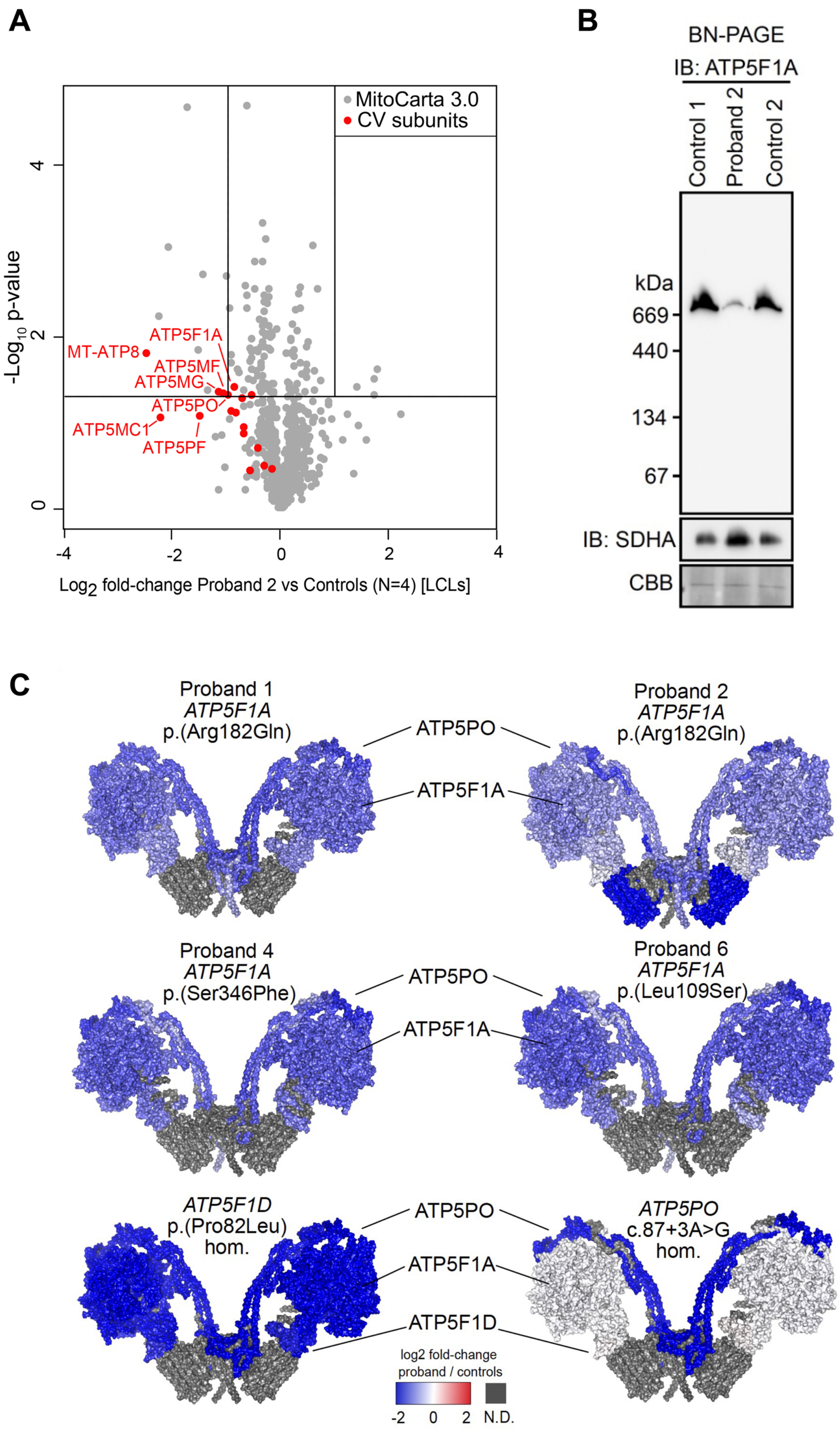
A
MitoCarta 3.0
CV subunits
-Log10 p-value
Log2 fold-change Proband 2 vs Controls (N=4) [LCLs]
ATP5F1A
MT-ATP8
ATP5MF
ATP5MG
ATP5PO
ATP5MC1
ATP5PF
B
BN-PAGE
IB: ATP5F1A
Control 1
Proband 2
Control 2
kDa
669
440
134
67
IB: SDHA
CBB
C
Proband 1
ATP5F1A
p.(Arg182Gln)
ATP5PO
ATP5F1A
Proband 2
ATP5F1A
p.(Arg182Gln)
Proband 4
ATP5F1A
p.(Ser346Phe)
Proband 6
ATP5F1A
p.(Leu109Ser)
ATP5F1D
p.(Pro82Leu)
hom.
ATP5PO
c.87+3A>G
hom.
ATP5F1D
log2 fold-change
proband / controls
-2 0 2 N.D.

Figure EV2. Proteomics analysis of mitochondrial proteins.

(**A**) Volcano plot of mitochondrial proteins annotated from MitoCarta3.0 of Proband 2 (p.R182Q) lymphoblastoid cell lines (LCLs) compared to controls ($N = 4$) showing reduced abundance of subunits of Complex V. Vertical lines represent ± 2-fold-change equivalent and horizontal lines represent significance P value $= 0.05$ equivalent from a two-sample t test. Red = Complex V subunits. (**B**) Blue native PAGE and immunoblotting (IB) of LCLs from Proband 2 and two unrelated controls against ATP5F1A and SDHA antibodies showing reduced abundance of complex V in Proband 2. CBB: Coomassie Brilliant Blue. (**C**) Topographical heatmap of the log2 fold-change abundances onto the cryo-EM structure of the dimer complex V structure for probands 1, 2, 4 and 6 as well as disease controls with known biallelic pathogenic variants in *ATP5F1D* and *ATP5PO* relative to controls. The topographical heatmaps are coloured using the fold-changes from the t-test results obtained in (**A**). Source data are available online for this figure.

 © The Author(s)
