# Supplementary material for: Dominant negative ATP5F1A variants disrupt oxidative phosphorylation causing neurological disorders
Source: EMBO Mol Med. 2025 Aug 26;17(10):2562–85. doi: 10.1038/s44321-025-00290-8 (PMC12514044; doi:10.1038/s44321-025-00290-8)
Supplement: Supplementary file 9 — Appendix Figure Source Data [file 44321_2025_290_MOESM9_ESM.zip › Appendix_Figure_S3.zip!/Appendix Figure S3/Appendix Fig. S3B/Appendix Fig. S3B_Readme.docx]

Fig. S2B - All images were rotated and/or flipped horizontally to present the animals in the same orientation (i.e., the anterior to the left, and the vulva pointing down). Images were also cropped (red box) to reduce empty space. Brightness and contrast was adjusted to highlight the internal organs and the age/size of the animal.
